# Stereoselective synthesis of sulfur-containing β-enaminonitrile derivatives through electrochemical Csp^3^–H bond oxidative functionalization of acetonitrile

**DOI:** 10.1038/s41467-019-08762-5

**Published:** 2019-02-19

**Authors:** Tian-Jun He, Zongren Ye, Zhuofeng Ke, Jing-Mei Huang

**Affiliations:** 1 Key Laboratory of Functional Molecular Engineering of Guangdong Province, School of Chemistry and Chemical Engineering, South China University of Technology, 510640 Guangzhou, China; 2 School of Materials Science and Engineering, PCFM Lab, Sun Yat-sen University, 510275 Guangzhou, China

## Abstract

Incorporation of nitrile groups into fine chemicals is of particular interest through C(sp^3^)–H bonds activation of alkyl nitriles in the synthetic chemistry due to the highly efficient atom economy. However, the direct α-functionalization of alkyl nitriles is usually limited to its enolate chemistry. Here we report an electro-oxidative C(sp^3^)–H bond functionalization of acetonitrile with aromatic/aliphatic mercaptans for the synthesis of sulfur-containing β-enaminonitrile derivatives. These tetrasubstituted olefin products are stereoselectively synthesized and the stereoselectivity is enhanced in the presence of a phosphine oxide catalyst. With iodide as a redox catalyst, activation of C(sp^3^)–H bond to produce cyanomethyl radicals proceeds smoothly at a decreased anodic potential, and thus highly chemoselective formation of C–S bonds and enamines is achieved. Importantly, the process is carried out at ambient temperature and can be easily scaled up.

## Abstract (web summary)

Direct α-functionalization of acetonitrile, a bulk chemical, is usually limited to its enolate chemistry. Here, the authors show an electro-oxidative C(sp^3^)–H bond functionalization of acetonitrile with thiols to afford tetrasubstituted olefins with high stereoselectivity.

## Introduction

**Fig. 1:**
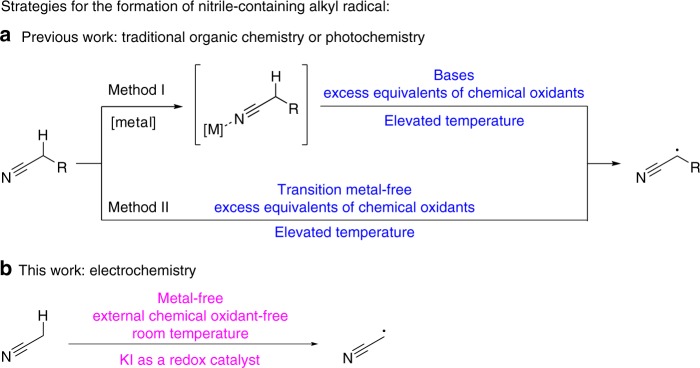
Strategies for the formation of nitrile-containing alkyl radicals. **a** The formation of nitrile-containing alkyl radicals under traditional organic chemical or photochemical conditions. **b** The formation of cyanomethyl radicals under electrochemical conditions

Nitriles are widely found in pharmaceuticals, natural products, and materials^1–4^. Introduction of nitrile groups onto target frameworks by C–H functionalization of C(sp^3^)–H bonds of simple aliphatic nitriles is of particular interest in the synthetic chemistry. Early research has focused on the C–H activation of alkyl nitriles using stoichiometric amounts of transition metal salts (such as Ru, Rh, Ni, Fe, etc.)^5–9^. On the other hand, the direct α-functionalization of alkyl nitriles is usually limited to its enolate chemistry, which requires a strong base for its formation^10–15^. Recently, free radical-initiated α-C–H functionalization of alkyl nitriles has attracted attention (Fig. 1a)^16,17^. Nevertheless, these methods require excess equivalents of strong oxidants (peroxides), metal-based oxidants (Ag^+^, Mn^3+^), or single-electron transfer reagents (diazonium salts). In addition, all of these works were carried out at elevated temperatures. Therefore, methods for the mild and environmentally friendly activation of alkyl nitriles are still highly desirable.

Electrochemical anodic oxidation represents an attractive and environment-friendly synthetic strategy to solve lingering problems in organic chemistry^18–33^. Particularly, the indirect electrolysis, in which a redox catalyst is utilized as the electron shuttle, achieves higher energy efficiency and different selectivity^34–43^. In continuation of our interest in the development of electrochemical methods to organic synthesis^44–47^, herein, we report the electrochemical C(sp^3^)–H bond oxidative functionalization of acetonitrile mediated by potassium iodide to synthesize sulfur-containing β-enaminonitrile derivatives highly efficiently in one pot (Fig. 1b).

## Results

### Reaction optimization

To initiate the investigation, *p*-fluorothiophenol (**1aa**) and acetonitrile were chosen as the substrates to test the reaction. Under a galvanostatic condition at 10 mA in an undivided cell, the reaction of **1aa** and acetonitrile with 10 mol% citric acid, 20 mol% 1,2-bis(diphenylphosphino)ethane (DPPE), and 50 mol% KI gave a 96% yield of the desired product as a pair of isomers of **2aa** (*Z/E* = 19:1, Table 1, entry 1) and a simple column chromatography separation could give the pure *Z*-isomer. Both the yield and stereoselectivity were reduced when the reaction was carried out in the absence of citric acid (Table 1, entry 2). The stereoselectivity decreased to 12:1 without DPPE (Table 1, entry 3). When the reaction was performed in the absence of KI, no desired product was obtained, which indicated that KI played a key role in this selective electro-oxidative reaction (Table 1, entry 4). Replacing citric acid with acetic acid led to a lower yield at 79% (Table 1, entry 5). No desired product was detected when *t-*BuOK was added instead of citric acid (Table 1, entry 6). When the temperature decreased to 10 °C, a 67% yield of **2aa** was found (Table 1, entry 7). Heating the reaction mixture to 50 °C or 70 °C resulted in poor stereoselectivities (Table 1, entries 8 and 9). Inferior reaction yield and stereoselectivity were obtained when *N*,*N*-dimethylformamide (DMF) was used as a co-solvent (Table 1, entry 10). The influence of the electrodes was also studied. Replacing either the Pt minigrid anode or the Pt wire cathode by a Pt foil led to a poor result (Table 1, entries 11 and 12). The increase of the electric current caused a lower yield and stereoselectivity (Table 1, entry 13). No desired product could be detected when the reaction was carried out at a current lower than 5 mA (Table 1, entry 14). Screening of acids or bases, redox catalysts, ligands, electrolytes, and electrode materials were also studied (See Supplementary Tables 1–5).

**Table 1:** Optimization of reaction conditions^a^


Entry	Variation from the standard conditions	Yield (%)^b^	*Z/E* ^b^
1	None	96	19:1
2	Without citric acid	44	10:1
3	Without DPPE	95	12:1
4	Without KI	0	
5	Acetic acid instead of citric acid	79	13:1
6	*t*-BuOK instead of citric acid	0	
7	10 °C	67	10:1
8	50 °C	99	12:1
9	70 °C	99	12:1
10	MeCN/DMF = 1:1	80	9:1
11^c^	Pt foil as an anode	71	9:1
12^c^	Pt foil as a cathode	66	9:1
13	15 mA, 3 h	84	13:1
14	5 mA, 8 h	0	

*DPPE* 1,2-bis(diphenylphosphino)ethane, *DMF* *N*,*N*-dimethylformamide, ^*19*^*F NMR* fluorine-19 nuclear magnetic resonance

^a^Standard conditions: **1aa** (0.5 mmol), citric acid (10 mol%), DPPE (20 mol%), KI (50 mol%), MeCN (5 mL), with 0.1 M *n*-Bu_4_NClO_4_ as electrolyte. A Pt minigrid electrode (52 mesh, 1 × 1.5 cm^2^) as an anode and a Pt wire (diameter = 0.5 mm, height = 2.0 cm) as a cathode, an undivided cell, constant current = 10  mA, 4  h, room temperature, 3.0 F mol^−1^

^b^Yields and *Z/E* ratios were determined by ^19^F NMR analysis of the crude reaction mixture using fluorobenzene as the internal standard

^c^ Pt foils (1.0 × 1.5 cm^2^)

### Substrate scope

**Fig. 2:**
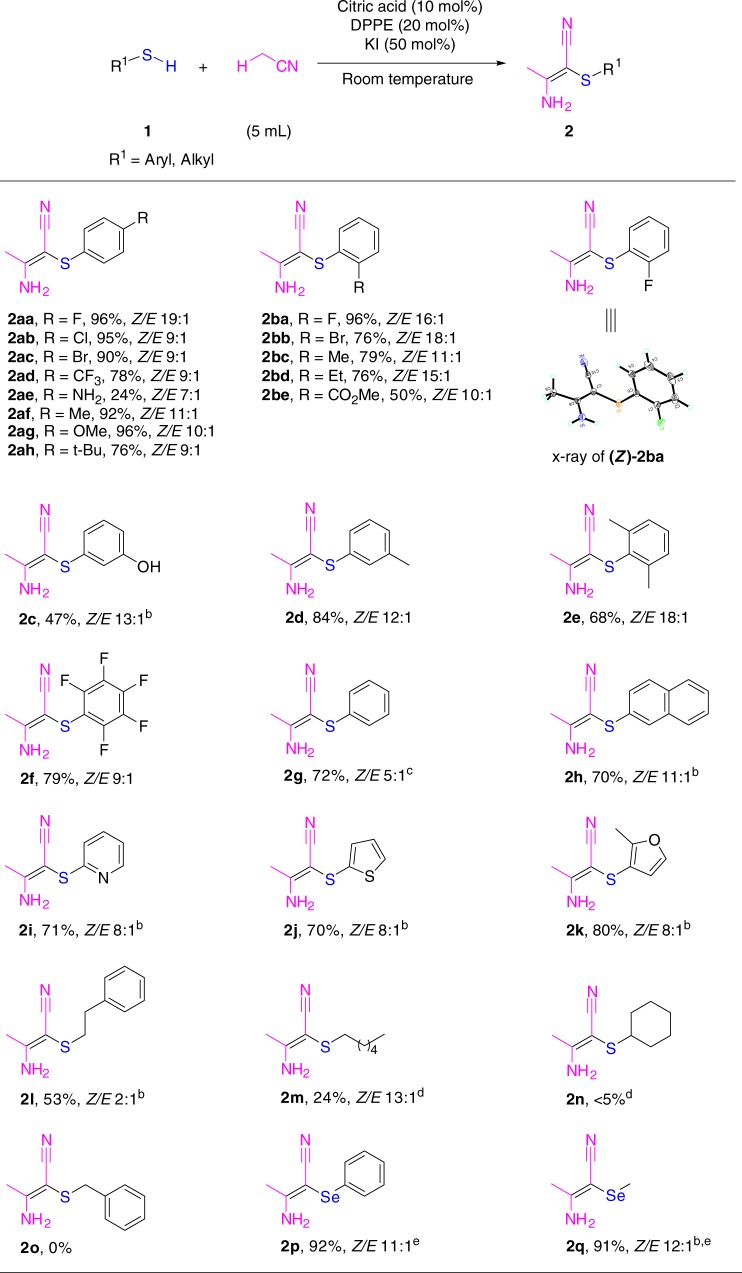
Substrate scope of thiols. ^a^Standard conditions: **1** (0.5 mmol), citric acid (10 mol%), 1,2-bis(diphenylphosphino)ethane (DPPE) (20 mol%), KI (50 mol%), MeCN (5 mL), with 0.1 M *n*-Bu_4_NClO_4_ as electrolyte. A Pt minigrid electrode as an anode and a Pt wire as a cathode, an undivided cell, constant current = 10 mA, 4 h, room temperature. Isolated yields are shown. *Z/E* ratios were determined by fluorine-19 nuclear magnetic resonance (^19^F NMR) or proton nuclear magnetic resonance (^1^H NMR analysis). ^b^KI (60 mol%), reaction time: 6 h. ^c^Phenyl disulfide as a substrate. ^d^KI (60 mol%), reaction time: 10 h. ^e^Diselenides as substrates

With the optimized conditions defined (Table 1, entry 1), the scope of phenylthiols/thiols was probed. As shown in Fig. 2, the reactions of various phenylthiols/thiols proceeded smoothly and the desired products were obtained in good to excellent yields with good stereoselectivities in most cases. First, the reactivity of phenylthiols with substituents on the benzene ring was studied. In general, both electron-donating and electron-withdrawing groups with different substitution patterns (*para-*, *meta-*, and *ortho-* substitutions; mono and multi substitutions) were tolerated in this reaction. Aryl thiols bearing fluoro, chloro, bromo, methyl, and methoxy groups could give the desired products in excellent yields (90–96%) and good selectivities. In addition, other fluorine-containing phenylthiols, such as trifluoromethyl phenylthiol and pentafluoro phenylthiol, were compatible with this protocol, affording **2ad** and **2f** in 78 and 79% yields, respectively. Somewhat steric effects were observed with the functional groups on the *ortho*-position (**2bb**–**2be**). Interestingly, oxidatively labile functional groups, such as amino and hydroxy, were tolerated in this transformation to produce the corresponding products (**2ae** and **2c**) in 24 and 47% yields, respectively. The present method could also be applied to diphenyl disulfide (**2g**).

Naphthyl and heteroaromatic thiols (**2** **h**, **2i**–**2k**) were effective for this reaction. Notably, the substrate scope could also be extended to alkyl mercaptans. The primary thiols, 2-phenylethanethiol and 1-hexanethiol, could be transformed to the corresponding products (**2l** and **2m**) in 53 and 24% yields, respectively. The secondary thiol, that is, cyclohexanethiol gave the product (**2n**) in a yield of 5% only. Unfortunately, no **2o** was detected when benzyl mercaptan was used as the reactant. Finally, it was delightful to find that diphenyl diselenide (**2p**) and dimethyldiselenide (**2q**) could be converted effectively to the corresponding products in excellent yields and good selectivitities.

The absolute stereochemistry of one of the products **(*Z*)-2ba** was determined by X-ray crystallographic analysis (Fig. 2). The stereochemistry of other products was determined on the basis of the similarities of the polarities and the ^1^H NMR and ^13^C NMR chemical shifts.

**Fig. 3:**
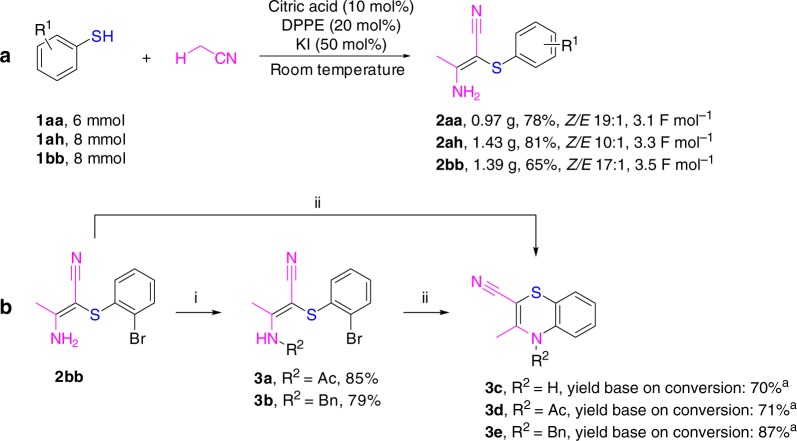
Gram-scale synthesis and product transformations. Reaction conditions: (i) acetyl chloride, Et_3_N, CH_2_Cl_2_, 0 °C to reflux, 12 h, 85%; BnBr, NaH, dry *N*,*N*-dimethylformamide (DMF), N_2_, 0 °C to r.t., 4 h, 79%. (ii) CuI, K_2_CO_3_, *trans*-*N,N′*-dimethylcyclohexane-1,2-diamine, *N,N′*-dimethylethylenediamine, toluene, N_2_, 120 °C, 15 h, conditions to be optimized. ^a^Conversions: **3c**, 60%; **3d**, 72%; **3e**, 62%

Next, the synthetic utility of this methodology was further investigated. First, the gram-scale synthesis of **2aa**, **2ah**, and **2bb** was performed and the desired products were obtained in the yields of 78%, 81%, and 65%, respectively. Second, 4*H*-1,4-benzothiazine scaffolds were obtained by copper-catalyzed cyclization of **2bb** and the corresponding derivatives (**3a** and **3b**) in the conversion yields of 70–87%. Notably, 4*H*-1,4-benzothiazine scaffolds are widely used in pharmaceutical chemistry due to their activities of antimicrobial, anticancer, and so on (Fig. 3).

## Discussion

**Fig. 4:**
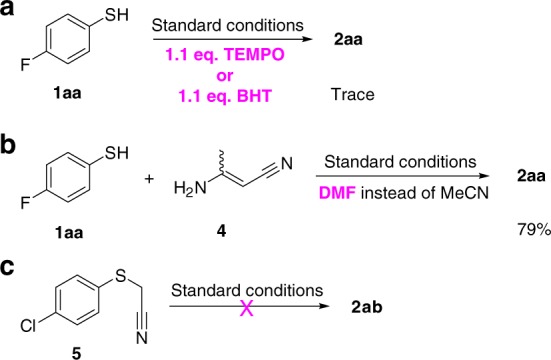
Mechanistic studies on the reaction. **a** Reactions by adding radical scavengers (tetramethylpiperidine N-oxyl (TEMPO) or butylated hydroxytoluene (BHT)). **b** The reaction between **1aa** and **4**. **c** The reaction between **5** and acetonitrile

Further studies were carried out to gain more insights into the reaction mechanism. First, when a radical scavenger, TEMPO (tetramethylpiperidine N-oxyl) or BHT (butylated hydroxytoluene), was added into the reaction mixture under the standard conditions, only a trace of desired product **2aa** was detected (Fig. 4a), and thus a radical nature of the transformation was implied. On the other hand, 3-aminocrotononitrile **4** was detected by gas chromatography–mass spectrometry (GC−MS) analysis during the above two reaction processes. Subsequent investigation demonstrated that the reaction started from **4** could produce the desired product **2aa** (Fig. 4b), while no **2ab** was obtained from (4-chlorophenylthio)acetonitrile **5** (Fig. 4c). Therefore, it was confirmed that the first step of this tandem reaction is an acetonitrile self-condensation to produce **4**.

**Fig. 5:**
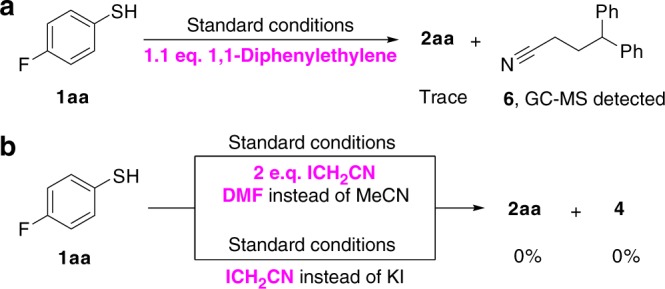
Studies on the pathway for the formation of intermediate **4**. **a** Radical trapping experiment by 1,1-diphenylethylene. **b** The reaction between **1aa** and iodoacetonitrile

Next, the formation of **4** was investigated. The pH value was monitored and it showed that the pH value increased steadily from 2 to 6 (see Supplementary Figure 1). Obviously, it was not a traditional Thorpe-type self-condensation through the ^−^CH_2_CN, which usually occurs under strongly basic conditions^48–50^. On the other hand, a radical trapping adduct, **6**, was detected (GC−MS analysis) by the use of 1,1-diphenylethene as the radical inhibitor, which suggested the intermediacy of cyanomethyl radical (Fig. 5a). Notably, the reaction did not proceed to afford the desired product **2aa** when acetonitrile was replaced by iodoacetonitrile (Fig. 5b). Moreover, using iodoacetonitrile instead of KI could not generate the desired product **2aa** even if acetonitrile was used as a solvent (Fig. 5b). These results rule out the formation of the intermediate ICH_2_CN in the early stage of the reaction.

**Fig. 6:**
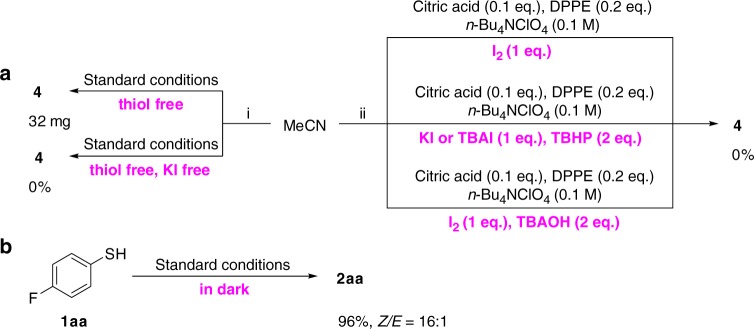
Studies on the active catalytic species for the formation of intermediate **4**. **a** I. Studies on the formation of **4**; II. the exploration of the iodine species. **b** The standard reaction run in dark

No desired reaction occurred in the absence of KI under the standard conditions (Table 1, entry 4). Further studies showed that in the absence of thiol, **4** could be obtained (Fig. 6a), while no **4** was detected without the addition of KI into the above reaction mixture. It demonstrated that KI played a crucial role in the formation of **4**. It was observed that the production of **4** needed the galvanic current, but the standard reaction could proceed in the dark (Fig. 6b). Hence, the formation of **4** might have been catalyzed by an iodine species that was generated by anodic oxidation, instead of photoexcitation, from KI^51,52^. Next, to explore the actual iodine species in the reaction, several different stoichiometric amounts of iodine sources were employed in the model reaction without the current. No **4** could be detected when I_2_ was applied (Fig. 6a). It has been reported that quaternary ammonium hypoiodite [*n*-Bu_4_N]^+^[IO]^−^ or iodite [*n*-Bu_4_N]^+^[IO_2_]^−^ could abstract a hydrogen atom from a C(sp^3^)−H to produce a radical^53^. However, the subsequent investigations using KI/TBHP, TBAI/TBHP, or I_2_/TBAOH system, which have been reported to generate hypoiodite or iodite, gave no **4** (Fig. 6a). Thus, we hypothesized that the active iodine radical^54–58^, which was in situ generated from the anodic oxidation, was able to abstract one hydrogen atom from acetonitrile to form the cyanomethyl radical.

**Fig. 7:**
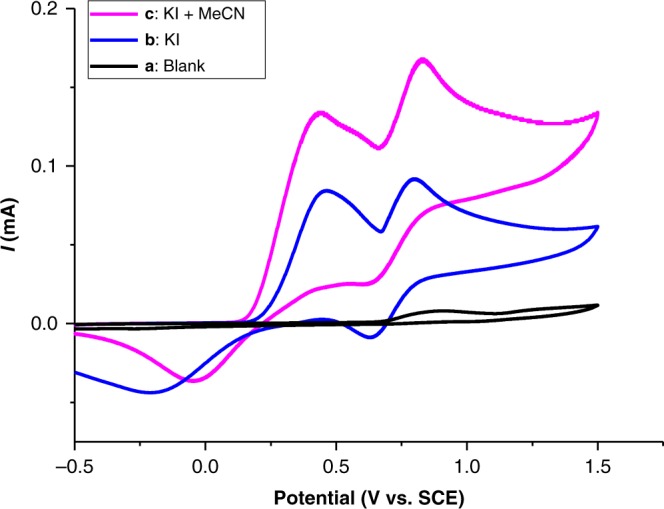
Cyclic voltammograms of 0.1 M *n*-Bu_4_NClO_4_ solution in *N*,*N*-dimethylformamide (DMF) at room temperature. **a** None; **b** KI (50 mmol L^−1^); **c** KI (50 mmol L^−1^) + MeCN (1 mL). The voltammogram was obtained with Pt wire as an auxiliary electrode and a saturated calomel electrode (SCE) as a reference electrode. The scan rate was 0.1 V s^−1^ on a platinum disk electrode (*d* = 2 mm)

Cyclic voltammetry studies (see Supplementary Figure 2) showed that KI (Fig. 7, curve b) exhibited two pairs of typical redox waves, with the oxidation peaks at 0.46 V (Ox_1_) and 0.80 V (Ox_2_) vs. SCE. After acetonitrile was introduced, obvious catalytic currents were detected; the peak currents of Ox_1_ and Ox_2_ dramatically increased from 84 to 134 and 92 to 168 μA, respectively (Fig. 7, curve c). Therefore, it was suggested that KI was employed as a redox catalyst in this indirect electrolysis process.

**Fig. 8:**
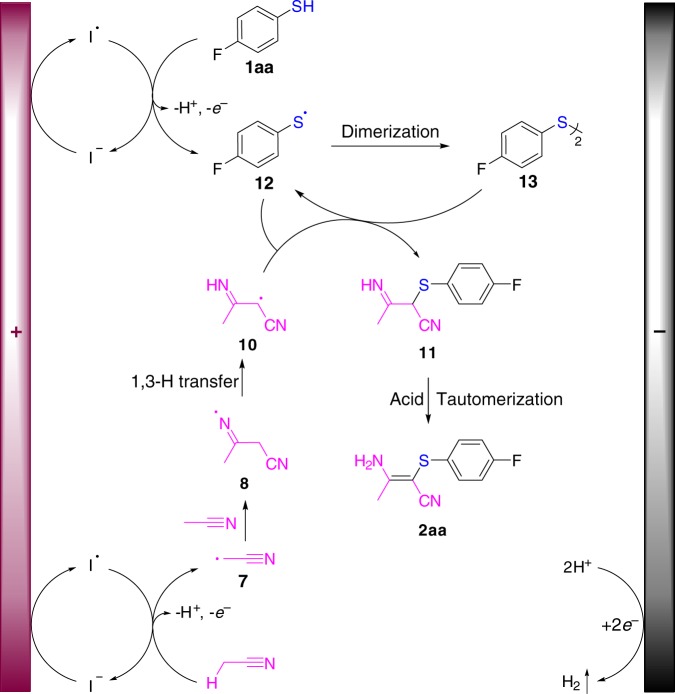
Proposed mechanism. Proposed reaction mechanism involves indirect anode oxidation of acetonitrile to cyanomethyl radical **7**, addition to acetonitrile, 1,3-H transfer to produce **10**, reaction of **10** with **12** or **13**, and tautomerization to furnish the final product **2aa**

On the basis of these above results, a plausible mechanism was proposed (Fig. 8). The reaction sequence began with the in situ generation of an iodine radical on the anode and the iodine radical abstracted one hydrogen atom from acetonitrile to form the cyanomethyl radical **7**. Addition of **7** to another molecule of acetonitrile furnished intermediate **8**^59^. The α-imine radical intermediate **10** was obtained by a 1,3-hydrogen transfer^60–63^ of iminyl radical **8**. Meanwhile, thiol **1aa** could be oxidized by the redox catalyst or by the anode directly to afford a sulfur radical **12**, which underwent dimerization to generate a disulfide **13**^64–66^. Thus, radical intermediate **10** could substitute with the disulfide **13** or couple with the sulfur radical **12** directly to produce imine **11**, which could tautomerize to give the desired product **2aa** in the presence of the acid catalyst. However, another pathway cannot be ruled out. Tautomerization of **10** to the corresponding enamine radical **9** was followed by the substitution with the disulfide **13** or the coupling with the sulfur radical **12** directly to form the desired product **2aa**. Concomitantly, cathodic reduction of protons led to the release of H_2_.

**Fig. 9:**
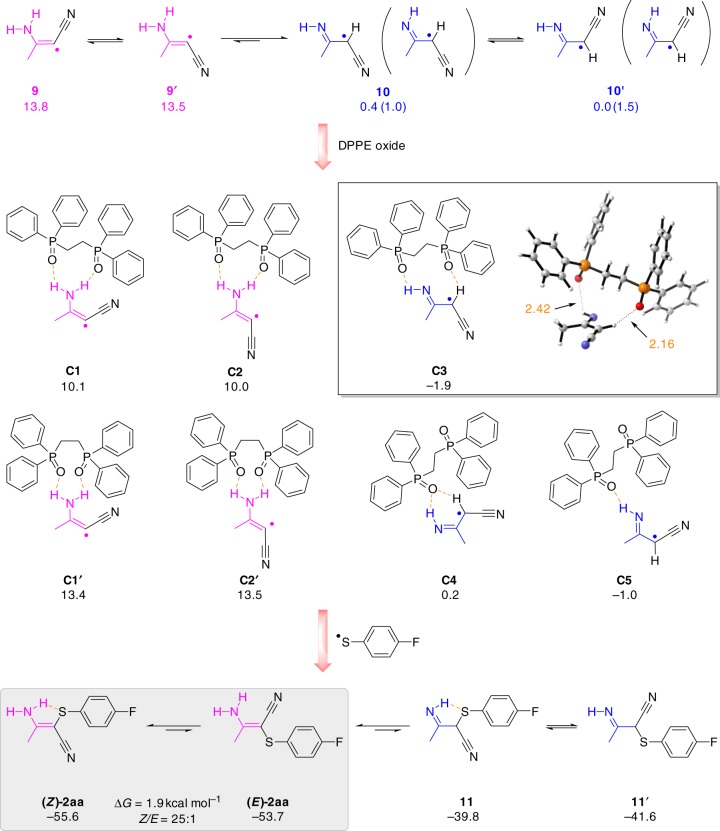
Density functional theory (DFT) study of the key intermediates and the calculated structures (Å, red) for complex **C3**. DFT study shows imine radical **10** can be coordinated with 1,2-bis(diphenylphosphino)ethane (DPPE) oxide to form complex **C3** via hydrogen bonding. The result suggests the important role of DPPE oxide in stabilizing the imine radical **10**, facilitating the formation of *Z* product and its tautomerization to final product **(*Z*)-2aa**

It was observed that the selectivity increased when DPPE was added. Studies showed that DPPE was in situ oxidized to 1,2-ethylene bis(diphenylphosphine oxide) on the anode (see Supplementary Discussion). Pre-oxidation of DPPE on the anode before the main reaction occurred the same yield and selectivity. We further carried out a density functional theory (DFT) calculations to provide insights into the mechanism (Fig. 9). DFT results indicate that imine type radical intermediates (**10** or **10′**) are more stable than the enamine type radicals (**9** or **9′**). The imine radical would interact with DPPE oxide to form complexes **C3**–**C5**, among which **C3** is the most stable one with the calculated formation energies of –1.9 kcal mol^−1^. The complex **C3** can stabilize the yielded radical and facilitate the C–S bond formation in *Z* configuration. It should be noted that the enamine type products (**2aa**) are more stable than imine types (**11** and **11′**). Therefore, the formed imine products would tautomerize to give the desired product **2aa**, in which the **(*Z*)-2aa** is more stable than the **(*E*)-2aa** by 1.9 kcal mol^−1^. The predicted *Z*:*E* is around 25:1, which is in excellent agreement with our experimental observation (19:1). Considering the relative stability between **(*Z*)-2aa** and **(*E*)-2aa**, the reaction should be thermodynamic control. DFT results suggest the important role of DPPE oxide in stabilizing the imine radical **10**, facilitating the formation of *Z* product and its tautomerization to final product **(*Z*)-2aa**.

In conclusion, we have developed a radical-initiated C(sp^3^)–H bond oxidative functionalization of acetonitrile through a KI-mediated indirect anodic oxidation. A wide range of aromatic/aliphatic mercaptans bearing various functional groups could participate in the reactions with acetonitrile to afford sulfur-containing β-enaminonitrile derivatives with concomitant generation of (*Z*)-tetrasustituated olefins. The high chemoselectivities and good stereoselectivities of the reactions could be achieved under metal-free, external chemical oxidant-free conditions. Further investigations into the mechanistic details and synthetic applications are currently underway in our laboratory.

## Methods

### Representative procedure for the synthesis of **2aa**

Into a round bottom flask was added thiol **1aa** (0.5 mmol, 1.0 equiv), KI (50 mol%), citric acid (10 mol%), and DPPE (20 mol%). MeCN (5 mL) with *n*-Bu_4_NClO_4_ (0.1 M) as an electrolyte was then added. The resulting solution was electrolyzed with a Pt minigrid electrode (52 mesh, 1 × 1.5 cm^2^) as anode and a Pt wire (diameter = 0.5 mm, height = 2.0 cm) as cathode, under a constant current (10 mA) in an undivided cell at room temperature for 4 h. After electrolysis, the mixture was quenched by water and extracted with ethyl acetate (3 × 10 mL). The combined organic layer was washed with brine (10 mL) and dried over Na_2_SO_4_. The ratio of **(*Z*)*-*2aa** and **(*E*)*-*2aa** was determined by ^19^F NMR (*Z/E* ratio = 19:1) of the crude mixture. ^19^F NMR (377 MHz, CDCl_3_) δ −115.97 (major), −116.73 (minor). The mixture of **(*Z*)*-*2aa** and **(*E*)*-*2aa** was obtained by a column chromatography separation of the crude mixture on silica gel (petroleum ether/ethyl acetate = 2:1), colorless oil, 100.0 mg, 96%. And, a further column chromatography separation could give the pure *Z*-isomer.

### Procedure for the scale-up synthesis of **2aa**

Into a round bottom flask was added KI (50 mol%), citric acid (10 mol%), and DPPE (20 mol%). MeCN (60 mL) with *n*-Bu_4_NClO_4_ (0.1 M) as an electrolyte was added. Thiol **1aa** (6 mmol, 1.0 equiv) was then introduced. The resulting solution was electrolyzed with a Pt minigrid electrode (52 mesh, 1 × 1.5 cm^2^) as anode and a Pt wire (diameter = 0.5 mm, height = 2.0 cm) as cathode, under a constant current (10 mA) in an undivided cell at room temperature. After 50 h, the mixture was quenched by water and extracted with ethyl acetate (3 × 30 mL). The combined organic layer was washed with brine (20 mL) and dried over Na_2_SO_4_, filtered, and concentrated in vacuo. The mixture of **(*Z*)*-*2aa** and **(*E*)*-*2aa** was obtained by a column chromatography separation of the crude mixture on silica gel (petroleum ether/ethyl acetate = 2:1), colorless oil, 0.97 g, 78%, *Z/E* ratio = 19:1.

## Supplementary information

Supplementary Information

Peer Review File

Description of Additional Supplementary Files

Supplementary Data 1

## Data Availability

The X-ray crystallographic coordinates for structures reported in this article have been deposited at the Cambridge Crystallographic Data Center (CCDC), under deposition number CCDC 1849256 (**(*****Z*****)*****-*****2ba**). The data can be obtained free of charge from The Cambridge Crystallographic Data Center via http://www.ccdc.cam.ac.uk/data_request/cif. For full characterization data including NMR spectra of new compounds and experimental details, see the Supplemental Information. Any further relevant data are available from the authors upon reasonable request.

## References

[1] Coates GW, Hustad PD, Reinartz S (2002). Catalysts for the living insertion polymerization of alkenes: access to new polyolefin architectures using Ziegler–Natta chemistry. Angew. Chem. Int. Ed..

[2] Fleming FF, Yao L, Ravikumar PC, Funk L, Shook BC (2010). Nitrile-containing pharmaceuticals: efficacious roles of the nitrile pharmacophore. J. Med. Chem..

[3] Fleming F (1999). Nitrile-containing natural products. Nat. Prod. Rep..

[4] Rappoport Z (1970). The Chemistry of the Cyano Group.

[5] Culkin DA, Hartwig JF (2003). Palladium-catalyzed α-arylation of carbonyl compounds and nitriles. Acc. Chem. Res..

[6] Bellina F, Rossi R (2010). Transition metal-catalyzed direct arylation of substrates with activated sp^3^-hybridized C−H bonds and some of their synthetic equivalents with aryl halides and pseudohalides. Chem. Rev..

[7] Johansson CCC, Colacot TJ (2010). Metal-catalyzed α-arylation of carbonyl and related molecules: novel trends in C−C bond formation by C−H bond functionalization. Angew. Chem. Int. Ed..

[8] Churchill D, Shin JH, Parkin G (1999). The Ansa effect in permethylmolybdenocene chemistry: a [Me2Si] Ansa bridge promotes intermolecular C−H and C−C bond activation. Organometallics.

[9] Evans ME, Li T, Jones WD (2009). Thermodynamic trends in carbon−hydrogen bond activation in nitriles and chloroalkanes at rhodium. J. Org. Chem..

[10] Wu L, Hartwig JF (2005). Mild palladium-catalyzed selective monoarylation of nitriles. J. Am. Chem. Soc..

[11] Kumagai N, Matsunaga S, Shibasaki M (2004). Cooperative catalysis of a cationic ruthenium complex, amine base, and Na salt: catalytic activation of acetonitrile as a nucleophile. J. Am. Chem. Soc..

[12] Wu T, Mu X, Liu G (2011). Palladium-catalyzed oxidative arylalkylation of activated alkenes: dual C−H bond cleavage of an arene and acetonitrile. Angew. Chem. Int. Ed..

[13] Kawato Y, Kumagai N, Shibasaki M (2013). Direct catalytic asymmetric addition of acetonitrile to *N*-thiophosphinoylimines. Chem. Commun..

[14] Shen J, Yang D, Liu R (2014). Copper-catalyzed aerobic oxidative coupling of aromatic alcohols and acetonitrile to β-ketonitriles. Org. Lett..

[15] Lang SB, Locascio TM, Tunge JA (2014). Activation of alcohols with carbon dioxide: intermolecular allylation of weakly acidic pronucleophiles. Org. Lett..

[16] Chu XQ, Shen ZL, Loh TP (2018). Recent advances in radical-initiated C(sp3)−H bond oxidative functionalization of alkyl nitriles. ACS Catal..

[17] Qiao K, Guo TF, Wan L, Guo K (2018). Iron(II)-catalyzed C-2 cyanomethylation of indoles and pyrroles via direct oxidative cross-dehydrogenative coupling with acetonitrile derivatives. Org. Chem. Front..

[18] Horn EJ, Rosen BR, Baran PS (2016). Synthetic organic electrochemistry: an enabling and innately sustainable method. ACS Cent. Sci..

[19] Jiao KJ, Zhao CQ, Mei TS (2017). Palladium catalyzed C–H functionalization with electrochemical oxidation. Tetrahedron Lett..

[20] Kakiuchi F, Kochi T (2017). Selective C−H functionalizations by electrochemical reactions with palladium catalysts. Isr. J. Chem..

[21] Cardoso DSP, Šljukić B, Sequeira CAC (2017). Organic electrosynthesis: from laboratorial practice to industrial applications. Org. Process Res. Dev..

[22] Yan M, Kawamata Y, Baran PS (2017). Synthetic organic electrochemical methods since 2000: on the verge of a renaissance. Chem. Rev..

[23] Jiang Y, Xu K, Zeng C (2018). Use of electrochemistry in the synthesis of heterocyclic structures. Chem. Rev..

[24] Pletcher D, Green RA, Brown RCD (2018). Flow electrolysis cells for the synthetic organic chemistry laboratory. Chem. Rev..

[25] Yoshida JI, Shimizu A, Hayashi R (2018). Electrogenerated cationic reactive intermediates: the pool method and further advances. Chem. Rev..

[26] Tang S, Liu Y, Lei A (2018). Electrochemical oxidative cross-coupling with hydrogen evolution: a green and sustainable way for bond formation. Chem.

[27] Wiebe A (2018). Electrifying organic synthesis. Angew. Chem. Int. Ed..

[28] Mçhle S (2018). Modern electrochemical aspects for the synthesis of value-added organic products. Angew. Chem. Int. Ed..

[29] Parry JB, Fu NK, Lin S (2018). Electrocatalytic difunctionalization of olefins as a general approach to the synthesis of vicinal diamines. Synlett.

[30] Sauer GS, Lin S (2018). An electrocatalytic approach to the radical difunctionalization of alkenes. ACS Catal..

[31] Moeller KD (2018). Using physical organic chemistry to shape the course of electrochemical reactions. Chem. Rev..

[32] Sauermann N, Meyer TH, Qiu YA, Ackermann L (2018). Electrocatalytic C−H activation. ACS Catal..

[33] Ma C, Fang P, Mei TS (2018). Recent advances in C-H functionalization using electrochemical transition metal catalysis. ACS Catal..

[34] Steckhan E (1986). Indirect electroorganic syntheses—a modern chapter of organic electrochemistry. Anew. Chem. Int. Ed. Engl..

[35] Ogibin YN, Elinson MN, Nikishin GI (2009). Mediator oxidation systems in organic electrosynthesis. Russ. Chem. Rev..

[36] Francke R, Little RD (2014). Redox catalysis in organic electrosynthesis: basic principles and recent developments. Chem. Soc. Rev..

[37] Nutting JE, Rafiee M, Stahl SS (2018). Tetramethylpiperidine N-oxyl (TEMPO), phthalimide N-oxyl (PINO), and related N-oxyl species: electrochemical properties and their use in electrocatalytic reactions. Chem. Rev..

[38] Liu K, Song CL, Lei A (2018). Recent advances in iodine mediated electrochemical oxidative cross-coupling. Org. Biomol. Chem..

[39] Wang HQ, Tan JJ, Zhang S, Xu K (2018). Electrosynthesis of trisubstituted 2-oxazolines via dehydrogenative cyclization of β-amino arylketones. Org. Lett..

[40] Zhang S, Xu K, Zeng CC (2018). Electrochemical formation of *N*-acyloxy amidyl radicals and their application: regioselective intramolecular amination of sp2 and sp3 C−H bonds. Org. Lett..

[41] Wu JW, Zhou Y, Lei A (2017). Electro-oxidative C(sp^3^)−H amination of azoles via intermolecular oxidative C(sp3)−H/N−H cross-coupling. ACS Catal..

[42] Xiong P, Xu HH, Xu HC (2018). Electrochemical difluoromethylarylation of alkynes. J. Am. Chem. Soc..

[43] Wu ZJ, Li SR, Xu HC (2018). Electrochemical dehydrogenative cyclization of 1,3-dicarbonyl compounds. Chem. Commun..

[44] Huang JM, Wang XX, Dong Y (2011). Electrochemical allylation reactions of simple imines in aqueous solution mediated by nanoscale zinc architectures. Angew. Chem., Int. Ed..

[45] Lai YL, Huang JM (2017). Palladium-catalyzed electrochemical allylic alkylation between alkyl and allylic halides in aqueous solution. Org. Lett..

[46] Lin DZ, Huang JM (2018). Electrochemical *N*-formylation of amines via decarboxylation of glyoxylic acid. Org. Lett..

[47] Du KS, Huang JM (2018). Electrochemical synthesis of bisindolylmethanes from indoles and ethers. Org. Lett..

[48] Baron H, Remfry FGP, Thorpe JF (1904). CLXXV—the formation and reactions of imino-compounds. Part I. Condensation of ethyl cyanoacetate with its sodium derivative. J. Chem. Soc. Trans..

[49] Ziegler K, Eberle H, Ohlinger H (1933). Über vielgliedrige Ringsysteme. I. Die präparativ ergiebige Synthese der Polymethylenketone mit mehr als 6 Ringgliedern. Justus Liebigs Ann. Chem. (in German).

[50] Schaefer, J. P.; Bloomfield, J. J. The Dieckmann Condensation (Including the Thorpe-Ziegler Condensation). *Org. React.* **1**, 1–203 (2011).

[51] Gardner JM, Abrahamsson M, Farnum BH, Meyer GJ (2009). Visible light generation of iodine atoms and I–I bonds: sensitized I^–^ oxidation and I_3_^–^ photodissociation. J. Am. Chem. Soc..

[52] Boschloo G, Hagfeldt A (2009). Characteristics of the iodide/triiodide redox mediator in dye-sensitized solar cells. Acc. Chem. Res..

[53] Liu D, Lei A (2015). Iodine-catalyzed oxidative coupling reactions utilizing C–H and X–H as nucleophiles. Chem. Asian J..

[54] Yang QL, Wang XY, Fang P, Mei TS (2018). Copper-catalyzed electrochemical C−H amination of arenes with secondary amines. J. Am. Chem. Soc..

[55] Qian P, Zha ZG, Wang ZY (2017). Electrocatalytic C−H/N−H coupling of 2′-aminoacetophenones for the synthesis of isatins. J. Org. Chem..

[56] Wang YK, Zha ZG, Wang ZY (2017). Efficient electrosynthesis of phosphinic amides via oxidative cross-coupling between N–H/P–H. Green Chem..

[57] Li YN, Zha ZG, Wang ZY (2016). Electrochemical synthesis of a-enaminones from aryl ketones. Chem. Commun..

[58] Xu K, Zha ZG, Wang ZY (2015). Electrosynthesis of enaminones directly from methyl ketones and amines with nitromethane as a carbon source. Chem. Commun..

[59] Conn JJ, Taurins A (1953). The structure and Grignard reaction of the β-aminocrotonitrile. Can. J. Chem..

[60] Ning YQ, Wu YB, Bi XH (2017). Radical enamination of vinyl azides: direct synthesis of N-unprotected enamines. Org. Lett..

[61] Yang HB, Selander N (2017). Divergent iron-catalyzed coupling of O-acyloximes with silyl enol ethers. Chem. Eur. J..

[62] Ke J, Tang YL, Lei A (2015). Copper-catalyzed radical/radical Csp^3^–H/P–H cross-coupling: α-phosphorylation of aryl ketone O-acetyloximes. Angew. Chem. Int. Ed..

[63] Ran LF, Ren ZH, Guan ZH (2014). Copper-catalyzed homocoupling of ketoxime carboxylates for synthesis of symmetrical pyrroles. Green Chem..

[64] Wang P, Tang S, Huang P, Lei A (2017). Electrocatalytic oxidant-free dehydrogenative C−H/S−H cross-coupling. Angew. Chem. Int. Ed..

[65] Huang PF, Wang P, Tang S, Lei A (2018). Electro-oxidative S−H/S−H cross-coupling with hydrogen evolution: facile access to unsymmetrical disulfides. Angew. Chem. Int. Ed..

[66] Mo ZY, Tang HT, Pan YM, Chen ZF (2018). Electrochemical sulfonylation of thiols with sulfonyl hydrazides: a metal- and oxidant-free protocol for the synthesis of thiosulfonates. Green Chem..

