# Supplementary material for: Stereoselective synthesis of sulfur-containing β-enaminonitrile derivatives through electrochemical Csp3–H bond oxidative functionalization of acetonitrile
Source: Nat Commun. 2019 Feb 19;10:833. doi: 10.1038/s41467-019-08762-5 (PMC6381189; doi:10.1038/s41467-019-08762-5)
Supplement: Supplementary file 1 — Supplementary Information [file 41467_2019_8762_MOESM1_ESM.pdf]

Supplementary Information

Stereoselective Synthesis of Sulfur-containing β-Enaminonitrile Derivatives through Electrochemical Csp^3–H Bond Oxidative Functionalization of Acetonitrile

He et al.

Supplementary Methods

Chemicals and Instrumentation

Commercial solvents and reagents were used without further purification unless otherwise noted.

Electrolysis reactions were conducted using a Model QJ3005T (32V) DC power supply purchased from Ningbo Jiuyuan Electronic Co., Ltd., China.

Analytical thin layer chromatography (TLC) plates and the silica gel (200 – 300 mesh) for column chromatography were phased from Qingdao Haiyang Chemical and Special Silica Gel Co, Ltd.

Proton nuclear magnetic resonance (^{1}H NMR) and carbon nuclear magnetic resonance (^{13}C NMR) spectroscopy were performed on Bruker Advance III-400 spectrometers (400 MHz for ^{1}H NMR, 100 MHz for ^{13}C NMR, 376 MHz for ^{19}F NMR). Chemical shifts of ^{1}H NMR, ^{13}C NMR and ^{19}F NMR spectra were reported as in units of parts per million (ppm) downfield from TMS (δ 0.0 ppm) and relative to the signal of $CDCl_3$ (δ 7.26 ppm for ^{1}H NMR and δ 77.1 ppm for ^{13}C NMR). Multiplicities were given as: s (singlet); br s (broad singlet); d (doublet); t (triplet); q (quartet); m (multiplets), etc. The number of protons (n) for a given resonance was indicated by nH.

GC-MS analyses were obtained on a Thermo Scientific Trace GC Ultra Gas Chromatograph equipped with a TG-5MS 30 m × 0.25 mm ID × 0.25 µm capillary column (Thermo Scientific). The GC was directly interfaced to a Thermo Scientific DSQ single quadrupole mass spectrometer (EI, 70 eV). The following GC oven temperature programs were used: 80 °C hold for 2 min, ramp 25 °C min^{-1} to a final temperature of 280 °C, and hold for 3 min. Helium was used as a carrier gas, with a constant column flow of 1.0 mL min^{-1}. The injector temperature was held constant at 260 °C; Melting points were measured on an Electrothermal SGW-X4 microscopy digital melting point apparatus and are uncorrected; IR spectra were recorded on a FTLA2000 spectrometer; HR-MS was carried out on a high-resolution mass spectrometer (LCMS-IT-TOF). Cyclic voltammetry (CV) analysis was performed on Ingsens IGS-1030 electrochemical workstation (Ingsens Instruments (Guangzhou) Co., Ltd., China) with a conventional three-electrode cell, using a platinum electrode (d = 2 mm) as working electrode, a Pt wire as counter electrode and saturated calomel electrode (SCE) as a reference electrode. Cyclic voltammograms were recorded at 100 mV s^{-1} scan rate.

Additional Results of Optimization Study

Supplementary Table 1 Screening of acids and bases *a*

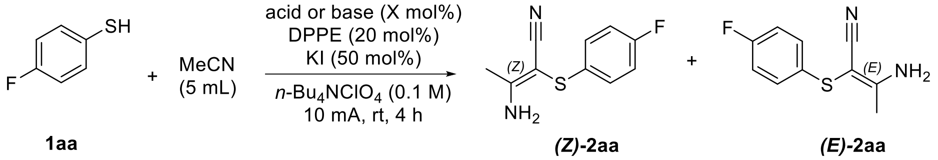

entry	acid or base	loading (mol%)	yield(%)[c]	*Z/E*[c]
1	triethylamine	10	0	
2	*N,N*-diisopropyl-ethylamine	10	0	
3	*t*-BuOK	10	0	
4	citric acid	10	96	19:1
5	acetic acid	10	79	13:1
6	oxalic acid	10	72	12:1
7	phosphoric acid	10	70	11:1
8	1,1'-binaphthyl-2,2'-diyl hydrogenphosphate[b]	10	84	10:1
9	amberlyst-15(H)	10	75	12:1
10	trifluoroacetic acid	10	40	9:1
11	citric acid	5	93	11:1
12	citric acid	15	98	13:1
13	citric acid	20	83	9:1

[a] Standard conditions: **1a** (0.5 mmol), 20 mol % DPPE, 50 mol % KI, MeCN (5 mL), with 0.1 M n-Bu_4NClO_4 as electrolyte. A Pt minigrid electrode (52 mesh, 1 × 1.5 cm^2) as anode and a Pt wire (diameter = 0.5 mm, height = 2.0 cm) as cathode, an undivided cell, constant current = 10 mA, 4 h, room temperature. [b] Without DPPE. [c] Yields and *Z/E* ratios were determined by ^{19}F NMR analysis of the crude reaction mixture using fluorobenzene as the internal standard.

Supplementary Table 2 Screening of redox catalysts *a*

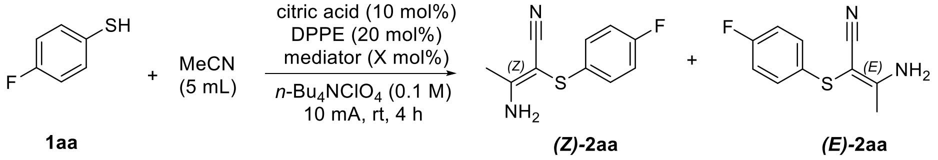

entry	Mediator	loading (mol%)	yield(%)[b]	*Z/E*[b]
1	KI	50	96	19:1
2	NaI	50	95	11:1
3	KBr	50	0	
4	TBAI	50	60	12:1
5	NH_4I	50	96	12:1
6	KI	0	0	
7	KI	25	87	11:1
8	KI	75	97	13:1

[a] Standard conditions: **1a** (0.5 mmol), 10 mol % citric acid, 50 mol % KI, MeCN (5 mL), with 0.1 M n-Bu_4NClO_4 as electrolyte. A Pt minigrid electrode (52 mesh, 1 × 1.5 cm^2) as anode and a Pt wire (diameter = 0.5 mm, height = 2.0 cm) as cathode, an undivided cell, constant current = 10 mA, 4 h, room temperature. [b] Yields and *Z/E* ratios were determined by ^{19}F NMR analysis of the crude reaction mixture using fluorobenzene as the internal standard.

Supplementary Table 3 Screening of ligands [a]

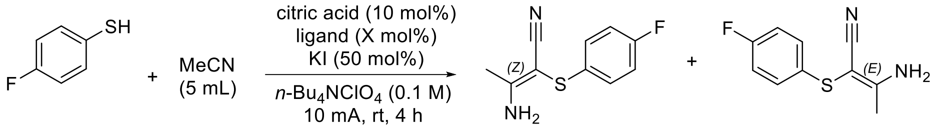

entry	ligand	loading (mol%)	yield(%)[b]	*Z/E*[b]
1	1,10-phenanthroline	20	0	
2	2,2'-bipyridine	20	0	
3	triphenylphosphine	20	70	10:1
4	trianisylphosphine	20	45	7:1
5	DPEphos	20	70	8:1
6	XANTphos	20	71	11:1
7	diphenylphosphine	20	75	12:1
8	DPPM	20	85	10:1
9	DPPE	20	96	19:1
10	DPPP	20	86	10:1
11	DPPE	10	95	10:1
12	DPPE	30	90	12:1

[a] Standard conditions: **1a** (0.5 mmol), 10 mol % citric acid, MeCN (5 mL), with 0.1 M n-Bu_4NClO_4 as electrolyte. A Pt minigrid electrode (52 mesh, 1 × 1.5 cm^2) as anode and a Pt wire (diameter = 0.5 mm, height = 2.0 cm) as cathode, an undivided cell, constant current = 10 mA, 4 h, room temperature. [b] Yields and *Z/E* ratios were determined by ^{19}F NMR analysis of the crude reaction mixture using fluorobenzene as the internal standard.

Supplementary Table 4 Screening of electrolytes *a*

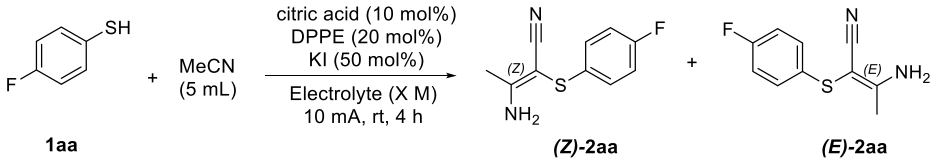

entry	Electrolyte	loading (M)	yield(%)[b]	*Z/E*[b]
1	*n*-Bu_4NClO_4	0.1	96	19:1
2	*n*-Bu_4NBF_4	0.1	76	9:1
3	*n*-Bu_4NPF_6	0.1	95	11:1
4	$LiClO_4$	0.1	0	
5	$NaClO_4$	0.1	0	
6	*n*-Bu_4NClO_4	0	56	11:1
7	*n*-Bu_4NClO_4	0.05	86	9:1
8	*n*-Bu_4NClO_4	0.2	82	10:1

[a] Standard conditions: **1a** (0.5 mmol), 10 mol % citric acid, 50 mol % KI, MeCN (5 mL), with X M Electrolyte as electrolyte. A Pt minigrid electrode (52 mesh, 1 × 1.5 cm^2) as anode and a Pt wire (diameter = 0.5 mm, height = 2.0 cm) as cathode, an undivided cell, constant current = 10 mA, 4 h, room temperature. [b] Yields and *Z/E* ratios were determined by ^{19}F NMR analysis of the crude reaction mixture using fluorobenzene as the internal standard.

Supplementary Table 5 Screening of electrode materials *a*

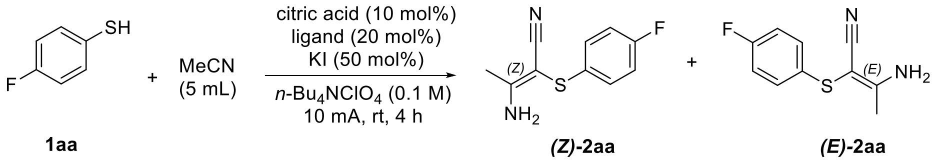

entry	Variation from the standard conditions	yield(%)[b]	Z/E[b]
1	none	96	19:1
2	RVC as an anode[c]	95	14:1
3	Carbon plate as an anode[c]	95	12:1
4	Carbon rod as an anode[c]	94	14:1
5	Carbon cloth as an anode[c]	91	12:1
6	Carbon cloth as a cathode[c]	35	11:1

[a] Standard conditions: **1aa** (0.5 mmol), citric acid (10 mol %), DPPE (20 mol %), KI (50 mol %), MeCN (5 mL), with 0.1 M *n*-Bu_4NClO_4 as electrolyte. A Pt minigrid electrode (52 mesh, 1 × 1.5 cm^2) as an anode and a Pt wire (diameter = 0.5 mm, height = 2.0 cm) as a cathode, an undivided cell, constant current = 10 mA, 4 h, room temperature. [b] Yields and *Z/E* ratios were determined by ^{19}F NMR analysis of the crude reaction mixture using fluorobenzene as the internal standard. [c] RVC anode (100 PPI, 1.0 x 1.5 x 1.0 cm^3), Carbon rod anode (diameter: 0.5 cm, height: 1.78 cm), Carbon cloth anode (1.0 × 1.5 cm^2), Carbon cloth cathode (0.3 × 1.5 cm^2).

General procedure for the synthesis of products

Into a round bottom flask, thiol **1** (0.5 mmol, 1.0 equiv), KI (50 mol %), citric acid (10 mol %) and DPPE (20 mol %) was added. MeCN (5 mL) with *n*-Bu_4NClO_4 (0.1 M) as an electrolyte was then added. The resulting solution was electrolyzed with a Pt minigrid electrode (52 mesh, 1 × 1.5 cm^2) as anode and a Pt wire (diameter = 0.5 mm, height = 2.0 cm) as cathode, under a constant current (10 mA) in an undivided cell at room temperature for 4 hours. After electrolysis, the mixture was quenched by water and extracted with ethyl acetate (3 × 10 mL). The combined organic layer was washed with brine (10 mL) and dried over Na_2SO_4. Purified product **2** was obtained after column chromatography on silica gel using a solvent mixture of petroleum ether and ethyl acetate.

Procedure for Scale-up synthesis and product transformations

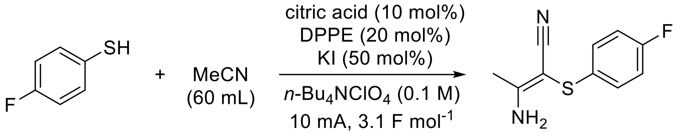

1aa, 6 mmol   **2aa**, 0.97 g, 78%, *Z/E* 19:1

Supplementary Equation 1

Into a round bottom flask, KI (50 mol %), citric acid (10 mol %) and DPPE (20 mol %) was added. MeCN (60 mL) with *n*-Bu_4NClO_4 (0.1 M) as an electrolyte was added. Thiol **1aa** (6 mmol, 1.0 equiv) was then introduced. The resulting solution was electrolyzed with a Pt minigrid electrode (52 mesh, 1 × 1.5 cm^2) as anode and a Pt wire (diameter = 0.5 mm, height = 2.0 cm) as cathode, under a constant current (10 mA) in an undivided cell at room temperature. After 50 hours, the mixture was quenched by water and extracted with ethyl acetate (3 × 30 mL). The combined organic layer was washed with brine (20 mL) and dried over Na_2SO_4, filtered and concentrated in *vacuo*. The mixture of ***(Z)*-2aa** and ***(E)*-2aa** was obtained by a column chromatography separation of the crude mixture on silica gel (petroleum ether/ethyl acetate = 2:1), colorless oil, 0.97 g, 78%, *Z/E* ratio = 19:1.

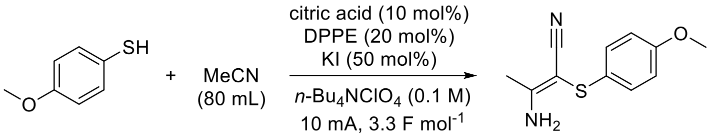

1ah, 8 mmol   **2ah**, 1.43 g, 81%, *Z/E* 10:1

Supplementary Equation 2

Into a round bottom flask, KI (50 mol %), citric acid (10 mol %) and DPPE (20 mol %) was added. MeCN (80 mL) with *n*-Bu_4NClO_4 (0.1 M) as an electrolyte was added. Thiol **1ah** (8 mmol, 1.0 equiv) was then introduced. The resulting solution was electrolyzed with a Pt minigrid electrode (52 mesh, 1 × 1.5 cm^2) as anode and a Pt wire (diameter = 0.5 mm, height = 2.0 cm) as cathode, under a constant current (10 mA) in an undivided cell at room temperature. After 70 hours, the mixture was quenched by water and extracted with ethyl acetate (3 × 35 mL). The combined organic layer was washed with brine (25 mL) and dried over Na_2SO_4, filtered and

concentrated in *vacuo*. The mixture of ***(Z)*-2ah** and ***(E)*-2ah** was obtained by a column chromatography separation of the crude mixture on silica gel (petroleum ether/ethyl acetate = 3:2), colorless oil, 1.43 g, 81%, *Z/E* ratio = 10:1.

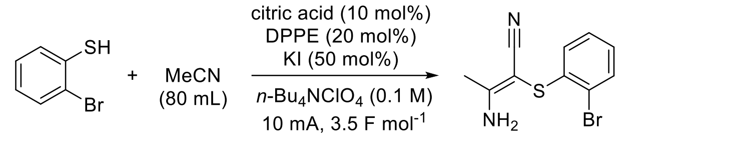

1bb, 8 mmol **2bb**, 1.39 g, 65%, *Z/E* 17:1

Supplementary Equation 3

Into a round bottom flask, KI (50 mol %), citric acid (10 mol %) and DPPE (20 mol %) was added. MeCN (80 mL) with *n*-Bu_4NClO_4 (0.1 M) as an electrolyte was added. Thiol **1bb** (8 mmol, 1.0 equiv) was then introduced. The resulting solution was electrolyzed with a Pt minigrid electrode (52 mesh, 1 × 1.5 cm^2) as anode and a Pt wire (diameter = 0.5 mm, height = 2.0 cm) as cathode, under a constant current (10 mA) in an undivided cell at room temperature. After 75 hours, the mixture was quenched by water and extracted with ethyl acetate (3 × 35 mL). The combined organic layer was washed with brine (25 mL) and dried over Na_2SO_4, filtered and concentrated in *vacuo*. The mixture of ***(Z)*-2bb** and ***(E)*-2bb** was obtained by a column chromatography separation of the crude mixture on silica gel (petroleum ether/ethyl acetate = 3:2), colorless oil, 1.39 g, 65%, *Z/E* ratio = 17:1.

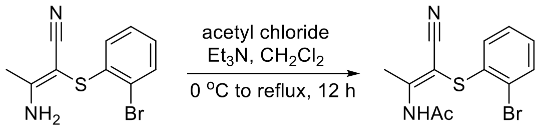

2bb **3a**, 85%

Supplementary Equation 4

***(Z)*-*N*-(1-((2-bromophenyl)thio)-1-cyanoprop-1-en-2-yl)acetamide (3a)**.[1] **2bb** (134.5 mg, 0.50 mmol) was dissolved in 2.5 mL of CH_2Cl_2 and cooled to 0 °C. Triethylamine (98 µL, 0.65 mmol) was added to the **2bb** solution followed by the corresponding acid chloride (43 µL, 0.60 mmol) dropwisely. Then the mixture was stirred under reflux for 12 h. The mixture was quenched by saturated $NaHCO_3$ (aq) and extracted with CH_2Cl_2 (3 × 10 mL). The combined organic layer was washed with brine (10 mL) and dried over Na_2SO_4. Evaporation and column chromatography on silica gel afforded **3a** as colorless oil (132.3 mg, 85%).

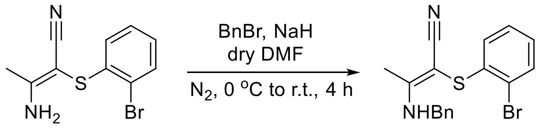

2bb **3b**, 79%

Supplementary Equation 5

***(Z)*-3-(benzylamino)-2-((2-bromophenyl)thio)but-2-enenitrile (3b)**.[2] Sodium hydride (60% in oil, 7.2 mg, 0.18 mmol) was suspended under nitrogen in dry DMF (0.7 mL) and cooled to 0 °C.

2bb (37.7 mg, 0.14 mmol) in dry DMF (0.7 mL) was added, and stirring in ice was continued for 15 min. Benzyl bromide (20 µL, 0.17 mmol) was added and the reaction mixture was allowed to warm slowly to room temperature with the control of the exotherm and the gas evolution. Stirring at 25 °C was continued for 4 h. The mixture was quenched by saturated NH_4Cl (aq) and extracted with ethyl acetate (3 × 5 mL). The combined organic layer was washed with brine (5 mL) and dried over Na_2SO_4. Evaporation and column chromatography on silica gel afforded **3b** as colorless oil (39.7 mg, 79%).

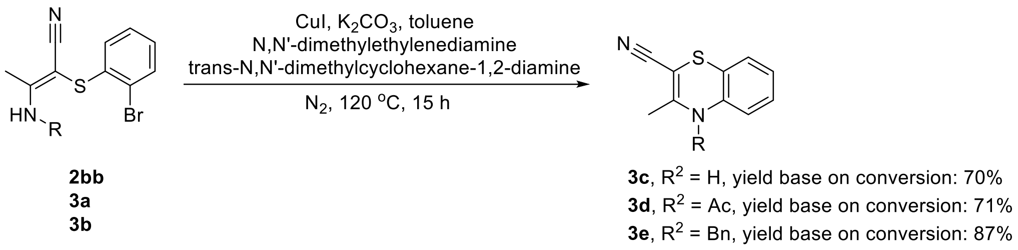

Supplementary Equation 6

Procedure for the synthesis of 4H-1,4-benzothiazine derivatives.[3,4] An oven-dried screw-cap test tube was charged with CuI (20 mol %.), **2bb/3a/3b** (1.0 e.q.), K_2CO_3 (2.0 e.q.), and a magnetic stir bar. The test tube was evacuated and back-filled with dry nitrogen. *N,N'*-dimethylethylenediamine (40 mol%), *trans-N,N'*-dimethylcyclohexane-1,2-diamine (40 mol%) and toluene (0.1 M) were then added. The vessel was immersed in a pre-heated oil bath and stirred vigorously at 120 °C for 15 h. The reaction mixture was cooled to room temperature, extracted with ethyl acetate. The combined organic layer was washed with brine and dried over Na_2SO_4. The filtrate was concentrated and the resulting residue was purified by flash chromatography (petroleum ether/ethyl acetate) to provide the desired product **3c**/**3d**/**3e**.

Supplementary Discussion

Monitoring of the pH during the reaction process

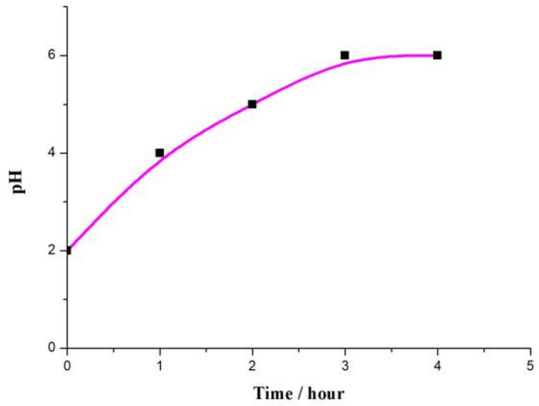

Supplementary Figure 1 Monitoring of the pH during the reaction process

Studies on the role of DPPE

It was observed that 1,2-ethylene bis(diphenylphosphine oxide) was collected after the reaction. In order to study if the oxide had played a role on the stereoselective reaction, a control reaction was carried out. The reaction was run in the absence of KI until DPPE was fully oxidized to 1,2-ethylene bis(diphenylphosphine oxide). KI was then added and the reaction furnished with a same yield and *Z/E* ratio as the standard one. This result demonstrated that the corresponding oxide of DPPE might have worked in the reaction to increase the stereoselectivity of the olefin.

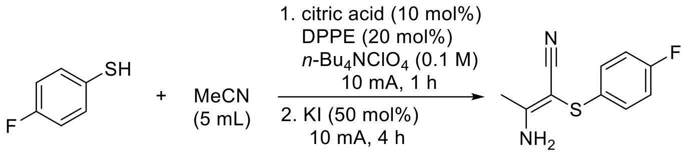

Supplementary Equation 7

Experimental procedure: Into a round bottom flask was added thiol **1aa** (0.5 mmol, 1.0 equiv), citric acid (10 mol %) and DPPE (20 mol %). MeCN (5 mL) with *n*-Bu_4NClO_4 (0.1 M) as an electrolyte was then added. The resulting solution was electrolyzed with a Pt minigrid electrode (52 mesh, 1 × 1.5 cm^2) as anode and a Pt wire (diameter = 0.5 mm, height = 2.0 cm) as cathode, under a constant current (10 mA) in an undivided cell at room temperature. After 1 h, ^{31}P NMR analysis showed that DPPE was completely converted to its corresponding oxide. Then, KI (50 mol %) was added into the round bottom flask and the reaction continued. After electrolysis, the mixture was quenched by water and extracted with ethyl acetate (3 × 10 mL). The combined organic layer was washed with brine (10 mL) and dried over Na_2SO_4, filtered and concentrated in *vacuo*. The crude product was obtained as a mixture of ***(Z)*-2aa** and ***(E)*-2aa** (colorless oil, *Z/E* ratio = 19:1, 92%).

DFT studies of the mechanism

Computational Details

All calculations were performed using Gaussian 09 D.01. program.[5] Geometry optimizations were carried out at the M06-2X[6]/6-311++g (d, p) for all atoms. All structures were fully optimized accounting for acetonitrile solvation effects using the SMD continuum solvent model.[7] Frequency analysis calculations were performed to characterize the structures to be the minima (no imaginary frequency). For reactions in solution, standard free energies refer to a 1 M standard-state concentration for all species. Compared to the conventional 1 atm standard state for gas-phase calculations, this requires the introduction of a concentration-change term of RTln(24.5), which is equal to 1.9 kcal mol^{-1} at 298 K.[8] Based on this method, a correction of 1.9 kcal mol^{-1} is applied when a component of the reaction changes (i.e., a reaction from m components to n components, the correction is (n-m) × 1.9 kcal mol^{-1}). And in order to reduce the overestimation of entropy, a scaling factor of 0.5 to the gas-phase entropic contributions (i.e., −TΔS) was applied.[9,10] The ratio of ***(Z)*-2aa** and ***(E)*-2aa** was calculated by Supplementary Equation 8.

$$\frac{[(\boldsymbol{Z})-\boldsymbol{2aa}]}{[(\boldsymbol{E})-\boldsymbol{2aa}]} = \mathrm{e}^{\left(\frac{4185.85\times\left(G_{(\boldsymbol{E})}-G_{(\boldsymbol{Z})}\right)/(\mathrm{kcal\cdot mol^{-1}})}{1.38\times10^{-23}\times298.15\times6.02\times10^{23}}\right)}$$

Supplementary Equation 8

Where the $G_{(E)}$-$G_{(Z)}$= ΔG= 1.9kcal mol^{-1}. All of the thermodynamic data were obtained at 298.15 K, and additional computational information and the Cartesian coordinates of the optimized structure are given in the Supplementary Data File.

Cyclic voltammograms

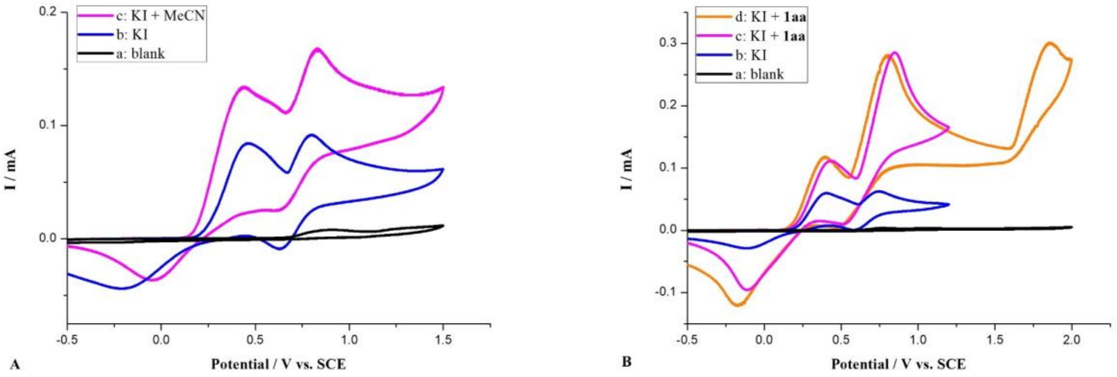

Supplementary Figure 2 A. Cyclic voltammograms of 0.1 M *n*-Bu_4NClO_4 solution in DMF at room temperature. (a) None; (b) KI (50 mmol L^{-1}); (c) KI (50 mmol L^{-1}) + MeCN (1 mL). B. Cyclic voltammograms of 0.1 M *n*-Bu_4NClO_4 solution in MeCN at room temperature. (a) None; (b) KI (50 mmol L^{-1}); (c) KI (50 mmol L^{-1}) + **1aa** (2 mmol L^{-1}), potential range from –0.5 to 1.2 V; (d) KI (50 mmol L^{-1}) + **1aa** (2 mmol L^{-1}), potential range from –0.5 to 2.0 V. The voltammogram was obtained with Pt wire as an auxiliary electrode and a saturated calomel electrode (SCE) as a reference electrode. The scan rate was 0.1 V/s on a platinum disk electrode (d = 2 mm).

Cyclic voltammetry studies on the mixture of **1aa**, acetonitrile, and KI (Supplementary Figure 2, curve c) also exhibited obvious catalytic currents compared with only KI in the acetonitrile solution (Supplementary Figure 2, curve b). In addition, the oxidation peak of **1aa** was observed at 1.85 V vs SCE (Supplementary Figure 2, curve d).

Detail descriptions for products

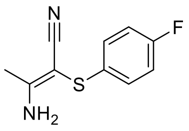

***(Z)*-2aa**

***(Z)*-3-Amino-2-((4-Fluorophenyl)thio)-crotononitrile (*(Z)*-2aa).** The ratio of ***(Z)*-2aa** and ***(E)*-2aa** was determined by ^{19}F NMR (*Z/E* ratio = 19:1) of the crude mixture. ^{19}F NMR (377 MHz, $CDCl_3$) δ -115.97 (major), -116.73 (minor). The mixture of ***(Z)*-2aa** and ***(E)*-2aa** was obtained by a column chromatography separation of the crude mixture on silica gel (petroleum ether/ethyl acetate = 2:1), colorless oil, 100.0 mg, 96%.

The *Z*-isomer was obtained by a further separation of the mixture of ***(Z)*-2aa** and ***(E)*-2aa** with the column chromatography on silica gel (petroleum ether/ethyl acetate = 2:1). ^{1}H NMR (400 MHz, $CDCl_3$): δ 7.24 – 7.19 (m, 2H), 7.00 – 6.96 (m, 2H), 5.66 (br s, 2H), 2.27 (s, 3H). ^{13}C NMR (100 MHz, $CDCl_3$): δ 164.9, 161.7 (d, J_{C-F} = 246.0 Hz), 130.4 (d, J_{C-F} = 3.2 Hz), 128.5 (d, J_{C-F} = 8.0 Hz), 121.3, 116.3 (d, J_{C-F} = 22.1 Hz), 66.6, 20.1. IR (KBr, cm^{-1}): ν = 3430, 2182, 1632, 1550, 1490, 1418. HRMS (m/z) calcd. for $C_{10}H_9FN_2NaS$ $[M+Na]^+$: 231.0368; found: 231.0363.

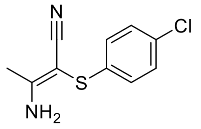

***(Z)*-2ab**

***(Z)*-3-Amino-2-((4-Chlorophenyl)thio)-crotononitrile (*(Z)*-2ab).** The mixture of ***(Z)*-2ab** and ***(E)*-2ab** was obtained by a separation of the crude mixture and the ratio of **2ab** was determined by a ^{1}H NMR analysis (colorless oil, *Z/E* ratio = 9:1, 106.7 mg, 95%).

The *Z*-isomer was obtained by a further separation of the mixture of ***(Z)*-2ab** and ***(E)*-2ab** with the column chromatography on silica gel (petroleum ether/ethyl acetate = 2:1). ^{1}H NMR (400 MHz, $CDCl_3$): δ 7.26 (d, J = 7.8 Hz, 2H), 7.16 (d, J = 8.5 Hz, 2H), 2.32 (s, 3H). ^{13}C NMR (100 MHz, $CDCl_3$): δ 164.8, 133.8, 132.1, 129.3, 127.5, 120.8, 66.4, 20.3. IR (KBr, cm^{-1}): ν = 3437, 3416, 2188, 1630, 1545, 1475, 1390. HRMS (m/z) calcd. for $C_{10}H_9ClN_2NaS$ $[M+Na]^+$: 247.0073; found: 247.0067.

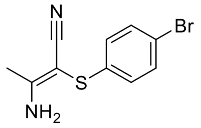

***(Z)*-2ac**

***(Z)*-3-Amino-2-((4-Bromophenyl)thio)-crotononitrile (*(Z)*-2ac).** The mixture of ***(Z)*-2ac** and ***(E)*-2ac** was obtained by a separation of the crude mixture and the ratio of ***(Z)*-2ac** and ***(E)*-2ac** was determined by a ^{1}H NMR analysis (colorless oil, *Z/E* ratio = 9:1, 122.1 mg, 90%).

The *Z*-isomer was obtained by a further separation of the mixture of ***(Z)*-2ac** and ***(E)*-2ac** with the column chromatography on silica gel (petroleum ether/ethyl acetate = 2:1). ^{1}H NMR (400 MHz, $CDCl_3$): δ 7.40 (d, J = 8.6 Hz, 2H), 7.10 (d, J = 8.6 Hz, 2H), 2.32 (s, 3H). ^{13}C NMR (100 MHz, $CDCl_3$): δ 165.1, 134.6, 132.2, 127.7, 120.9, 119.8, 65.7, 20.3. IR (KBr, cm^{-1}): ν = 3423, 2176, 1624, 1547, 1471. HRMS (m/z) calcd. for $C_{10}H_9BrN_2NaS$ $[M+Na]^+$: 290.9568; found: 290.9562.

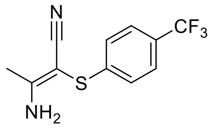

***(Z)*-2ad**

***(Z)*-3-Amino-2-((4-(Trifluoromethyl)phenyl)thio)-crotononitrile (*(Z)*-2ad).** The mixture of ***(Z)*-2ad** and ***(E)*-2ad** was obtained by a separation of the crude mixture and the ratio of **2ad** was determined by a ^{1}H NMR analysis (colorless oil, *Z/E* ratio = 9:1, 100.7 mg, 78%).

The *Z*-isomer was obtained by a further separation of the mixture of ***(Z)*-2ad** and ***(E)*-2ad** with the column chromatography on silica gel (petroleum ether/ethyl acetate = 2:1). ^{1}H NMR (400 MHz, $CDCl_3$): δ 7.53 (d, J = 8.2 Hz, 2H), 7.30 (d, J = 8.2 Hz, 2H), 2.36 (s, 3H). ^{13}C NMR (100 MHz, $CDCl_3$): δ 165.4, 140.4, 128.1 (q, J_{C-F} = 33.0 Hz), 126.0 (q, J_{C-F} = 3.0 Hz), 125.5, 123.8 (d, J_{C-F} = 223.0 Hz), 120.5, 64.8, 20.4. ^{19}F NMR (377 MHz, $CDCl_3$) δ -62.38. IR (KBr, cm^{-1}): ν = 3441, 2186, 1626, 1543, 1403, 1330. HRMS (m/z) calcd. for $C_{11}H_9F_3N_2NaS$ $[M+Na]^+$: 281.0336; found: 281.0331.

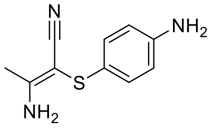

***(Z)*-2ae**

***(Z)*-3-Amino-2-((4-(Aminomethyl)phenyl)thio)-crotononitrile (*(Z)*-2ae).** The mixture of ***(Z)*-2ae** and ***(E)*-2ae** was obtained by a separation of the crude mixture and the ratio of ***(Z)*-2ae** and ***(E)*-2ae** was determined by a ^{1}H NMR analysis (colorless oil, *Z/E* ratio = 7:1, 24.6 mg, 24%).

The *Z*-isomer was obtained by a further separation of the mixture of ***(Z)*-2ae** and ***(E)*-2ae** with the column chromatography on silica gel (petroleum ether/ethyl acetate = 1:1). ^{1}H NMR (400 MHz, DMSO-d^6): δ 7.42 (br s, 1H), 7.14 (br s, 1H), 7.01 (d, J = 7.6 Hz, 2H), 6.53 (d, J = 8.0 Hz, 2H), 5.15 (br s, 2H), 2.09 (s, 3H). ^{13}C NMR (100 MHz, DMSO-d^6): δ 165.3, 148.5, 130.6, 123.2, 121.1, 115.0, 65.1, 20.1. IR (KBr, cm^{-1}): ν = 3378, 2173, 1642, 1552, 1495, 1417. HRMS (m/z) calcd. for $C_{10}H_{12}N_3S$ $[M+H]^+$: 206.0752; found: 206.0746.

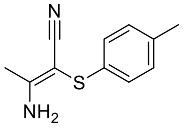

***(Z)*-2af**

***(Z)*-3-Amino-2-((4-Methylphenyl)thio)-crotononitrile (*(Z)*-2af).** The mixture of ***(Z)*-2af** and ***(E)*-2af** was obtained by a separation of the crude mixture and the ratio of ***(Z)*-2af** and ***(E)*-2af** was determined by a ^{1}H NMR analysis (colorless oil, *Z/E* ratio = 11:1, 94.0 mg, 92%).

The *Z*-isomer was obtained by a further separation of the mixture of ***(Z)*-2af** and ***(E)*-2af** with the column chromatography on silica gel (petroleum ether/ethyl acetate = 7:4). ^{1}H NMR (400 MHz, $CDCl_3$): δ 7.14 (d, J = 8.2 Hz, 2H), 7.09 (d, J = 8.0 Hz, 2H), 2.30 (s, 3H), 2.27 (s, 3H). ^{13}C NMR (100 MHz, $CDCl_3$): δ 164.4, 136.2, 131.7, 130.0, 126.8, 121.4, 67.0, 21.0, 20.2. IR (KBr, cm^{-1}): ν = 3424, 2174, 1622, 1547, 1493, 1419. HRMS (m/z) calcd. for $C_{11}H_{12}N_2NaS$ $[M+Na]^+$: 227.0619; found: 227.0613.

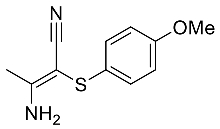

***(Z)*-2ag**

***(Z)*-3-Amino-2-((4-Methoxyphenyl)thio)-crotononitrile (*(Z)*-2ag).** The mixture of ***(Z)*-2ag** and ***(E)*-2ag** was obtained by a separation of the crude mixture and the ratio of ***(Z)*-2ag** and ***(E)*-2ag** was determined by a ^{1}H NMR analysis (colorless oil, *Z/E* ratio = 10:1, 105.7 mg, 96%).
The *Z*-isomer was obtained by a further separation of the mixture of ***(Z)*-2ag** and ***(E)*-2ag** with the column chromatography on silica gel (petroleum ether/ethyl acetate = 3:2). ^{1}H NMR (400 MHz, $CDCl_3$): δ 7.21 (d, J = 8.4 Hz, 2H), 6.82 (d, J = 8.4 Hz, 2H), 3.76 (s, 3H), 2.22 (s, 3H). ^{13}C NMR (100 MHz, $CDCl_3$): δ 164.4, 158.8, 129.3, 126.1, 121.8, 114.9, 67.7, 55.5, 20.1. IR (KBr, cm^{-1}): v = 3441, 2181, 1623, 1555, 1493. HRMS (m/z) calcd. for $C_{11}H_{12}N_2NaOS$ $[M+Na]^+$: 243.0568; found: 243.0563.

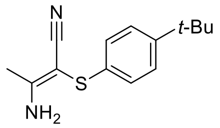

***(Z)*-2ah**

***(Z)*-3-Amino-2-((4-*t*-Butylphenyl)thio)-crotononitrile (*(Z)*-2ah).** The mixture of ***(Z)*-2ah** and ***(E)*-2ah** was obtained by a separation of the crude mixture and the ratio of ***(Z)*-2ah** and ***(E)*-2ah** was determined by a ^{1}H NMR analysis (colorless oil, *Z/E* ratio = 9:1, 93.6 mg, 76%).
The *Z*-isomer was obtained by a further separation of the mixture of ***(Z)*-2ah** and ***(E)*-2ah** with the column chromatography on silica gel (petroleum ether/ethyl acetate = 7:4). ^{1}H NMR (400 MHz, $CDCl_3$): δ 7.31 (d, J = 8.3 Hz, 2H), 7.17 (d, J = 8.3 Hz, 2H), 2.28 (s, 3H), 1.29 (s, 9H). ^{13}C NMR (100 MHz, $CDCl_3$): δ 164.6, 149.5, 131.8, 126.3, 121.4, 66.7, 34.5, 31.3, 20.2. IR (KBr, cm^{-1}): v = 3432, 2179, 1620, 1548, 1490, 1397. HRMS (m/z) calcd. for $C_{14}H_{18}N_2NaS$ $[M+Na]^+$: 269.1088; found: 269.1083.

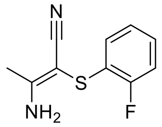

***(Z)*-2ba**

***(Z)*-3-Amino-2-((2-Fluorophenyl)thio)-crotononitrile (*(Z)*-2ba).** The ratio of ***(Z)*-2ba** and ***(E)*-2ba** was determined by ^{19}F NMR (*Z/E* ratio = 16:1) of the crude mixture. ^{19}F NMR (377 MHz, $CDCl_3$) δ -111.67 (major), -112.77 (minor). The mixture of ***(Z)*-2ba** and ***(E)*-2ba** was obtained by a column chromatography separation of the crude mixture on silica gel (petroleum ether/ethyl acetate = 2:1), colorless oil, 100.0 mg, 96%.
The *Z*-isomer was obtained by a further separation of the mixture of ***(Z)*-2ba** and ***(E)*-2ba** with the column chromatography on silica gel (petroleum ether/ethyl acetate = 2:1). mp: 118 – 120°C. ^{1}H NMR (400 MHz, $CDCl_3$): δ 7.32 – 7.29 (m, 1H), 7.23 – 7.18 (m, 1H), 7.12 – 7.03 (m, 2H), 2.29 (s, 3H). ^{13}C NMR (100 MHz, $CDCl_3$): δ 165.4, 160.0 (d, $J_{C\text{-}F}$ = 243.0 Hz), 129.2 (d, $J_{C\text{-}F}$ = 2.0 Hz), 128.3 (d, $J_{C\text{-}F}$ = 8.0 Hz), 124.8 (d, $J_{C\text{-}F}$ = 3.0 Hz), 122.3 (d, $J_{C\text{-}F}$ = 18.0 Hz), 121.2, 115.8

(d, J_{C-F} = 22.0 Hz), 64.3, 20.2. IR (KBr, cm^{-1}): ν = 3415, 2178, 1635, 1547, 1469. HRMS (m/z) calcd. for $C_{10}H_9FN_2NaS$ $[M+Na]^+$: 231.0368; found: 231.0363.

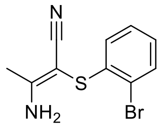

***(Z)*-2bb**

***(Z)*-3-Amino-2-((2-Bromophenyl)thio)-crotononitrile (*(Z)*-2bb).** The mixture of ***(Z)*-2bb** and ***(E)*-2bb** was obtained by a separation of the crude mixture and the ratio of ***(Z)*-2bb** and ***(E)*-2bb** was determined by a ^1H NMR analysis (colorless oil, *Z/E* ratio = 18:1, 102.3 mg, 76%).
The *Z*-isomer was obtained by a further separation of the mixture of ***(Z)*-2bb** and ***(E)*-2bb** with the column chromatography on silica gel (petroleum ether/ethyl acetate = 2:1). ^1H NMR (400 MHz, $CDCl_3$): δ 7.51 (d, J = 7.9 Hz, 1H), 7.27 (d, J = 6.2 Hz, 1H), 7.16 (d, J = 8.0 Hz, 1H), 7.04 (dd, J = 7.6, 7.8 Hz, 1H), 2.36 (s, 3H). ^{13}C NMR (100 MHz, $CDCl_3$): δ 165.7, 136.1, 133.2, 128.1, 127.0, 125.8, 120.9, 120.8, 64.8, 20.4. IR (KBr, cm^{-1}): ν = 3412, 2181, 1631, 1549, 1445. HRMS (m/z) calcd. for $C_{10}H_{10}BrN_2S$ $[M+H]^+$: 268.9748; found: 268.9743.

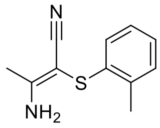

***(Z)*-2bc**

***(Z)*-3-Amino-2-((2-Methylphenyl)thio)-crotononitrile (*(Z)*-2bc).** The mixture of ***(Z)*-2bc** and ***(E)*-2bc** was obtained by a separation of the crude mixture and the ratio of ***(Z)*-2bc** and ***(E)*-2bc** was determined by a ^1H NMR analysis (colorless oil, *Z/E* ratio = 11:1, 80.7 mg, 79%).
The *Z*-isomer was obtained by a further separation of the mixture of ***(Z)*-2bc** and ***(E)*-2bc** with the column chromatography on silica gel (petroleum ether/ethyl acetate = 7:4). ^1H NMR (400 MHz, $CDCl_3$): δ 7.14 – 7.04 (m, 4H), 2.33 (s, 3H), 2.26 (s, 3H). ^{13}C NMR (100 MHz, $CDCl_3$): δ 165.5, 135.3, 133.9, 130.6, 126.7, 125.7, 124.4, 121.6, 64.1, 20.1, 19.8. IR (KBr, cm^{-1}): ν = 3412, 2180, 1635, 1546, 1465. HRMS (m/z) calcd. for $C_{11}H_{12}N_2NaS$ $[M+Na]^+$: 227.0619; found: 227.0613.

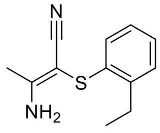

***(Z)*-2bd**

***(Z)*-3-Amino-2-((2-Ethylphenyl)thio)-crotononitrile (*(Z)*-2bd).** The mixture of ***(Z)*-2bd** and ***(E)*-2bd** was obtained by a separation of the crude mixture and the ratio of ***(Z)*-2bd** and ***(E)*-2bd** was determined by a ^1H NMR analysis (colorless oil, *Z/E* ratio = 15:1, 83.0 mg, 76%).
The *Z*-isomer was obtained by a further separation of the mixture of ***(Z)*-2bd** and ***(E)*-2bd** with the column chromatography on silica gel (petroleum ether/ethyl acetate = 7:4). ^1H NMR (400 MHz, $CDCl_3$): δ 7.15 – 7.08 (m, 4H), 2.71 (q, J = 7.5 Hz, 2H), 2.24 (s, 3H), 1.23 (t, J = 7.5 Hz, 3H). ^{13}C NMR (100 MHz, $CDCl_3$): δ 165.6, 141.3, 133.4, 128.8, 126.7, 126.0, 124.8, 121.8, 64.2,

26.4, 20.1, 14.3. IR (KBr, cm^{-1}): ν = 3415, 2180, 1635, 1545, 1462. HRMS (m/z) calcd. for $C_{12}H_{14}N_2NaS$ $[M+Na]^+$: 241.0775; found: 241.0770.

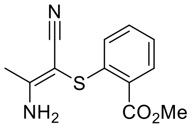

***(Z)*-2be**

***(Z)*-2-((2-amino-1-cyanoprop-1-en-1-yl)thio)methylbenzoate (*(Z)*-2be).** The mixture of ***(Z)*-2be** and ***(E)*-2be** was obtained by a separation of the crude mixture and the ratio of ***(Z)*-2be** and ***(E)*-2be** was determined by a ^{1}H NMR analysis (colorless oil, *Z/E* ratio = 10:1, 62.1 mg, 50%).
The *Z*-isomer was obtained by a further separation of the mixture of ***(Z)*-2be** and ***(E)*-2be** with the column chromatography on silica gel (petroleum ether/ethyl acetate = 3:2). ^{1}H NMR (400 MHz, $CDCl_3$): δ 8.01 (d, J = 7.8 Hz, 1H), 7.46 (dd, J = 8.2, 8.3 Hz, 1H), 7.34 (d, J = 8.2 Hz, 1H), 7.21 (dd, J = 7.5, 7.6 Hz, 1H), 3.93 (s, 3H), 2.37 (s, 3H). ^{13}C NMR (100 MHz, $CDCl_3$): δ 166.9, 165.3, 139.6, 133.0, 131.8, 126.9, 125.1, 125.0, 120.9, 65.8, 52.3, 20.5. IR (KBr, cm^{-1}): ν = 3392, 3313, 2178, 1713, 1640, 1548, 1438. HRMS (m/z) calcd. for $C_{12}H_{12}N_2O_2SNa$ $[M+Na]^+$: 271.0517; found: 271.0512.

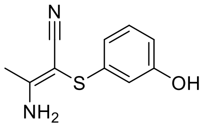

***(Z)*-2c**

***(Z)*-3-Amino-2-((3-Hydroxyphenyl)thio)-crotononitrile (*(Z)*-2c).** The mixture of ***(Z)*-2c** and ***(E)*-2c** was obtained by a separation of the crude mixture and the ratio of ***(Z)*-2c** and ***(E)*-2c** was determined by a ^{1}H NMR analysis (colorless oil, *Z/E* ratio = 13:1, 48.5 mg, 47%).
The *Z*-isomer was obtained by a further separation of the mixture of ***(Z)*-2c** and ***(E)*-2c** with the column chromatography on silica gel (petroleum ether/ethyl acetate = 1:2). ^{1}H NMR (400 MHz, $CDCl_3$): δ 7.14 (dd, J = 7.8, 7.8 Hz, 1H), 6.79 (d, J = 7.6 Hz, 1H), 6.74 (s, 1H), 6.66 (d, J = 8.2 Hz, 1H), 6.01 (br s, 1H), 2.29 (s, 3H). ^{13}C NMR (100 MHz, $CDCl_3$): δ 165.0, 156.7, 136.6, 130.3, 121.2, 118.4, 113.5, 112.7, 66.1, 20.3. IR (KBr, cm^{-1}): ν = 3639, 3598, 3442, 3343, 2181, 1623, 1587, 1549, 1440. HRMS (m/z) calcd. for $C_{10}H_{10}N_2NaOS$ $[M+Na]^+$: 229.0412; found: 229.0406.

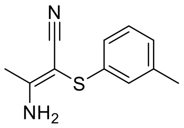

***(Z)*-2d**

***(Z)*-3-Amino-2-((3-Methylphenyl)thio)-crotononitrile (*(Z)*-2d).** The mixture of ***(Z)*-2d** and ***(E)*-2d** was obtained by a separation of the crude mixture and the ratio of ***(Z)*-2d** and ***(E)*-2d** was determined by a ^{1}H NMR analysis (colorless oil, *Z/E* ratio = 12:1, 85.8 mg, 84%).
The *Z*-isomer was obtained by a further separation of the mixture of ***(Z)*-2d** and ***(E)*-2d** with the column chromatography on silica gel (petroleum ether/ethyl acetate = 7:4). ^{1}H NMR (400 MHz, $CDCl_3$): δ 7.17 – 7.14 (m, 1H), 7.02 – 6.96 (m, 3H), 2.30 (s, 3H), 2.27 (s, 3H). ^{13}C NMR (100

MHz, $CDCl_3$): δ 165.1, 139.1, 135.1, 129.1, 127.1, 126.7, 123.2, 121.6, 65.7, 21.5, 20.1. IR (KBr, cm^{-1}): ν = 3421, 2178, 1629, 1546, 1473. HRMS (m/z) calcd. for $C_{11}H_{12}N_2NaS$ $[M+Na]^+$: 227.0619; found: 227.0613.

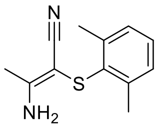

***(Z)*-2e**

***(Z)*-3-Amino-2-((2,6-dimethylphenyl)thio)-crotononitrile (*(Z)*-2e).** The mixture of ***(Z)*-2e** and ***(E)*-2e** was obtained by a separation of the crude mixture and the ratio of ***(Z)*-2e** and ***(E)*-2e** was determined by a ^{1}H NMR analysis (colorless oil, *Z/E* ratio = 18:1, 74.2 mg, 68%).
The *Z*-isomer was obtained by a further separation of the mixture of ***(Z)*-2e** and ***(E)*-2e** with the column chromatography on silica gel (petroleum ether/ethyl acetate = 7:4). ^{1}H NMR (400 MHz, $CDCl_3$): δ 7.19 – 7.15 (m, 1H), 7.04 (br s, 2H), 7.03 – 6.97 (m, 2H), 2.32 (s, 9H). ^{13}C NMR (100 MHz, $CDCl_3$): δ 164.5, 139.1, 135.0, 129.1, 127.1, 126.8, 123.3, 121.2, 66.6, 21.5, 20.3. IR (KBr, cm^{-1}): ν = 3422, 2179, 1630, 1545, 1413. HRMS (m/z) calcd. for $C_{12}H_{14}N_2NaS$ $[M+Na]^+$: 241.0775; found: 241.0770.

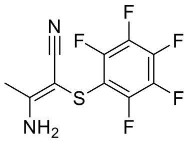

***(Z)*-2f**

***(Z)*-3-Amino-2-((Pentafluorophenyl)thio)-crotononitrile (*(Z)*-2f).** The mixture of ***(Z)*-2f** and ***(E)*-2f** was obtained by a separation of the crude mixture and the ratio of ***(Z)*-2f** and ***(E)*-2f** was determined by a ^{1}H NMR analysis (colorless oil, *Z/E* ratio = 9:1, 110.7 mg, 79%).
The *Z*-isomer was obtained by a further separation of the mixture of ***(Z)*-2f** and ***(E)*-2f** with the column chromatography on silica gel (petroleum ether/ethyl acetate = 2:1). ^{1}H NMR (400 MHz, $CDCl_3$): δ 2.21 (s, 3H). ^{13}C NMR (100 MHz, $CDCl_3$): δ 165.6, 148.5 (m), 146.0 (m), 143.3 (m), 140.7 (m), 139.0 (m), 136.4 (m), 120.3, 108.6 (m), 65.9, 20.4. IR (KBr, cm^{-1}): ν = 3458, 2178, 1631, 1553, 1516, 1483. HRMS (m/z) calcd. for $C_{10}H_6F_5N_2S$ $[M+H]^+$: 281.0172; found: 281.0166.

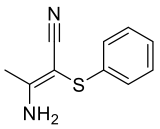

***(Z)*-2g**

***(Z)*-3-Amino-2-(phenylthio)-crotononitrile (*(Z)*-2g).** The mixture of ***(Z)*-2g** and ***(E)*-2g** was obtained by a separation of the crude mixture and the ratio of ***(Z)*-2g** and ***(E)*-2g** was determined by a ^{1}H NMR analysis (colorless oil, *Z/E* ratio = 5:1, 68.5 mg, 72%).
The *Z*-isomer was obtained by a further separation of the mixture of ***(Z)*-2g** and ***(E)*-2g** with the column chromatography on silica gel (petroleum ether/ethyl acetate = 2:1). ^{1}H NMR (400 MHz, $CDCl_3$): δ 7.31 – 7.16 (m, 5H), 2.33 (s, 3H). ^{13}C NMR (100 MHz, $CDCl_3$): δ 164.5, 135.2, 129.2,

126.2, 126.1, 121.0, 66.7, 20.3. IR (KBr, cm^{-1}): v = 3435, 3397, 2178, 1638, 1580, 1544, 1477, 1437. HRMS (m/z) calcd. for $C_{10}H_{10}N_2NaS$ $[M+Na]^+$: 213.0462; found: 213.0457.

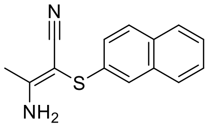

***(Z)*-2h**

***(Z)*-3-Amino-2-(naphthylthio)-crotononitrile (*(Z)*-2h).** The mixture of ***(Z)*-2h** and ***(E)*-2h** was obtained by a separation of the crude mixture and the ratio of ***(Z)*-2h** and ***(E)*-2h** was determined by a ^{1}H NMR analysis (pale yellow oil, *Z/E* ratio = 11:1, 84.1 mg, 70%).
The *Z*-isomer was obtained by a further separation of the mixture of ***(Z)*-2h** and ***(E)*-2h** with the column chromatography on silica gel (petroleum ether/ethyl acetate = 1:1). ^{1}H NMR (400 MHz, $CDCl_3$): δ 7.78 – 7.72 (m, 3H), 7.65 (s, 1H), 7.48 – 7.40 (m, 2H), 7.34 (d, J = 8.6 Hz, 1H), 2.35 (s, 3H). ^{13}C NMR (100 MHz, $CDCl_3$): δ 164.8, 133.8, 132.6, 131.9, 129.0, 127.8, 127.1, 126.8, 125.8, 124.6, 124.2, 121.2, 66.4, 20.3. IR (KBr, cm^{-1}): v = 3426, 2176, 1620, 1547, 1491, 1388. HRMS (m/z) calcd. for $C_{14}H_{12}N_2NaS$ $[M+Na]^+$: 263.0619; found: 263.0613.

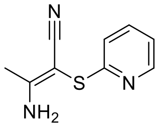

***(Z)*-2i**

***(Z)*-3-Amino-2-(pyridine-2-thio)-crotononitrile (*(Z)*-2i).** The mixture of ***(Z)*-2i** and ***(E)*-2i** was obtained by a separation of the crude mixture and the ratio of ***(Z)*-2i** and ***(E)*-2i** was determined by a ^{1}H NMR analysis (colorless oil, *Z/E* ratio = 8:1, 67.9 mg, 71%).
The *Z*-isomer was obtained by a further separation of the mixture of ***(Z)*-2i** and ***(E)*-2i** with the column chromatography on silica gel (petroleum ether/ethyl acetate = 3:2). ^{1}H NMR (400 MHz, $CDCl_3$): δ 8.41 (d, J = 4.6 Hz, 1H), 7.61 – 7.57 (m, 1H), 7.21 (d, J = 8.0 Hz, 1H), 7.06 (dd, J = 4.9, 5.0 Hz, 1H), 2.35 (s, 3H). ^{13}C NMR (100 MHz, $CDCl_3$): δ 165.5, 158.6, 149.8, 137.2, 121.0, 120.5, 119.7, 64.7, 20.4. IR (KBr, cm^{-1}): v = 3382, 3312, 2168, 1644, 1578, 1548, 1454, 1416. HRMS (m/z) calcd. for $C_9H_9N_3NaS$ $[M+Na]^+$: 214.0415; found: 214.0409.

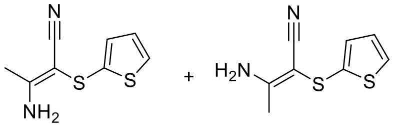

***(Z)*-2j** ***(E)*-2j**

3-Amino-2-(Thienyl-2-thio)-crotononitrile (2j). The mixture of ***(Z)*-2j** and ***(E)*-2j** was obtained by a separation of the crude mixture with the column chromatography on silica gel (petroleum ether/ethyl acetate = 2:1) and the ratio of ***(Z)*-2j** and ***(E)*-2j** was determined by a ^{1}H NMR analysis (pale yellow oil, *Z/E* ratio = 8:1, 68.7 mg, 70%). ^{1}H NMR (400 MHz, $CDCl_3$): *Z*-isomer: δ 7.28 – 7.27 (m, 1H), 7.12 (m, 1H), 6.94 – 6.93 (m, 1H), 5.93 (br s, 2H), 2.15 (s, 3H). *E*-isomer: δ 7.28 – 7.27 (m, 1H), 7.07 (m, 1H), 6.94 – 6.93 (m, 1H), 5.53 (br s, 2H), 2.27 (s, 3H). ^{13}C NMR (100 MHz, $CDCl_3$): *Z*-isomer: δ 164.1, 134.2, 131.6, 128.7, 127.7, 121.6, 70.0, 20.1. *E*-isomer:

δ166.8, 134.2, 130.6, 128.4, 127.7, 121.6, 69.1, 18.9. IR (KBr, cm^{-1}): v = 3443, 3327, 2181, 1624, 1554, 1406, 1219. HRMS (m/z) calcd. for $C_8H_9N_2S_2$ $[M+H]^+$: 197.0207; found: 197.0202.

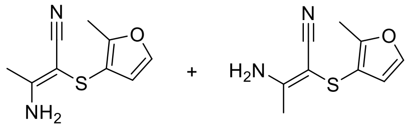

***(Z)*-2k** ***(E)*-2k**

3-Amino-2-(Thienyl-2-thio)-crotononitrile (2k). The mixture of ***(Z)*-2k** and ***(E)*-2k** was obtained by a separation of the crude mixture with the column chromatography on silica gel (petroleum ether/ethyl acetate = 2:1) and the ratio of ***(Z)*-2k** and ***(E)*-2k** was determined by a ^1H NMR analysis (pale yellow oil, Z/E ratio = 8:1, 77.7 mg, 80%). ^1H NMR (400 MHz, $CDCl_3$): *Z*-isomer: δ 7.24 (m, 1H), 6.32 (m, 1H), 5.78 (br s, 2H), 2.38 (s, 3H), 2.15 (s, 3H). *E*-isomer: δ 7.24 (m, 1H), 6.32 (m, 1H), 5.34 (br s, 2H), 2.38 (s, 3H), 2.25 (s, 3H). ^{13}C NMR (100 MHz, $CDCl_3$): *Z*-isomer: δ 163.0, 154.0, 140.7, 121.8, 113.8, 110.6, 69.5, 19.9, 12.0. *E*-isomer: δ 163.0, 154.0, 140.7, 121.8, 113.6, 110.6, 69.5, 19.9, 12.0. IR (KBr, cm^{-1}): v = 3453, 3337, 2179, 1626, 1560, 1510, 1432, 1218. HRMS (m/z) calcd. for $C_9H_{10}N_2NaOS$ $[M+Na]^+$: 217.0412; found: 217.0406.

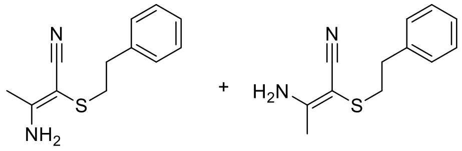

***(Z)*-2l** ***(E)*-2l**

3-Amino-2-(2-Phenylethylthiol)-crotononitrile (2l). The mixture of ***(Z)*-2l** and ***(E)*-2l** was obtained by a separation of the crude mixture with the column chromatography on silica gel (petroleum ether/ethyl acetate = 7:3) and the ratio of ***(Z)*-2l** and ***(E)*-2l** was determined by a ^1H NMR analysis (pale yellow oil, *Z/E* ratio = 2:1, 57.9 mg, 53%). ^1H NMR (400 MHz, $CDCl_3$): *Z*-isomer: δ 7.31 – 7.27 (m, 2H), 7.23 – 7.19 (m, 3H), 5.48 (br s, 1H), 2.91 – 2.85 (m, 4H), 2.20 (s, 3H). *E*-isomer: δ 7.31 – 7.27 (m, 2H), 7.23 – 7.19 (m, 3H), 5.21 (br s, 1H), 2.91 – 2.85 (m, 4H), 2.25 (s, 3H). ^{13}C NMR (100 MHz, $CDCl_3$): *Z*-isomer: δ 163.3, 140.0, 128.6, 128.5, 126.5, 121.6, 67.7, 36.3, 35.8, 20.1. *E*-isomer: δ 159.3, 140.0, 128.6, 128.5, 126.5, 120.7, 67.7, 36.3, 35.8, 19.4. IR (KBr, cm^{-1}): v = 3443, 2179, 1622, 1557, 1496, 1434, 1364. HRMS (m/z) calcd. for $C_{12}H_{14}N_2SNa$ $[M+Na]^+$: 241.0775; found: 241.0770.

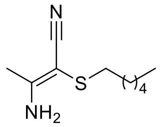

***(Z)*-2m**

***(Z)*-3-Amino-2-(1-Hexanethio)-crotononitrile (*(Z)*-2m).** The mixture of ***(Z)*-2m** and ***(E)*-2m** was obtained by a separation of the crude mixture and the ratio of ***(Z)*-2m** and ***(E)*-2m** was determined by a ^1H NMR analysis (colorless oil, *Z/E* ratio = 13:1, 23.8 mg, 24%).

The *Z*-isomer was obtained by a further separation of the mixture of ***(Z)*-2m** and ***(E)*-2m** with the column chromatography on silica gel (petroleum ether/ethyl acetate = 2:1). ^1H NMR (400 MHz, $CDCl_3$): δ 2.60 (t, J = 7.5 Hz, 2H), 2.21 (s, 3H), 1.63 – 1.56 (m, 2H), 1.42 – 1.26 (m, 8H), 0.89 (t,

J = 6.2 Hz, 3H). ^{13}C NMR (100 MHz, $CDCl_3$): δ 162.5, 121.3, 69.0, 35.1, 31.4, 29.5, 28.4, 22.6, 20.1, 14.0. IR (KBr, cm^{-1}): ν = 3442, 2181, 1621, 1559, 1407. HRMS (m/z) calcd. for $C_{10}H_{18}N_2NaS$ $[M+Na]^+$: 221.1088; found: 221.1083.

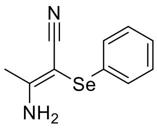

***(Z)*-2p**

***(Z)*-3-Amino-2-(benzeneselenol)-crotononitrile (*(Z)*-2p).** The mixture of ***(Z)*-2p** and ***(E)*-2p** was obtained by a separation of the crude mixture and the ratio of ***(Z)*-2p** and ***(E)*-2p** was determined by a ^1H NMR analysis (colorless oil, *Z/E* ratio = 11:1, 109.1 mg, 92%).
The *Z*-isomer was obtained by a further separation of the mixture of ***(Z)*-2p** and ***(E)*-2p** with the column chromatography on silica gel (petroleum ether/ethyl acetate = 2:1). ^1H NMR (400 MHz, $CDCl_3$): δ 7.36 – 7.34 (m, 2H), 7.27 – 7.19 (m, 3H), 2.29 (s, 3H). ^{13}C NMR (100 MHz, $CDCl_3$): δ 164.1, 130.7, 129.5, 129.1, 126.9, 122.5, 60.7, 20.1. IR (KBr, cm^{-1}): ν = 3434, 3421, 2174, 1622, 1549, 1476, 1436. HRMS (m/z) calcd. for $C_{10}H_{10}N_2NaSe$ $[M+Na]^+$: 260.9907; found: 260.9901.

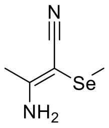

***(Z)*-2q**

***(Z)*-3-Amino-2-(methylselenol)-crotononitrile (*(Z)*-2q).** The mixture of ***(Z)*-2q** and ***(E)*-2q** was obtained by a separation of the crude mixture and the ratio of ***(Z)*-2q** and ***(E)*-2q** was determined by a ^1H NMR analysis (colorless oil, *Z/E* ratio = 12:1, 79.7 mg, 91%).
The *Z*-isomer was obtained by a further separation of the mixture of ***(Z)*-2q** and ***(E)*-2q** with the column chromatography on silica gel (petroleum ether/ethyl acetate = 2:1). ^1H NMR (400 MHz, $CDCl_3$): δ 5.65 (br s, 2H), 2.24 (s, 3H), 2.12 (s, 3H). ^{13}C NMR (100 MHz, $CDCl_3$): δ 162.5, 122.4, 60.8, 19.8, 8.2. IR (KBr, cm^{-1}): ν = 3433, 2174, 1621, 1560, 1398. HRMS (m/z) calcd. for $C_5H_8N_2NaSe$ $[M+Na]^+$: 198.9750; found: 198.9745.

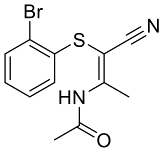

3a

***(Z)*-*N*-(1-((2-bromophenyl)thio)-1-cyanoprop-1-en-2-yl)acetamide (3a)**: colorless oil (132.3 mg, 85%). ^1H NMR (400 MHz, $CDCl_3$): δ 8.67 (br s, 1H), 7.60 – 7.58 (m, 1H), 7.37 – 7.33 (m, 2H), 7.18 – 7.15 (m, 1H), 2.77 (s, 3H), 2.17 (s, 3H). ^{13}C NMR (100 MHz, $CDCl_3$): δ 168.1, 160.9, 133.9, 133.5, 129.1, 128.9, 128.7, 123.0, 117.7, 81.2, 25.3, 20.2. HRMS (m/z) calcd. for $C_{12}H_{10}BrN_2OS$ $[M-H]^-$: 308.9703; found: 308.9712.

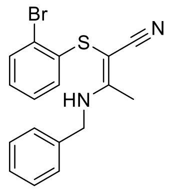

3b

***(Z)*-3-(benzylamino)-2-((2-bromophenyl)thio)but-2-enenitrile (3b)**: colorless oil (39.7 mg, 79%). ^{1}H NMR (400 MHz, $CDCl_3$): δ 7.50 – 7.49 (m, 1H), 7.35 – 7.26 (m, 4H), 7.20 – 7.18 (m, 1H), 7.08 – 7.03 (m, 3H), 6.62 (br s, 1H), 4.43 (d, J = 6.0 Hz, 2H), 2.33 (s, 3H). ^{13}C NMR (100 MHz, $CDCl_3$): δ 166.2, 136.9, 136.1, 133.2, 129.1, 128.1, 128.0, 127.1, 126.5, 125.9, 121.4, 121.1, 63.8, 47.6, 17.6. HRMS (m/z) calcd. for $C_{17}H_{16}BrN_2S$ $[M+H]^+$: 359.0212; found: 359.0219.

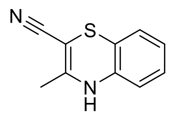

3c

3-methyl-4H-benzo[b][1,4]thiazine-2-carbonitrile (3c). Yellow solid (28.3 mg, yield base on conversion: 70%, conversion of **2bb**: 60%). mp: 140 – 141°C. ^{1}H NMR (400 MHz, $CDCl_3$): δ 7.02 – 6.99 (m, 1H), 6.93 – 6.86 (m, 2H), 6.53 – 6.51 (m, 1H), 6.33 (br s, 1H), 2.13 (s, 3H). ^{13}C NMR (100 MHz, $CDCl_3$): δ 154.1, 138.8, 128.0, 126.9, 125.5, 117.4, 116.7, 115.3, 69.6, 19.9. HRMS (m/z) calcd. for $C_{10}H_9N_2S$ $[M+H]^+$: 189.0481; found: 189.0475.

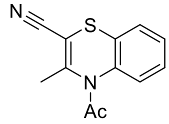

3d

4-acetyl-3-methyl-4H-benzo[b][1,4]thiazine-2-carbonitrile (3d). Colorless oil (19.8 mg, yield base on conversion: 71%, conversion of **3a**: 72%). Notice: The polarity of **3a** and **3d** is very similar. It's hard to separate **3a** and **3d**. ^{1}H NMR (400 MHz, $CDCl_3$): δ 7.43 – 7.39 (m, 2H), 7.34 – 7.30 (m, 2H), 2.55 (s, 3H), 2.16 (s, 3H). ^{13}C NMR (100 MHz, $CDCl_3$): δ 169.1, 156.3, 137.1, 132.5, 129.7, 128.3, 128.1, 128.0, 127.7, 126.3, 114.4, 103.9, 23.2, 21.1. HRMS (m/z) calcd. for $C_{12}H_{11}N_2OS$ $[M+H]^+$: 231.0587; found: 231.0582.

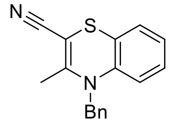

3e

4-benzyl-3-methyl-4H-benzo[b][1,4]thiazine-2-carbonitrile (3e). Yellow oil (16.0 mg, yield base on conversion: 87%, conversion of **3b**: 62%). ^{1}H NMR (400 MHz, $CDCl_3$): δ 7.38 – 7.28 (m, 5H), 7.08 – 6.98 (m, 3H), 6.77 – 6.75 (m, 1H), 4.98 (s, 2H), 2.17 (s, 3H). ^{13}C NMR (100 MHz, $CDCl_3$): δ 159.1, 143.3, 136.4, 129.1, 127.8, 127.7, 127.5, 126.2, 125.4, 123.0, 117.4, 116.0, 52.1, 19.3. HRMS (m/z) calcd. for $C_{17}H_{15}N_2S$ $[M+H]^+$: 279.0950; found: 279.0948.

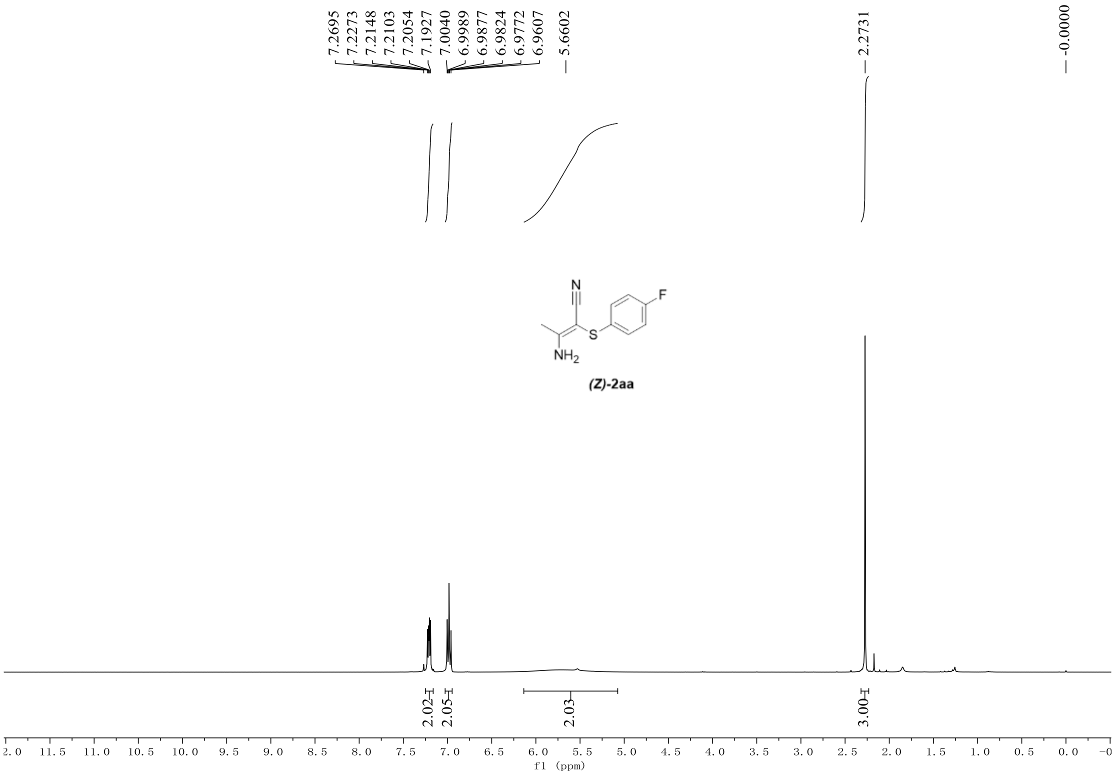

Supplementary Figure 3. ^{1}H NMR spectrum of *(Z)*-2aa

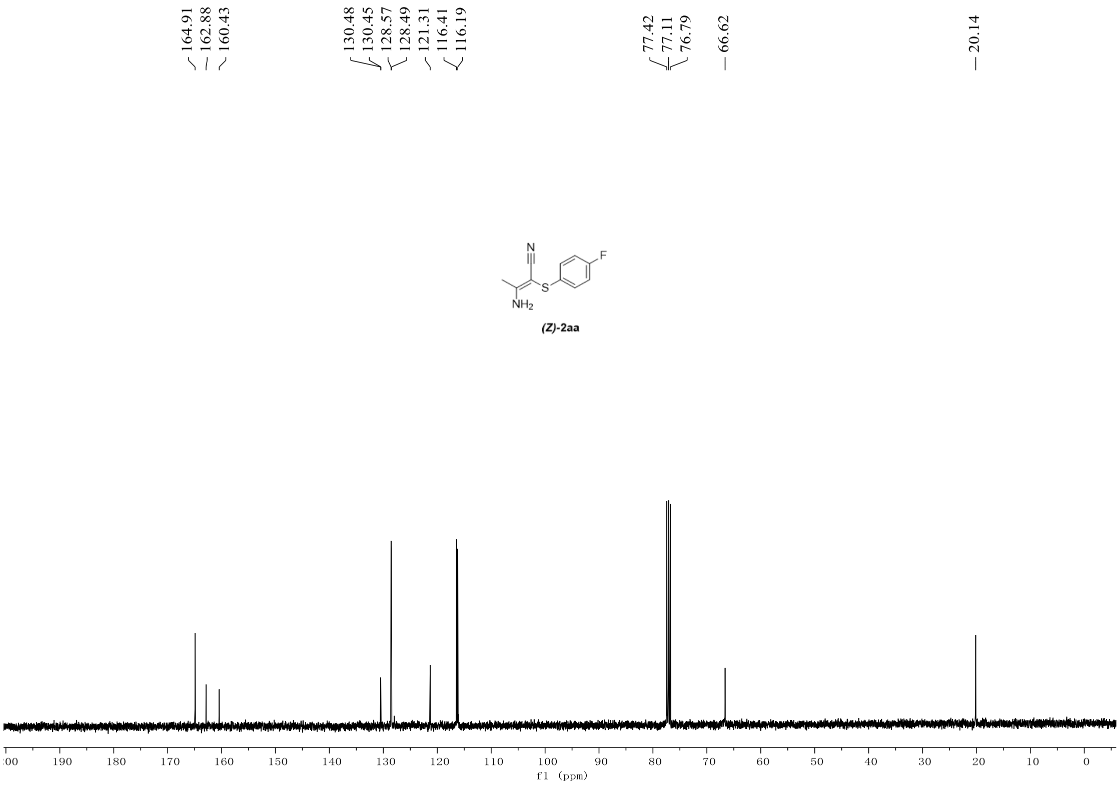

Supplementary Figure 4. ^{13}C NMR spectrum of *(Z)*-2aa

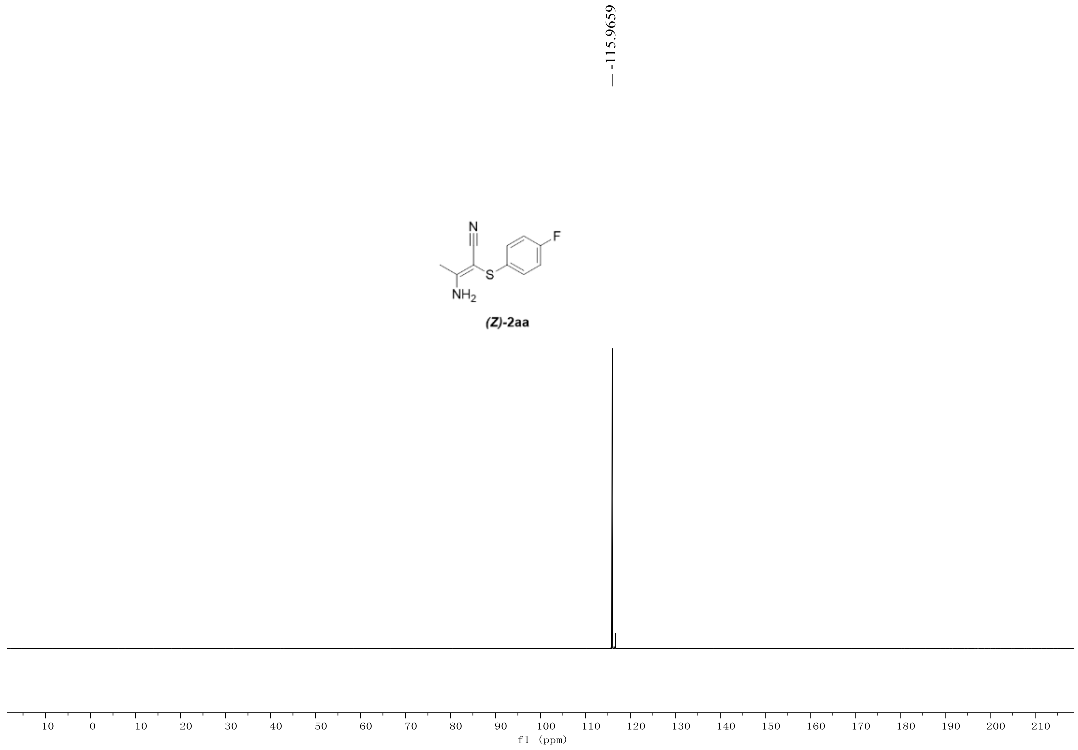

Supplementary Figure 5. ^{19}F NMR spectrum of *(Z)*-2aa

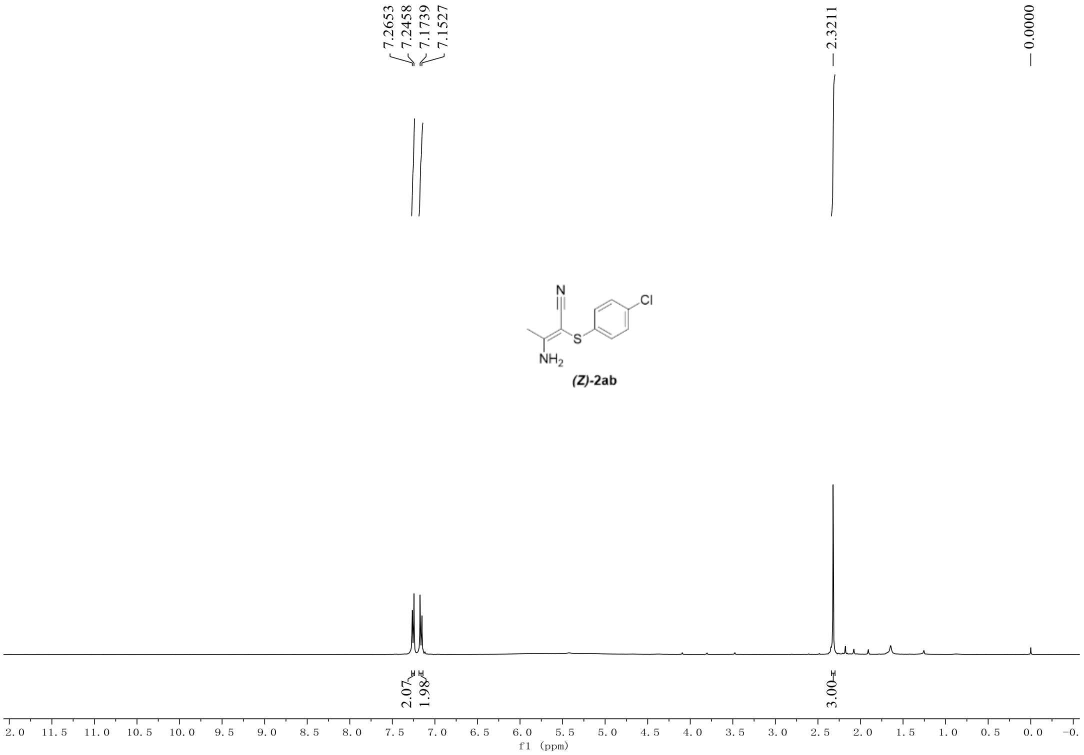

Supplementary Figure 6. ^{1}H NMR spectrum of *(Z)*-2ab

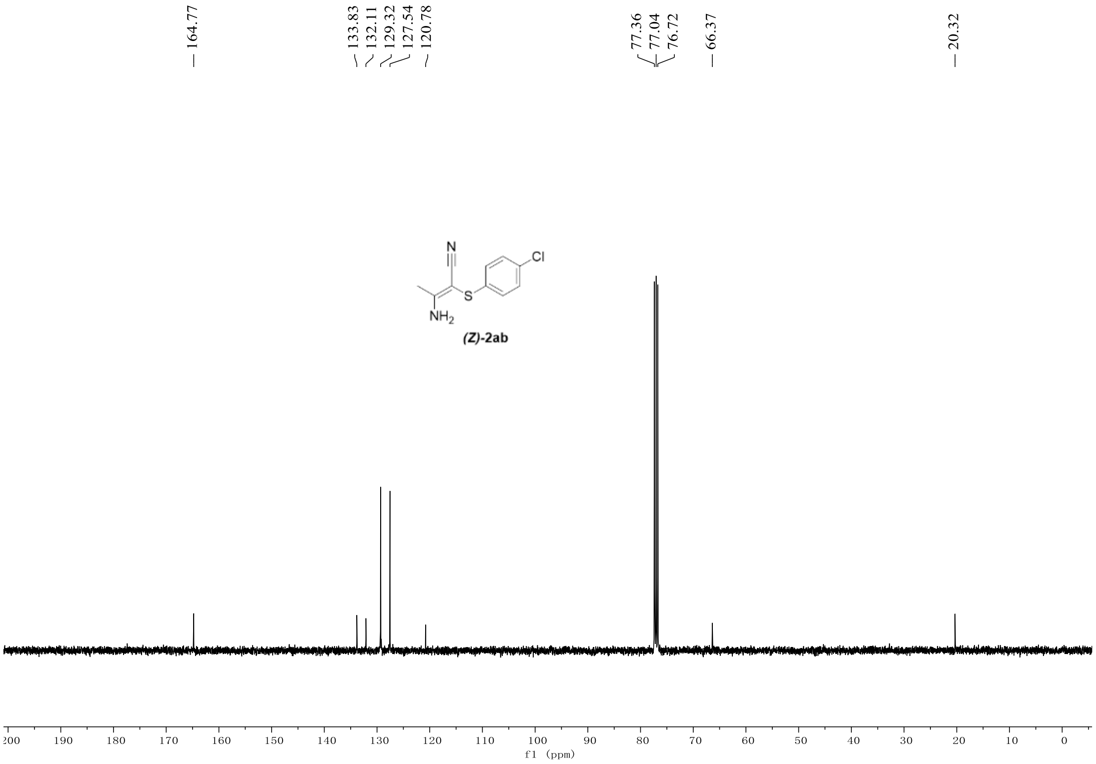

Supplementary Figure 7. ^{13}**C NMR spectrum of** ***(Z)*****-2ab**

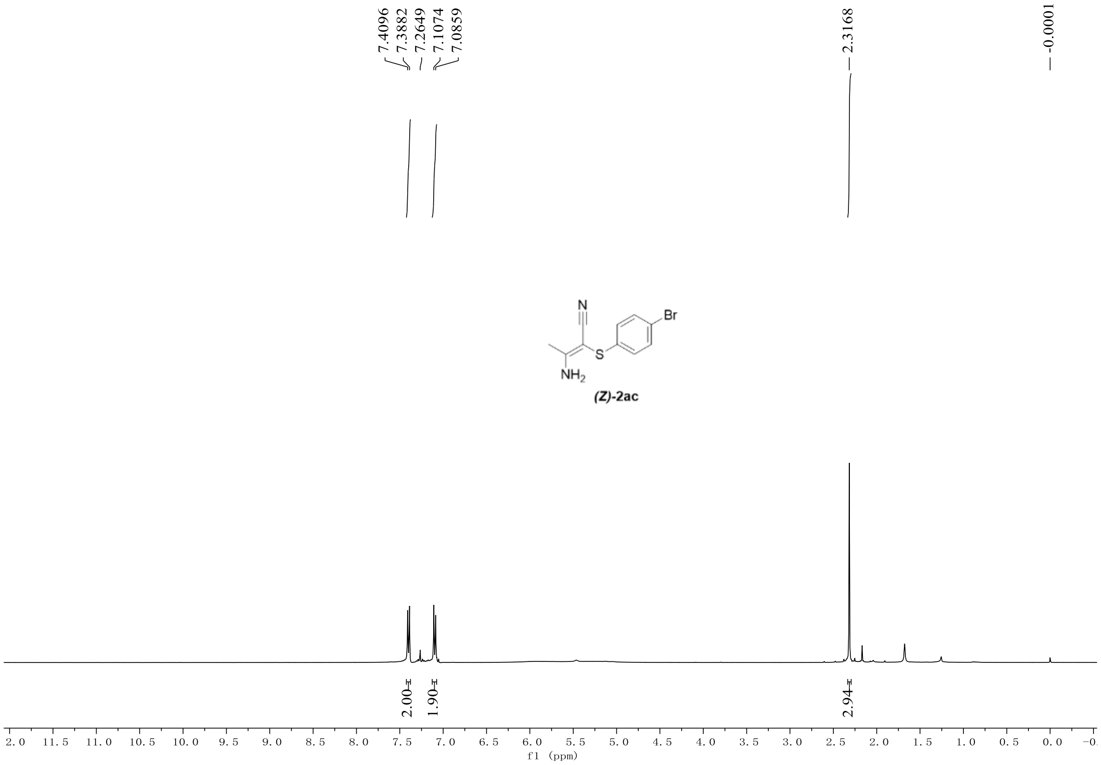

Supplementary Figure 8. ^{1}**H NMR spectrum of** ***(Z)*****-2ac**

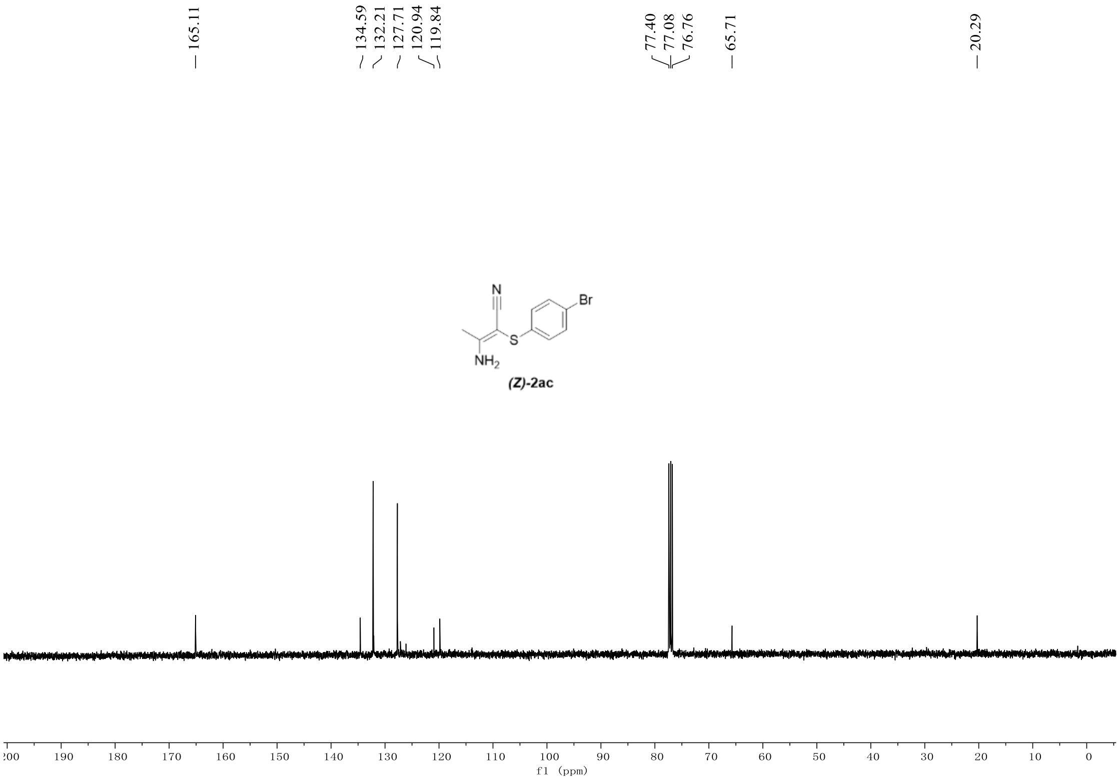

Supplementary Figure 9. ^{13}C NMR spectrum of *(Z)*-2ac

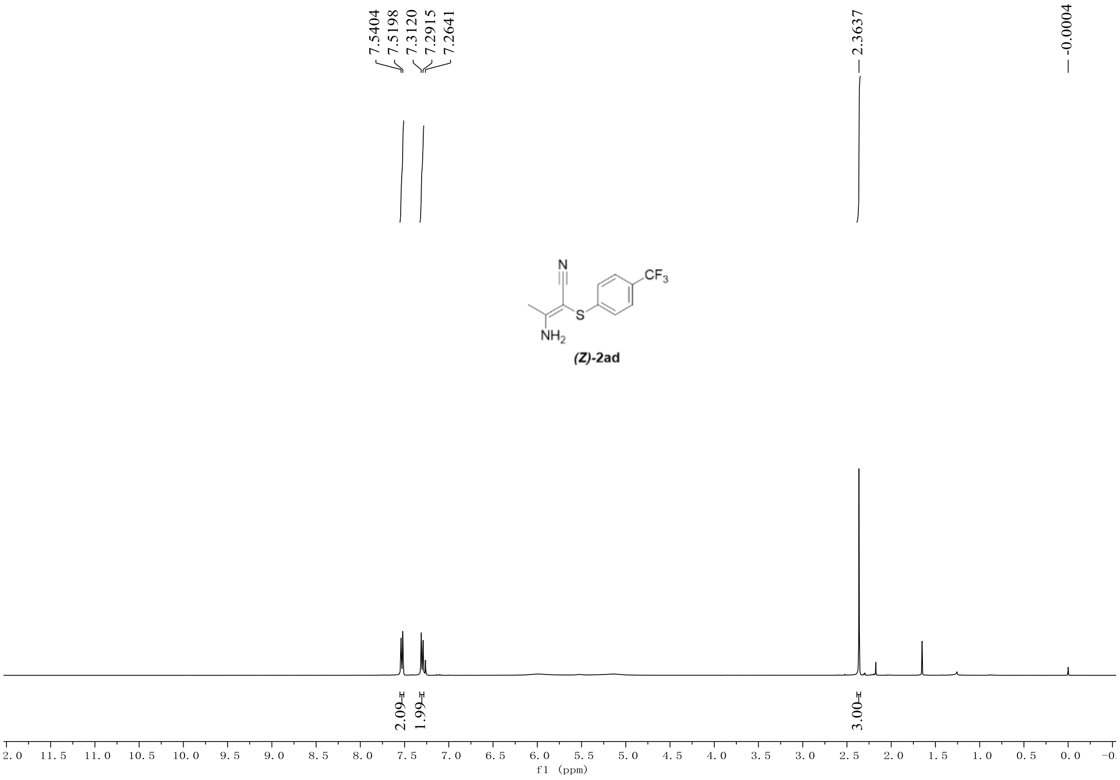

Supplementary Figure 10. ^{1}H NMR spectrum of *(Z)*-2ad

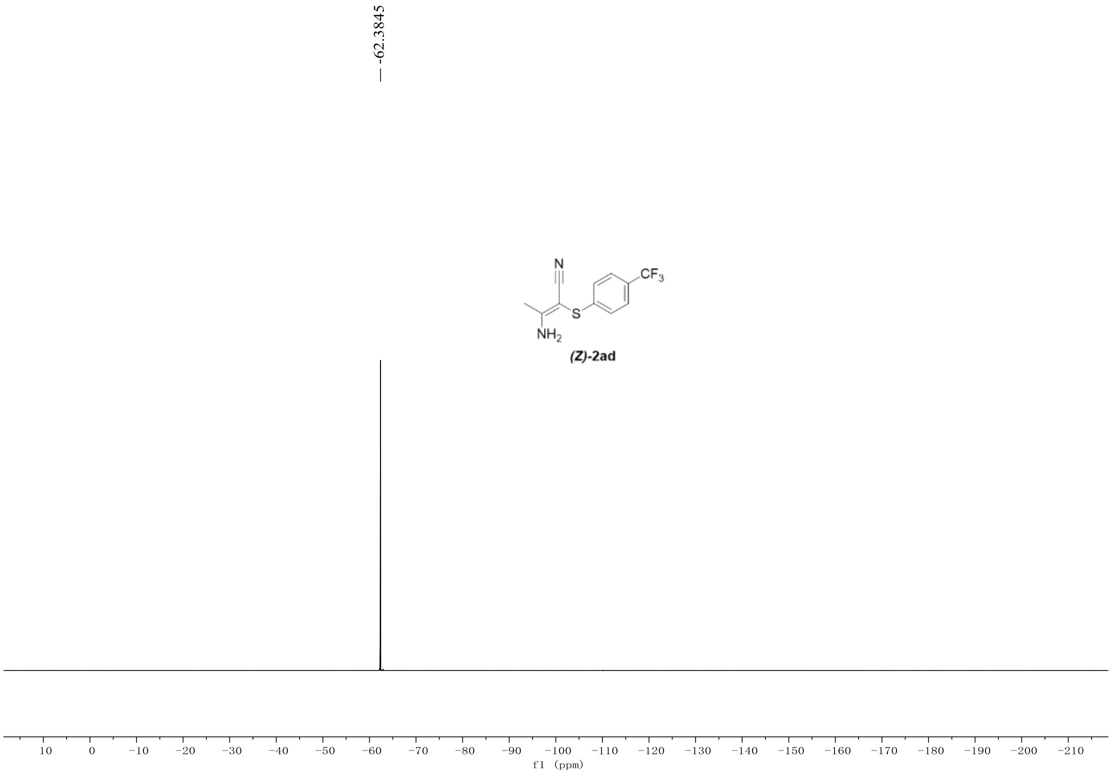

Supplementary Figure 11. ^{19}**F NMR spectrum of *(Z)*-2ad**

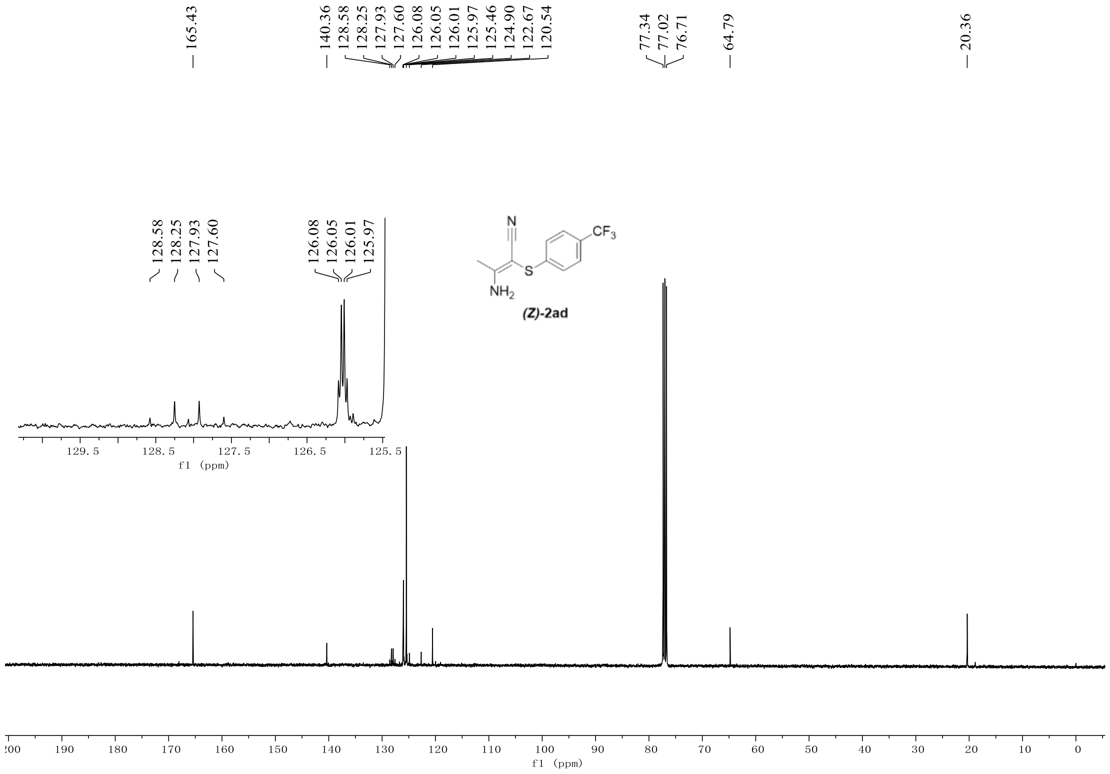

Supplementary Figure 12. ^{13}**C NMR spectrum of *(Z)*-2ad**

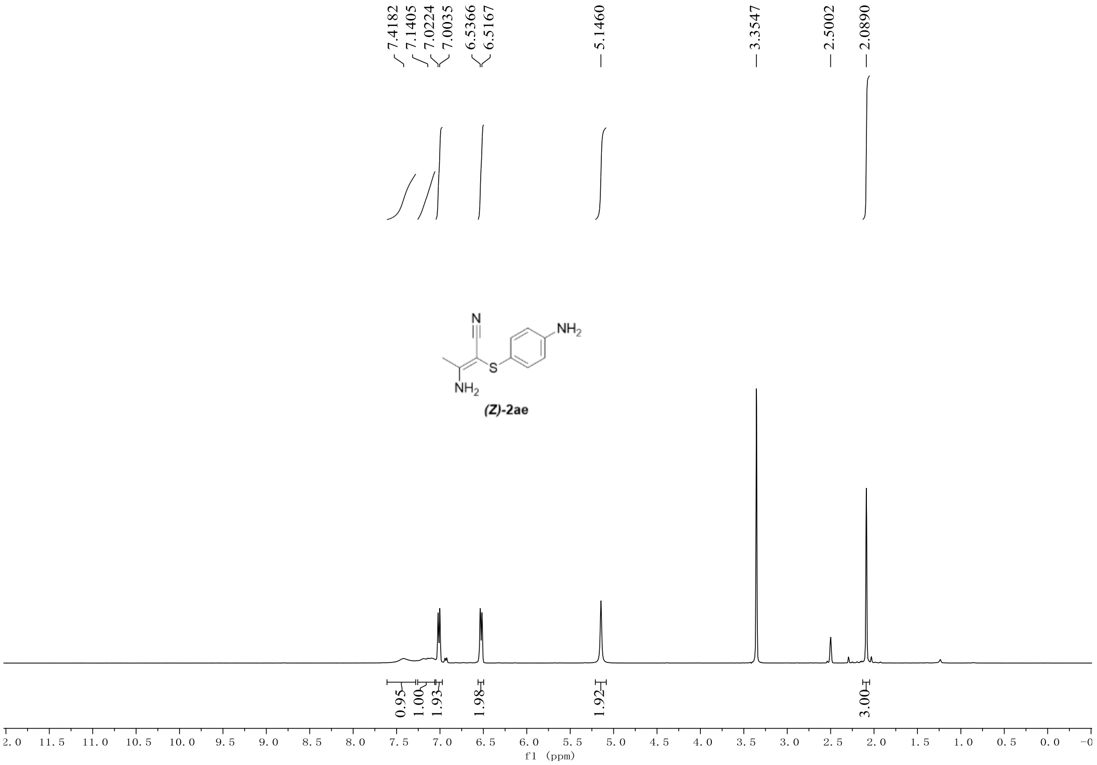

Supplementary Figure 13. ^{1}H NMR spectrum of *(Z)*-2ae

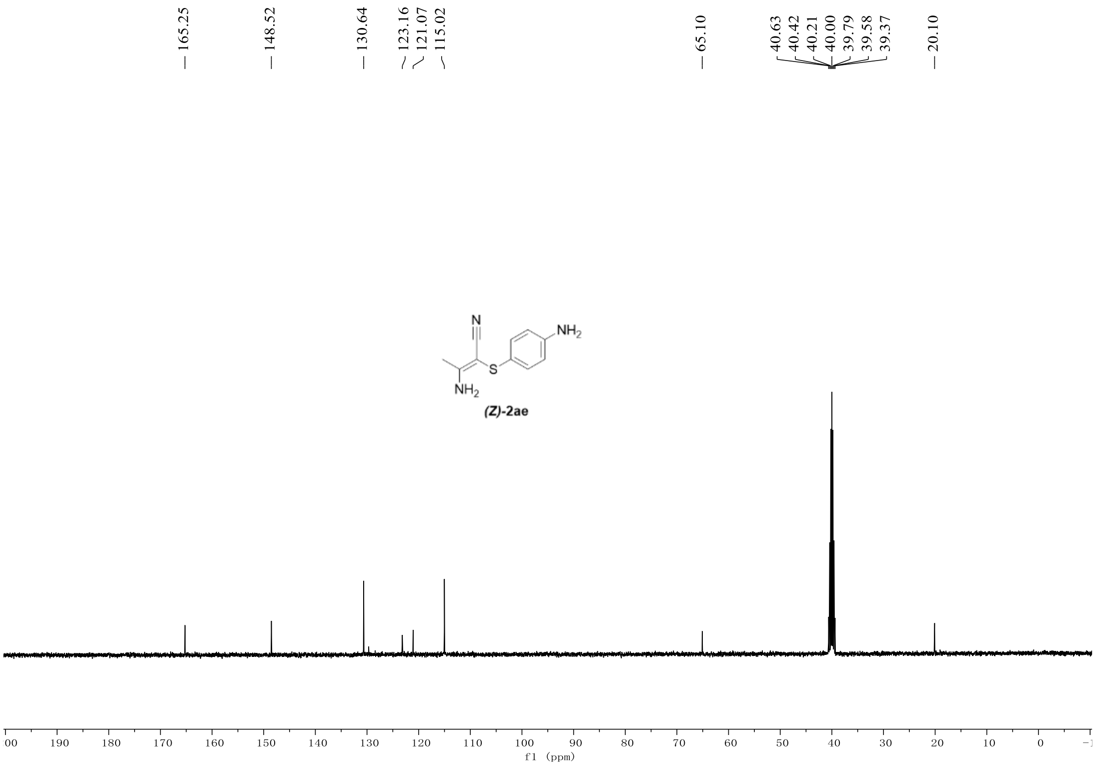

Supplementary Figure 14. ^{13}C NMR spectrum of *(Z)*-2ae

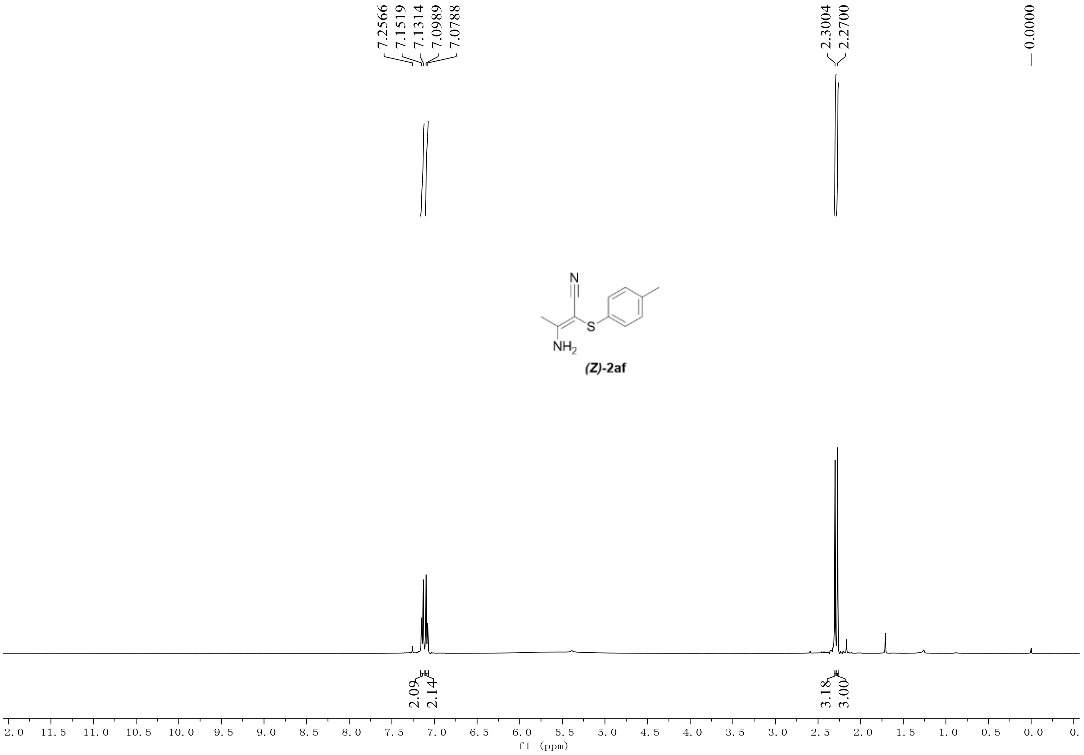

Supplementary Figure 15. ^{1}**H NMR spectrum of** ***(Z)*****-2af**

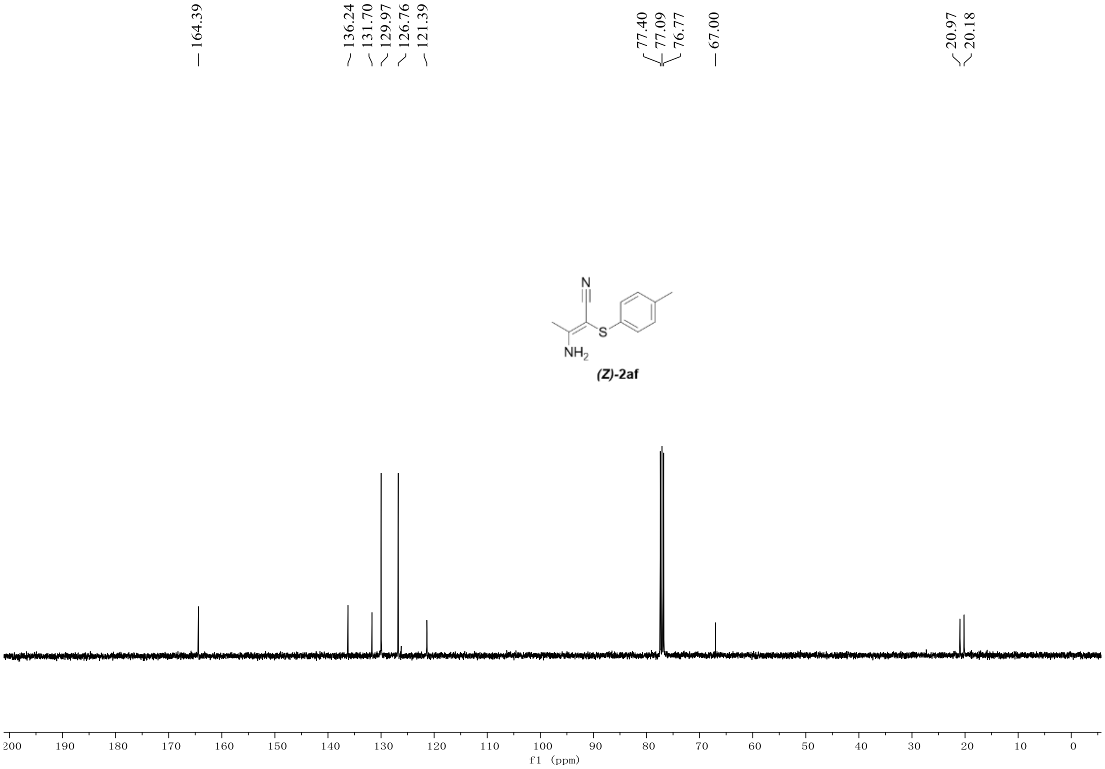

Supplementary Figure 16. ^{13}**C NMR spectrum of** ***(Z)*****-2af**

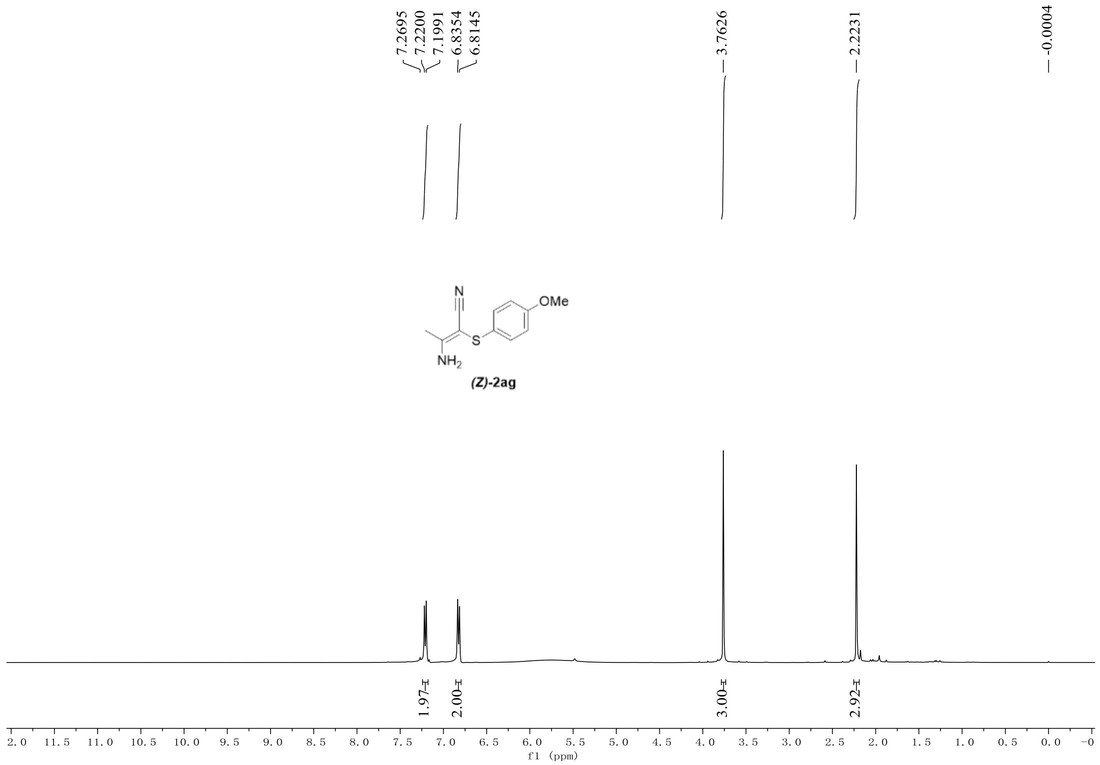

Supplementary Figure 17. ^{1}H NMR spectrum of *(Z)*-2ag

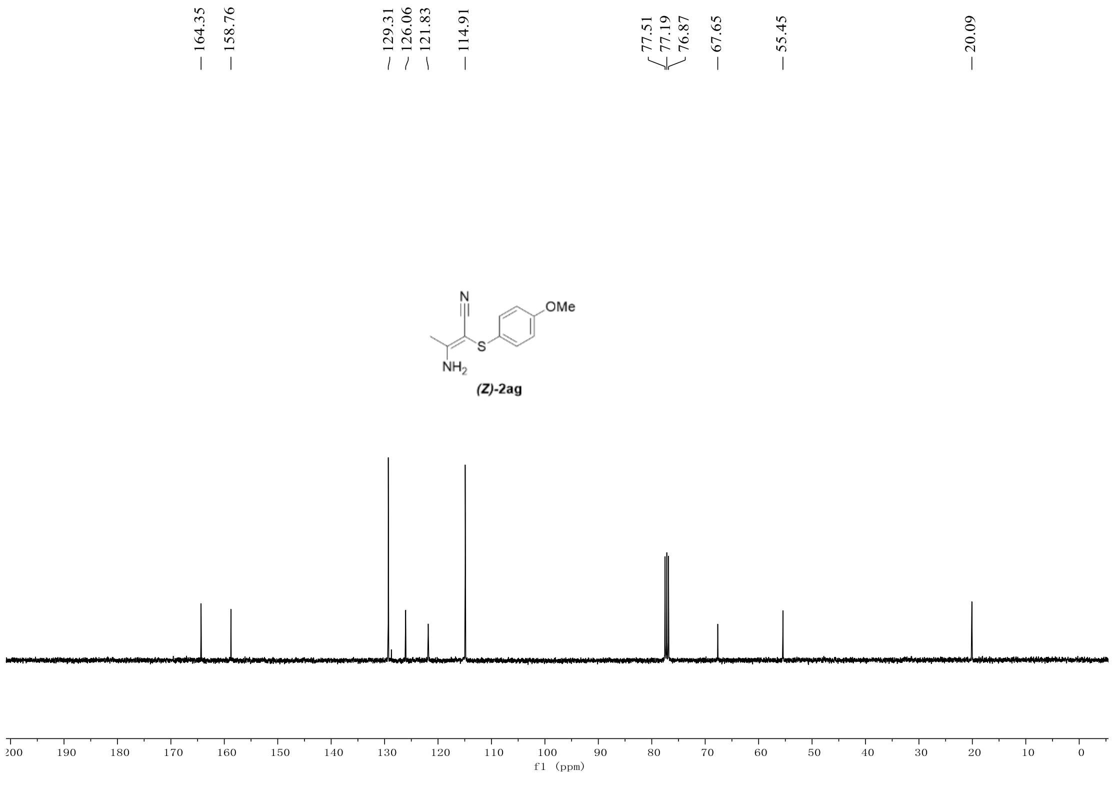

Supplementary Figure 18. ^{13}C NMR spectrum of *(Z)*-2ag

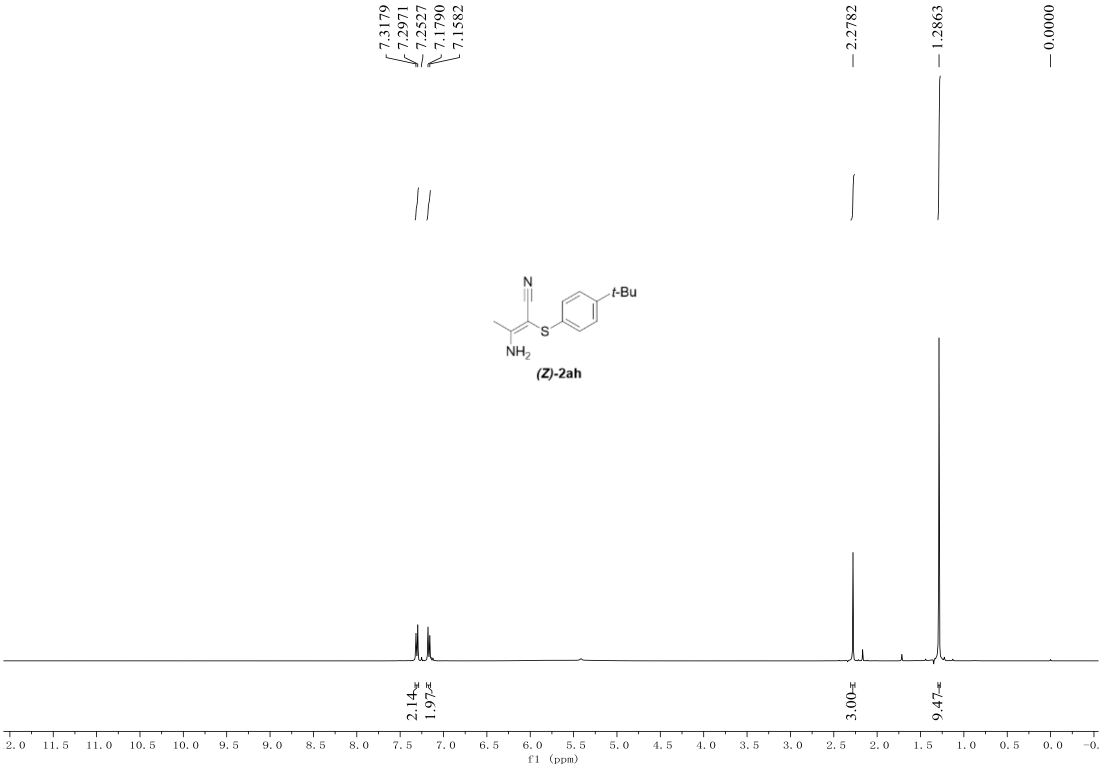

Supplementary Figure 19. ^{1}H NMR spectrum of *(Z)*-2ah

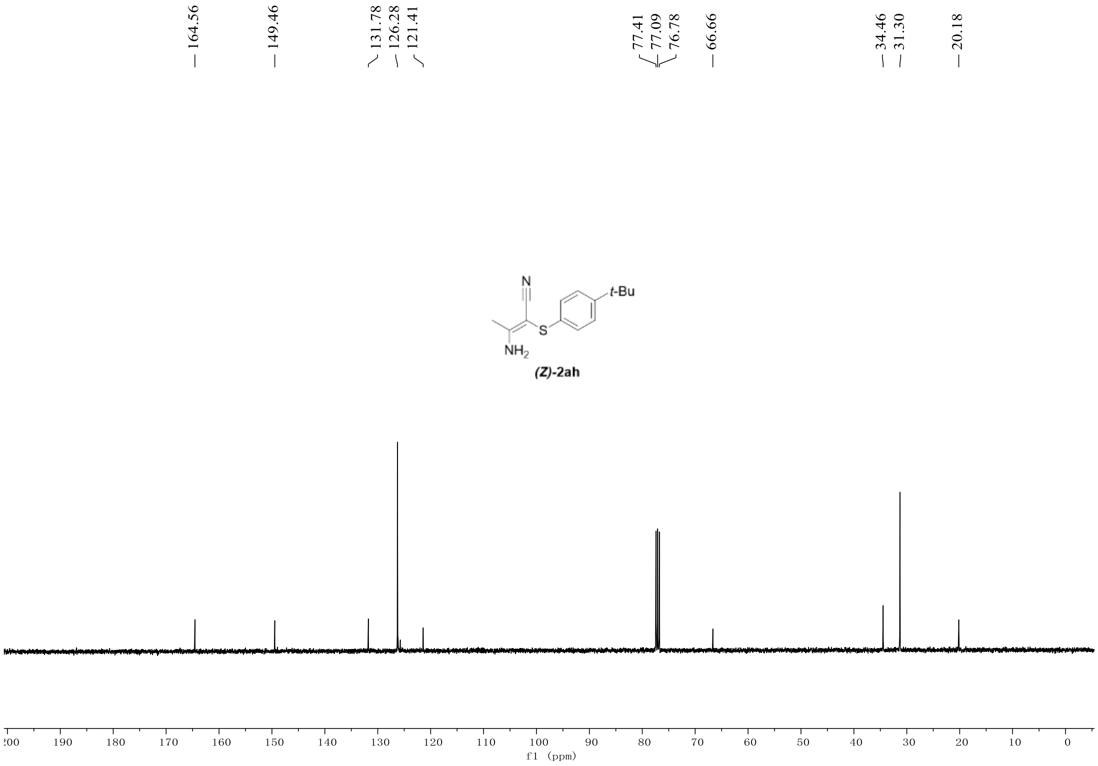

Supplementary Figure 20. ^{13}C NMR spectrum of *(Z)*-2ah

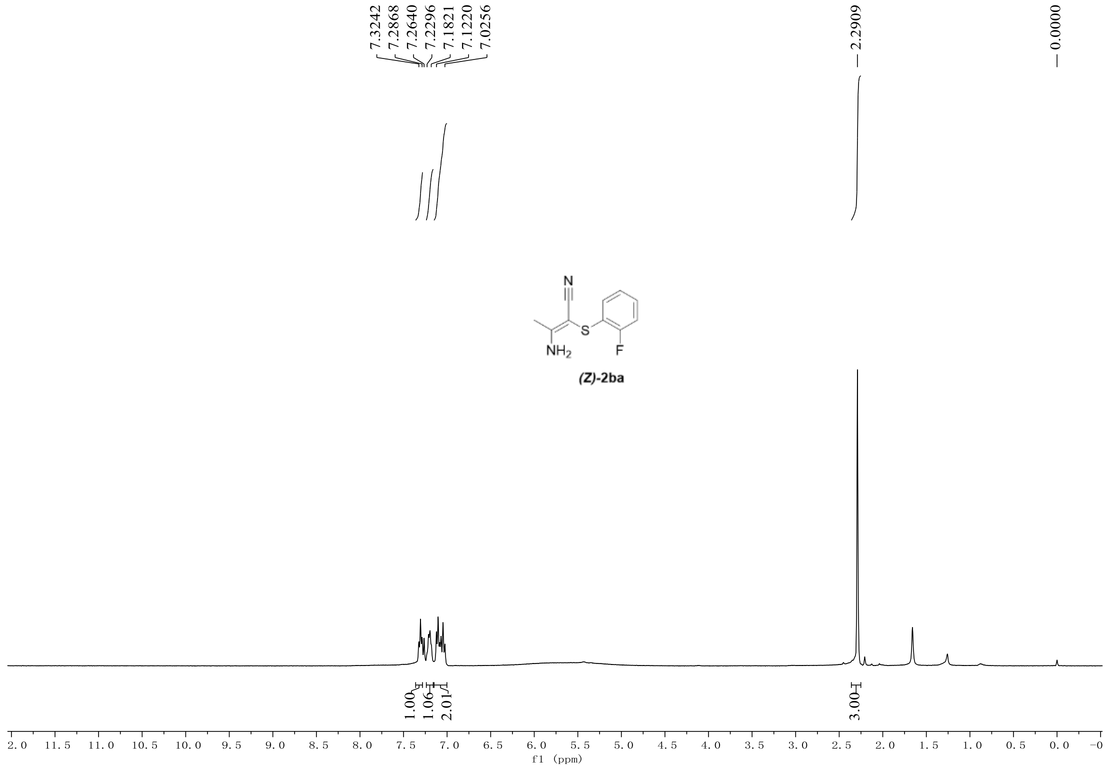

Supplementary Figure 21. ^{1}H NMR spectrum of *(Z)*-2ba

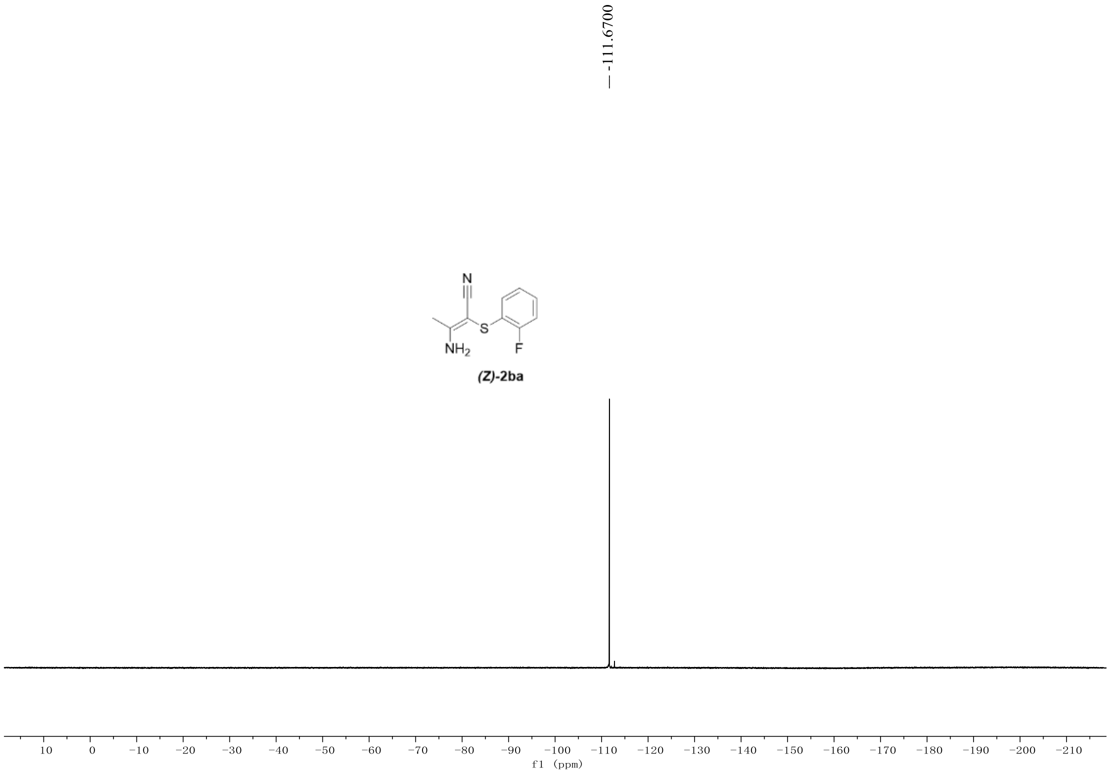

Supplementary Figure 22. ^{19}F NMR spectrum of *(Z)*-2ba

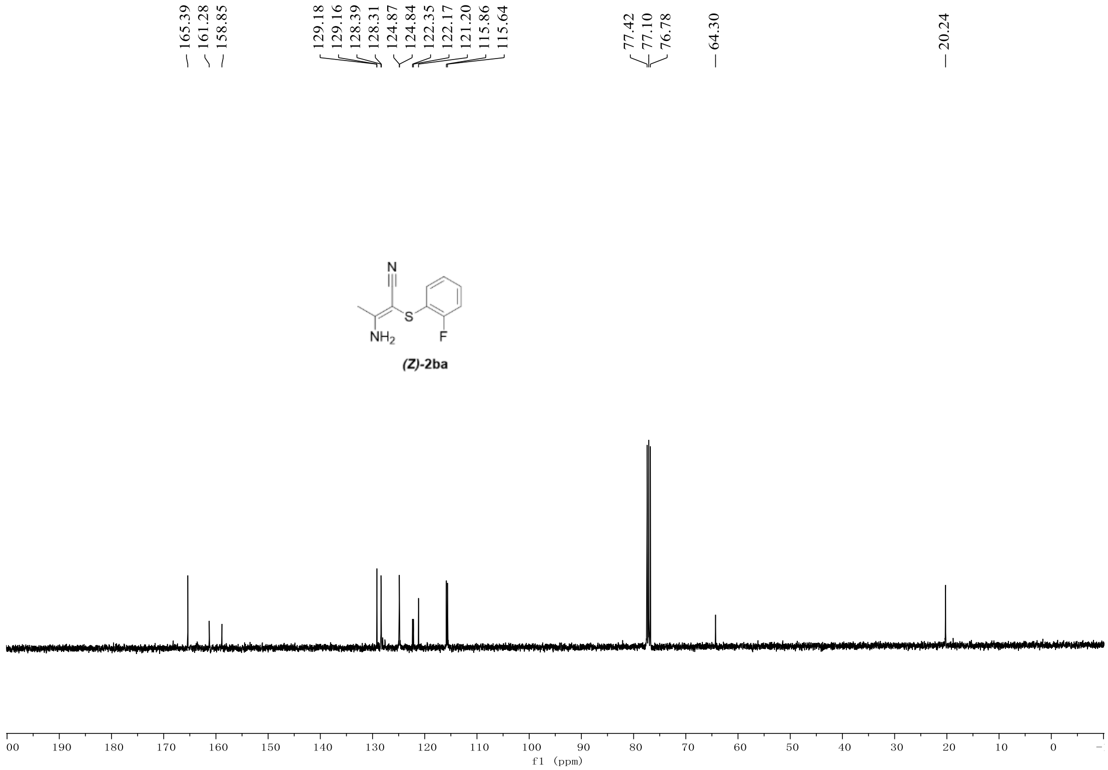

Supplementary Figure 23. ^{13}C NMR spectrum of *(Z)*-2ba

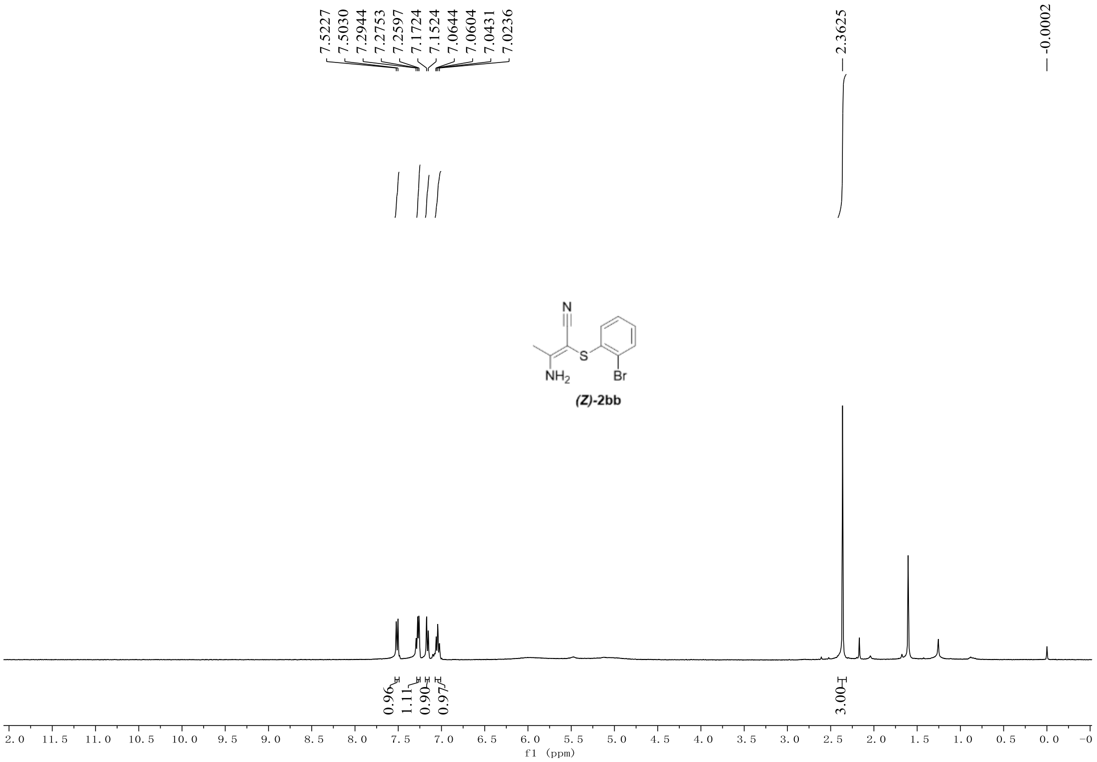

Supplementary Figure 24. ^{1}H NMR spectrum of *(Z)*-2bb

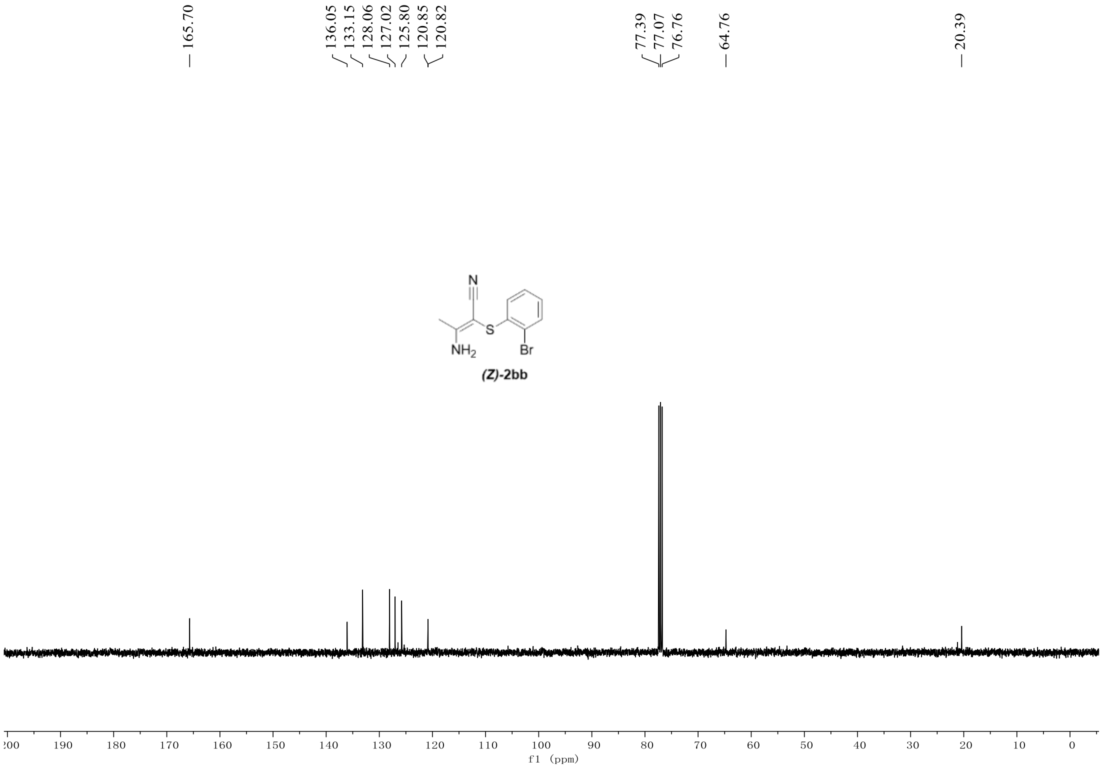

Supplementary Figure 25. ^{13}C NMR spectrum of *(Z)*-2bb

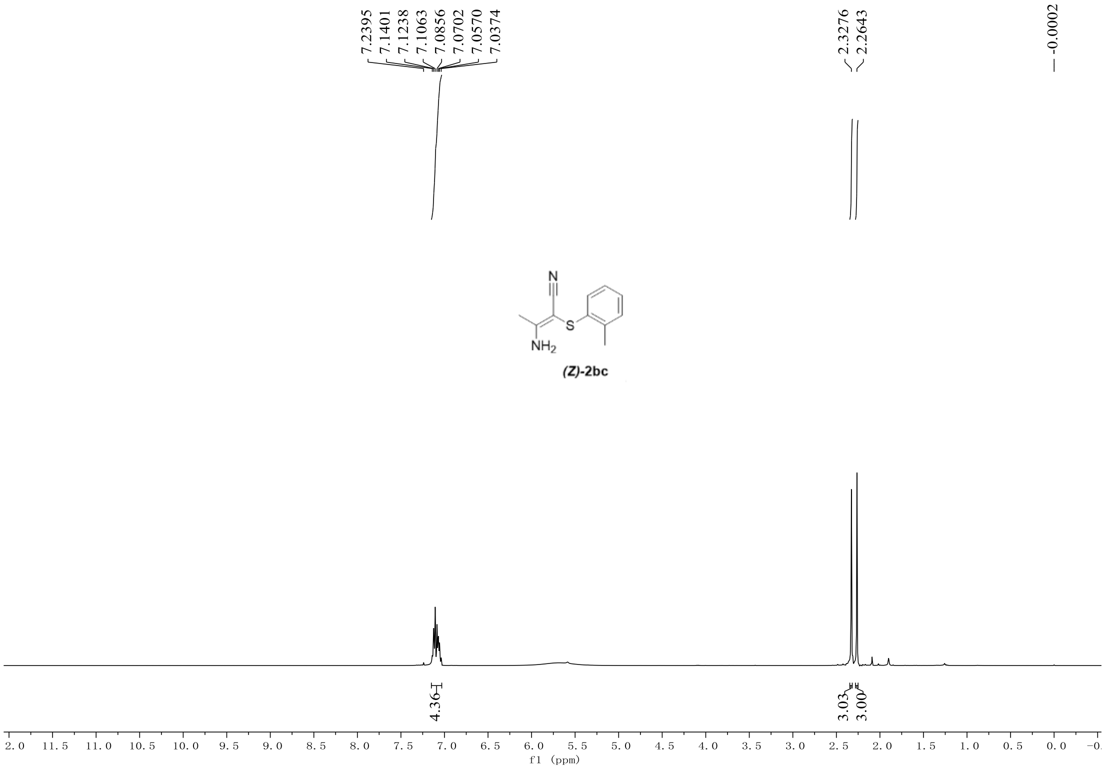

Supplementary Figure 26. ^{1}H NMR spectrum of *(Z)*-2bc

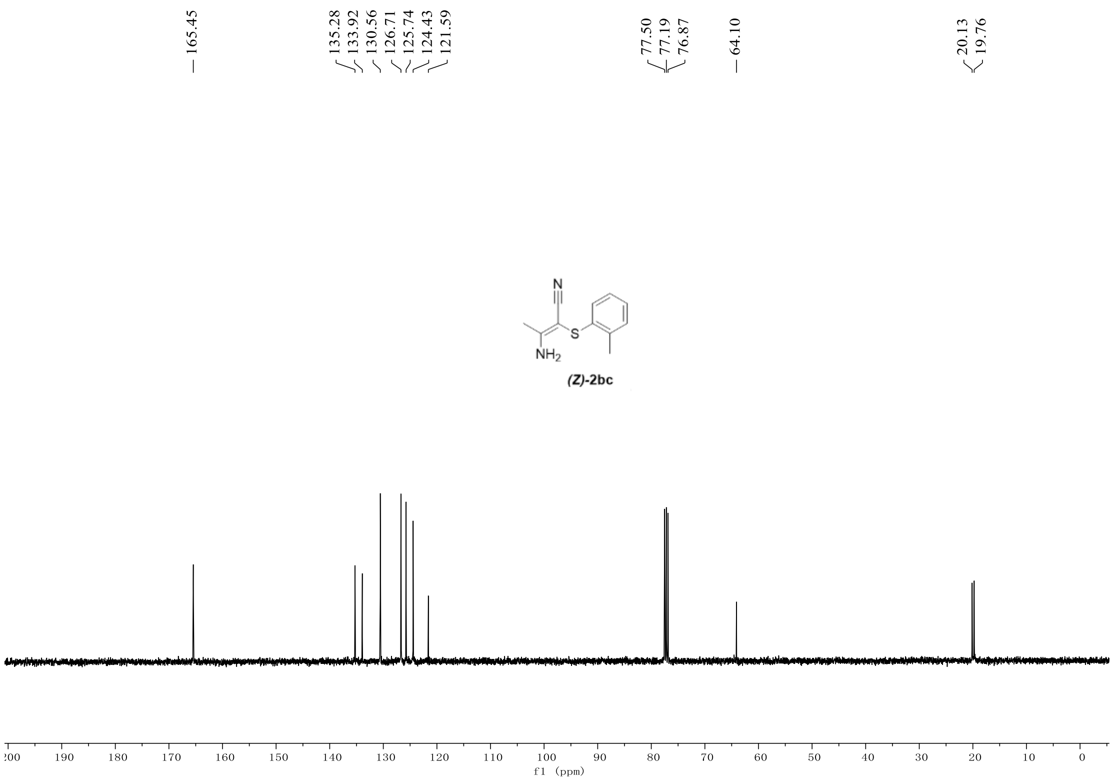

Supplementary Figure 27. ^{13}C NMR spectrum of *(Z)*-2bc

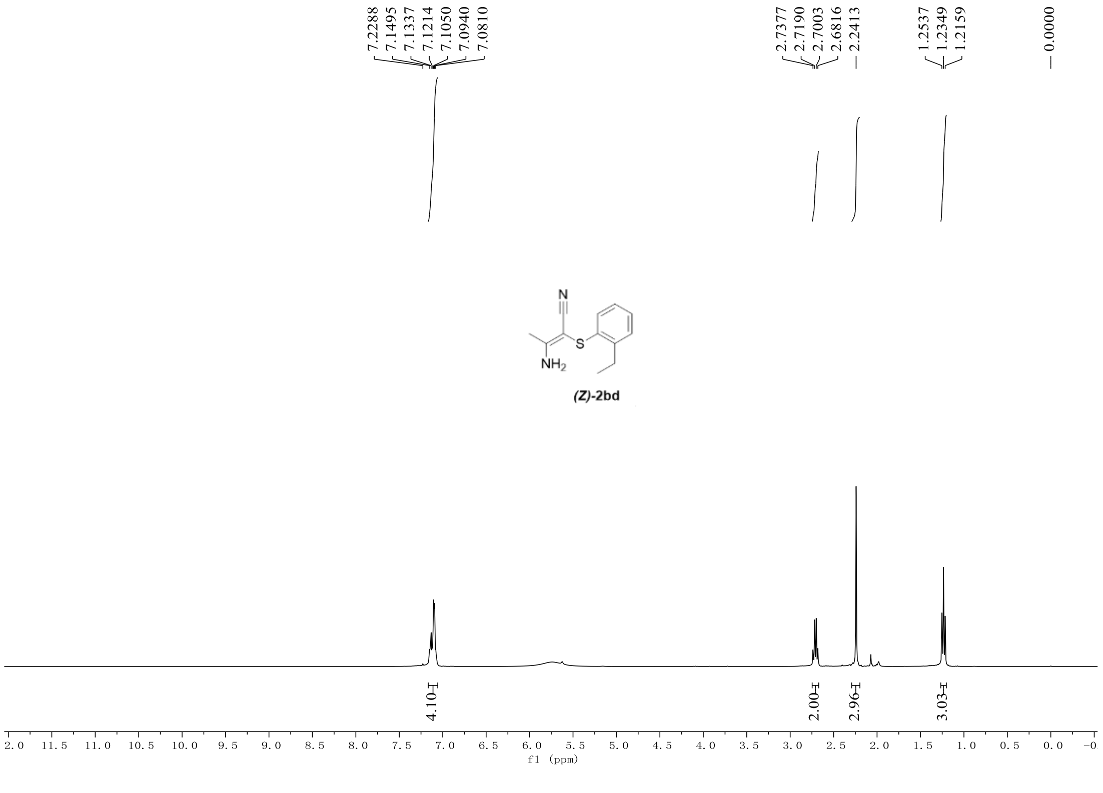

Supplementary Figure 28. ^{1}H NMR spectrum of *(Z)*-2bd

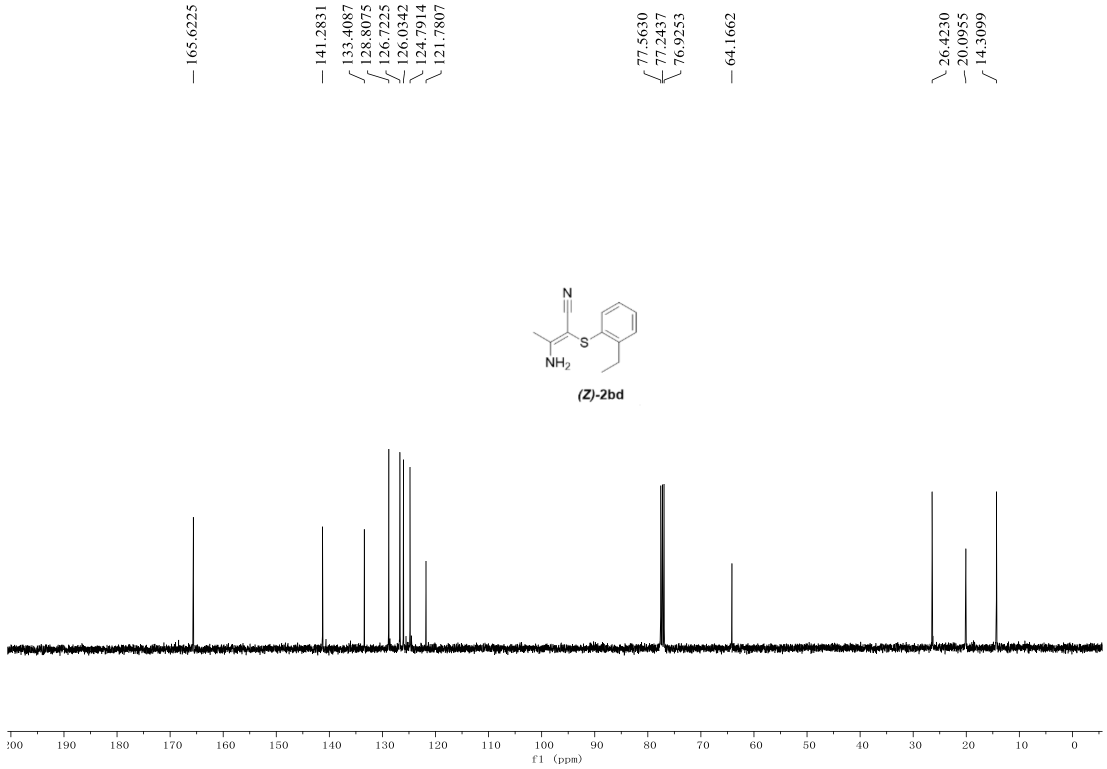

Supplementary Figure 29. ^{13}C NMR spectrum of *(Z)*-2bd

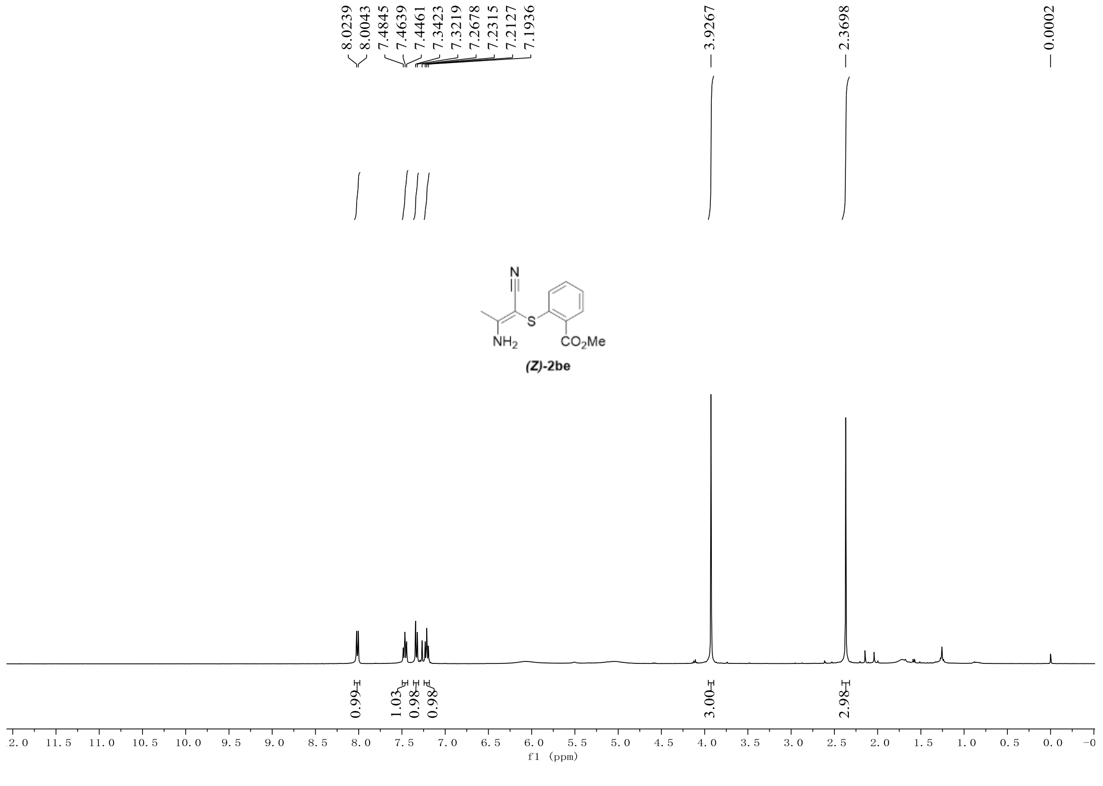

Supplementary Figure 30. ^{1}H NMR spectrum of *(Z)*-2be

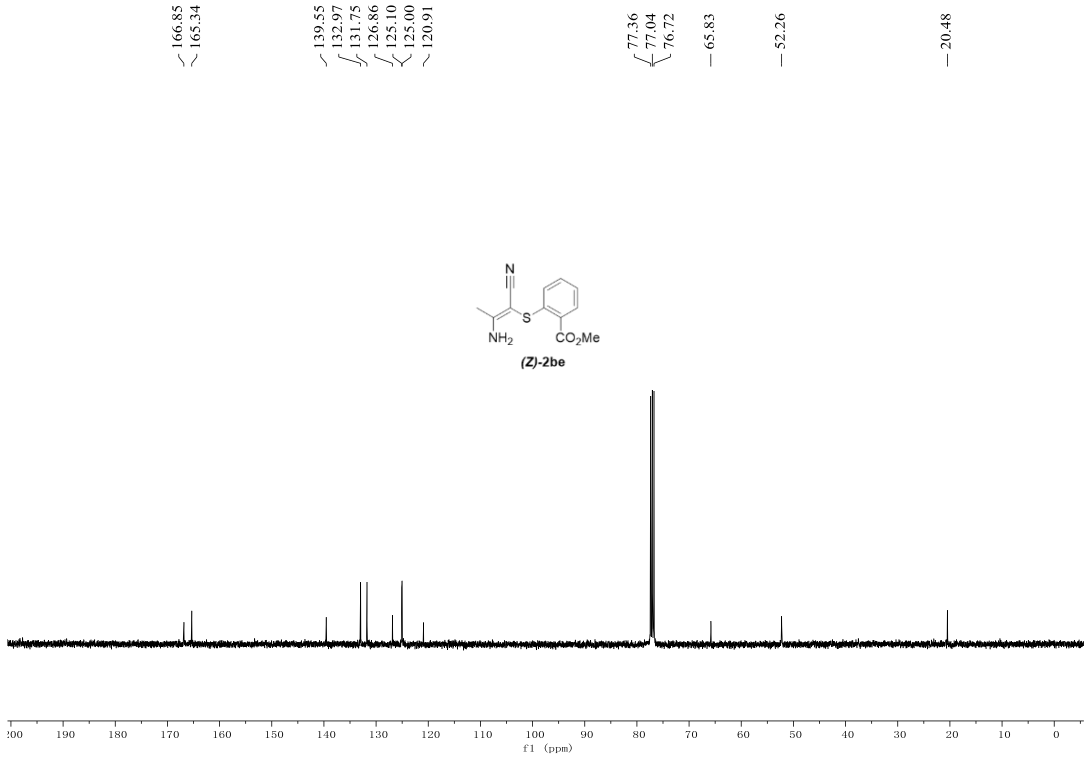

Supplementary Figure 31. ^{13}C NMR spectrum of *(Z)*-2be

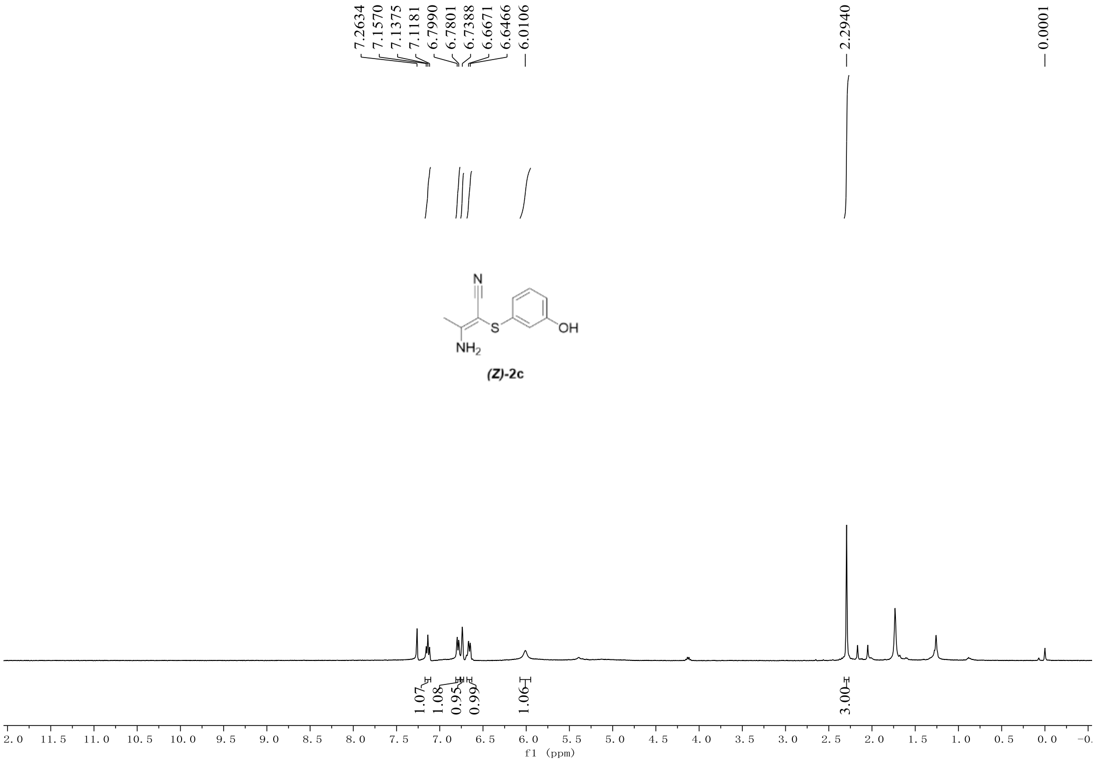

Supplementary Figure 32. ^{1}H NMR spectrum of *(Z)*-2c

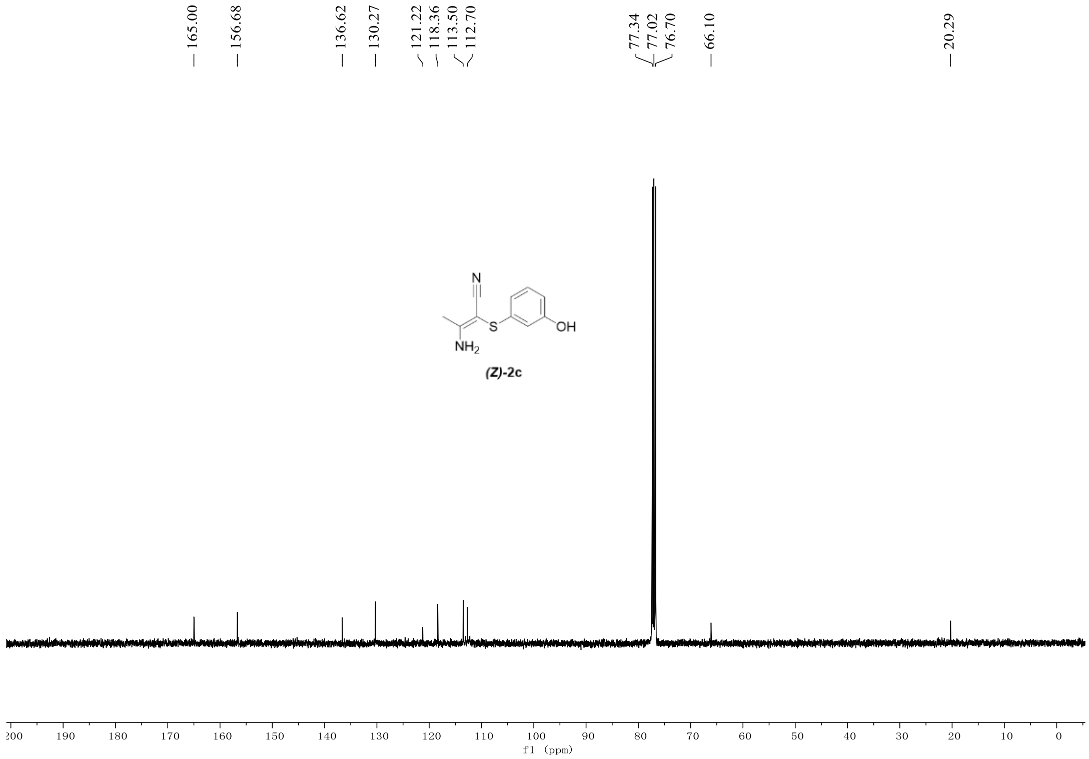

Supplementary Figure 33. ^{13}C NMR spectrum of *(Z)*-2c

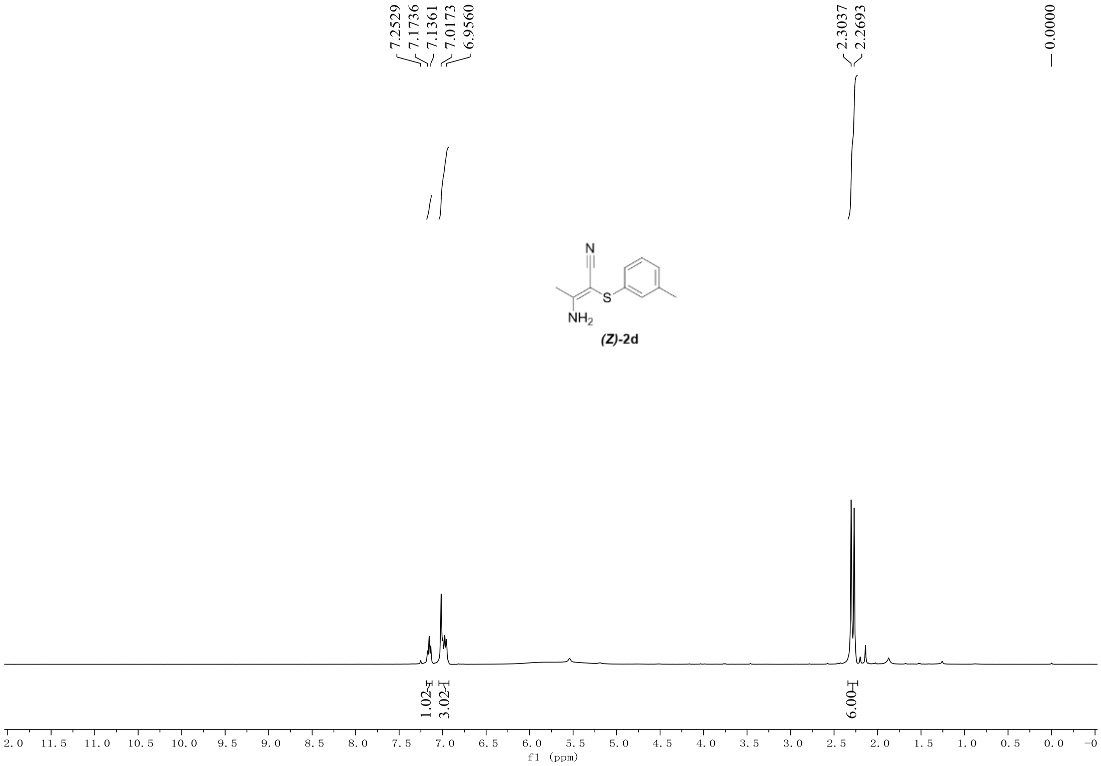

Supplementary Figure 34. ^{1}H NMR spectrum of *(Z)*-2d

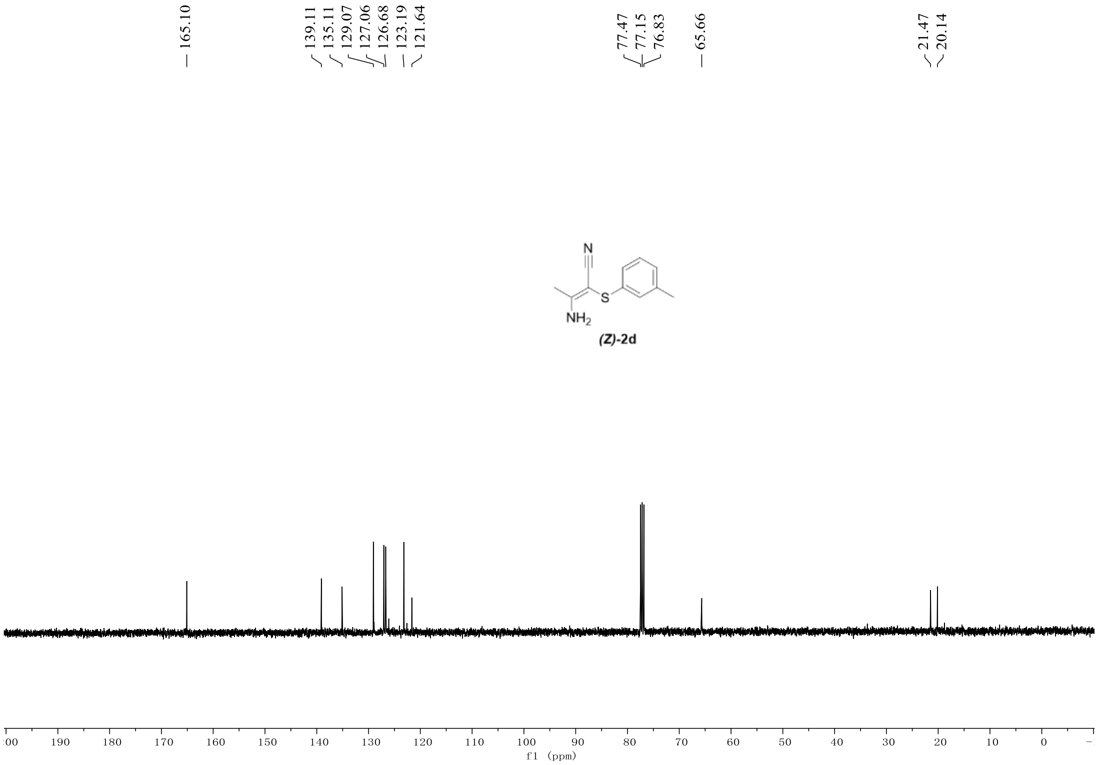

Supplementary Figure 35. ^{13}C NMR spectrum of *(Z)*-2d

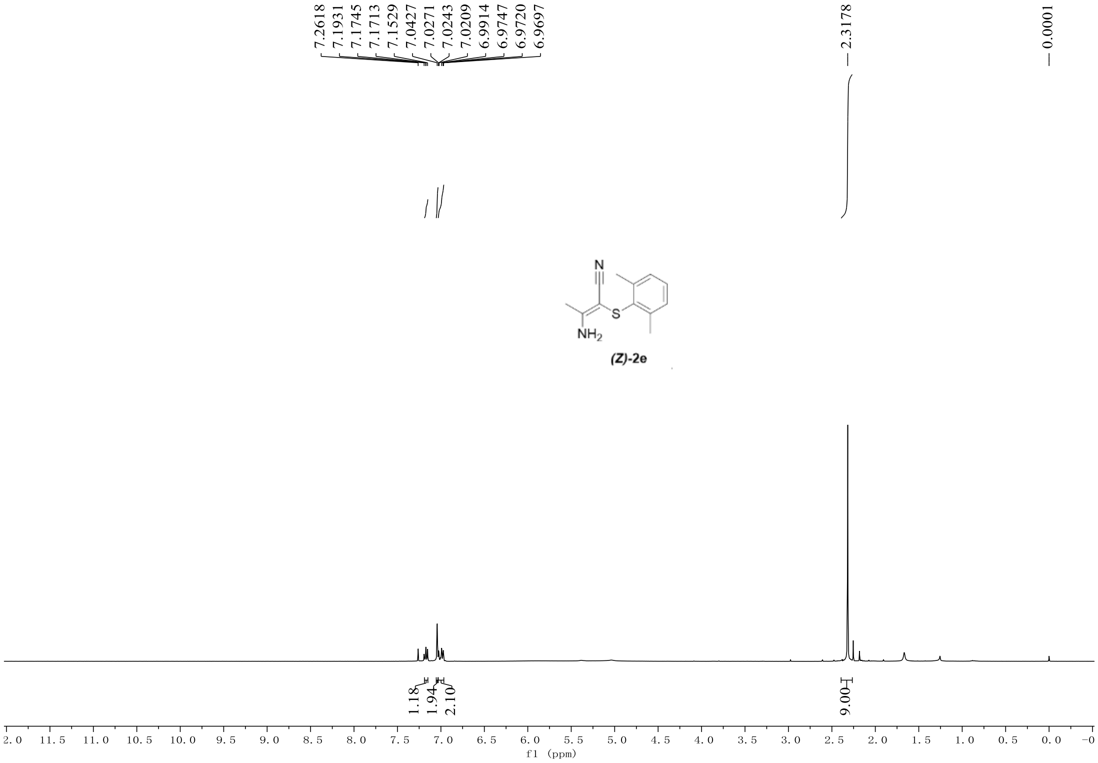

Supplementary Figure 36. ^{1}H NMR spectrum of *(Z)*-2e

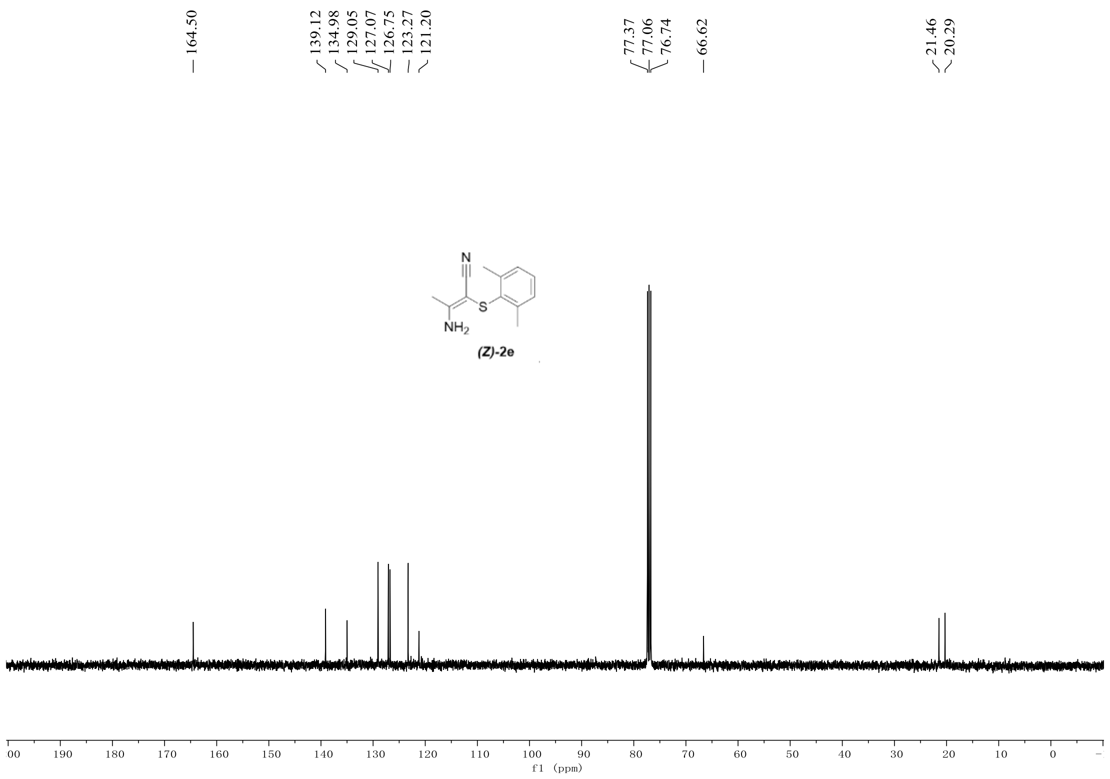

Supplementary Figure 37. ^{13}C NMR spectrum of *(Z)*-2e

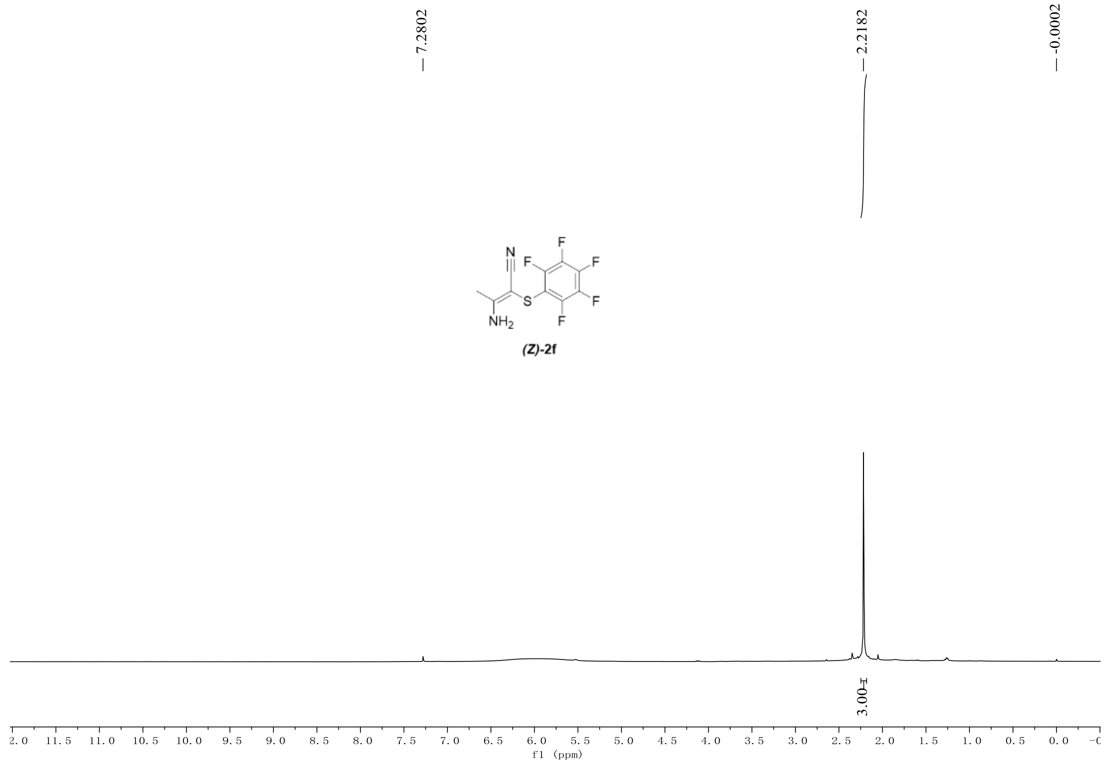

Supplementary Figure 38. ^{1}H NMR spectrum of *(Z)*-2f

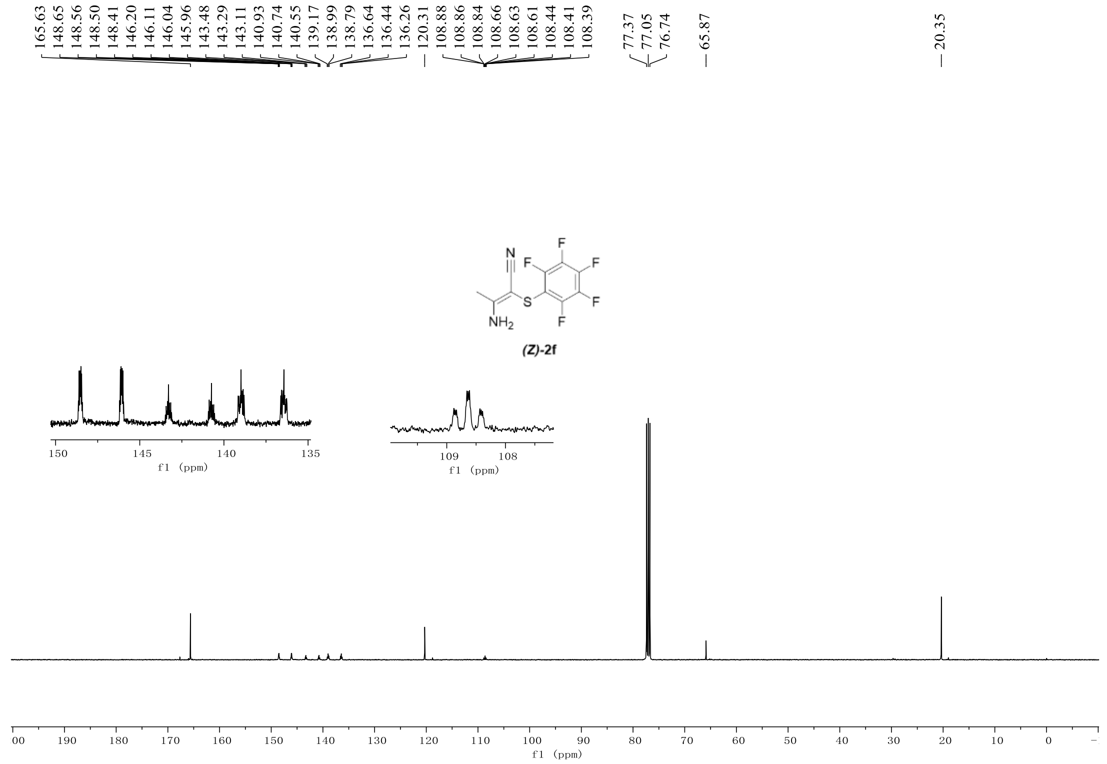

Supplementary Figure 39. ^{13}**C NMR spectrum of** ***(Z)*****-2f**

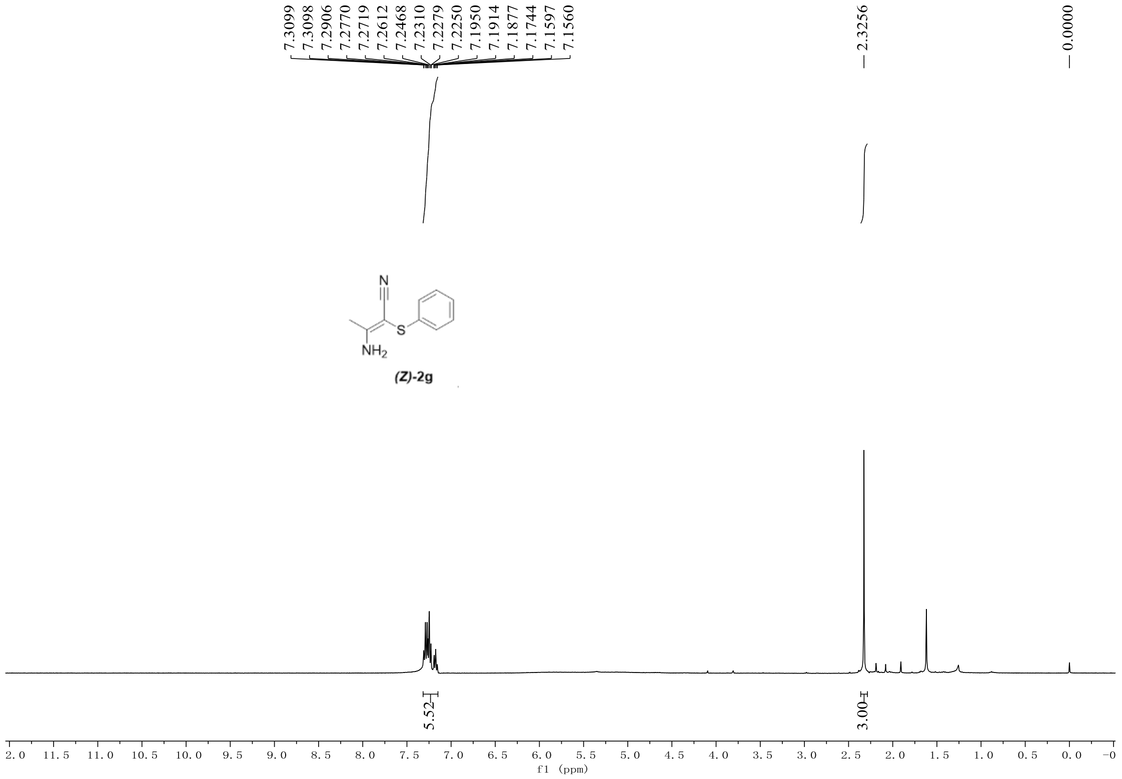

Supplementary Figure 40. ^{1}**H NMR spectrum of** ***(Z)*****-2g**

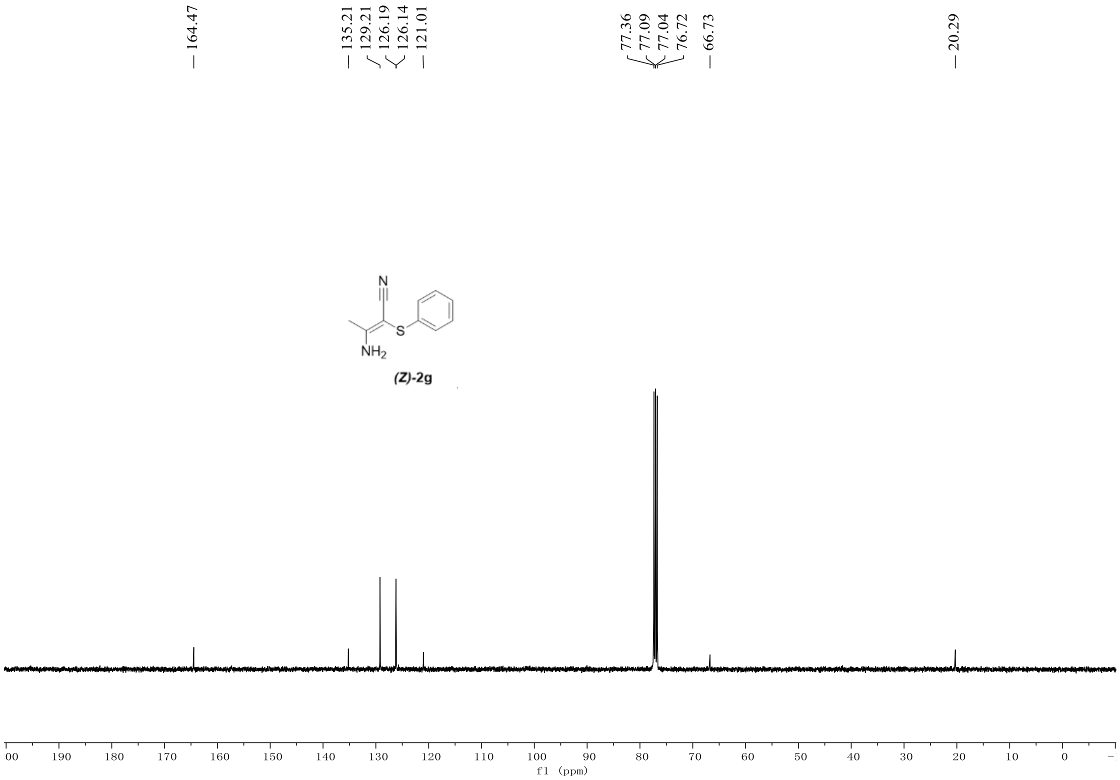

Supplementary Figure 41. ^{13}C NMR spectrum of *(Z)*-2g

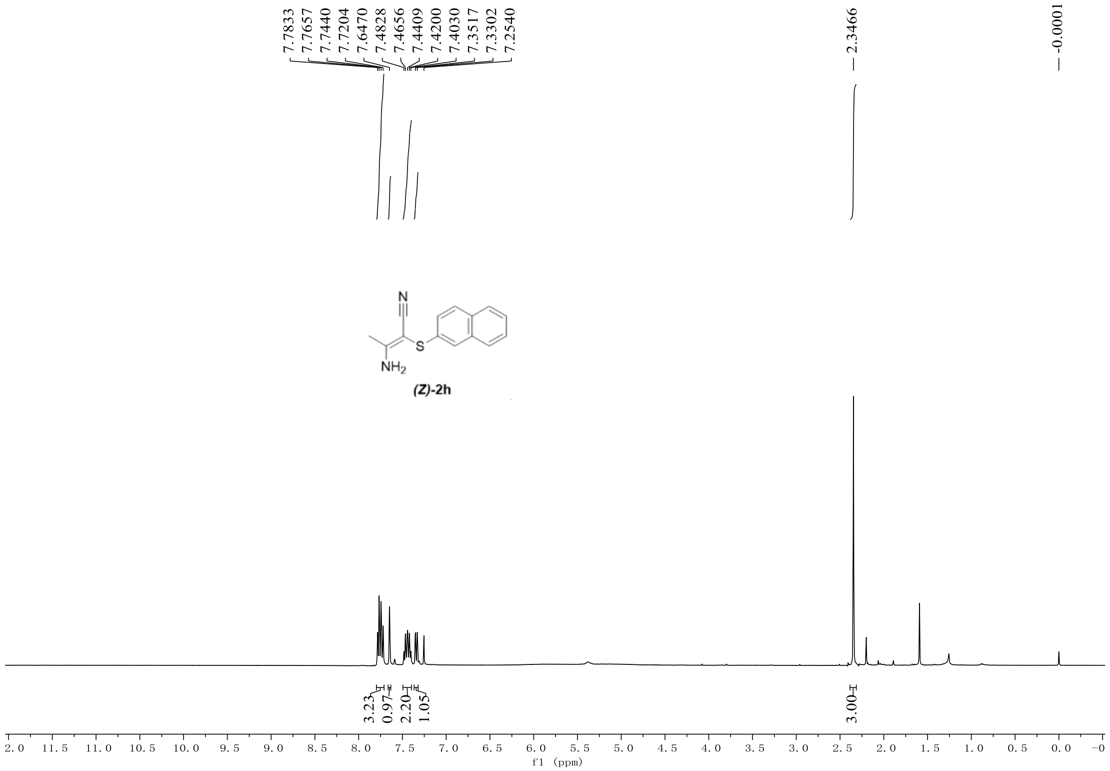

Supplementary Figure 42. ^{1}H NMR spectrum of *(Z)*-2h

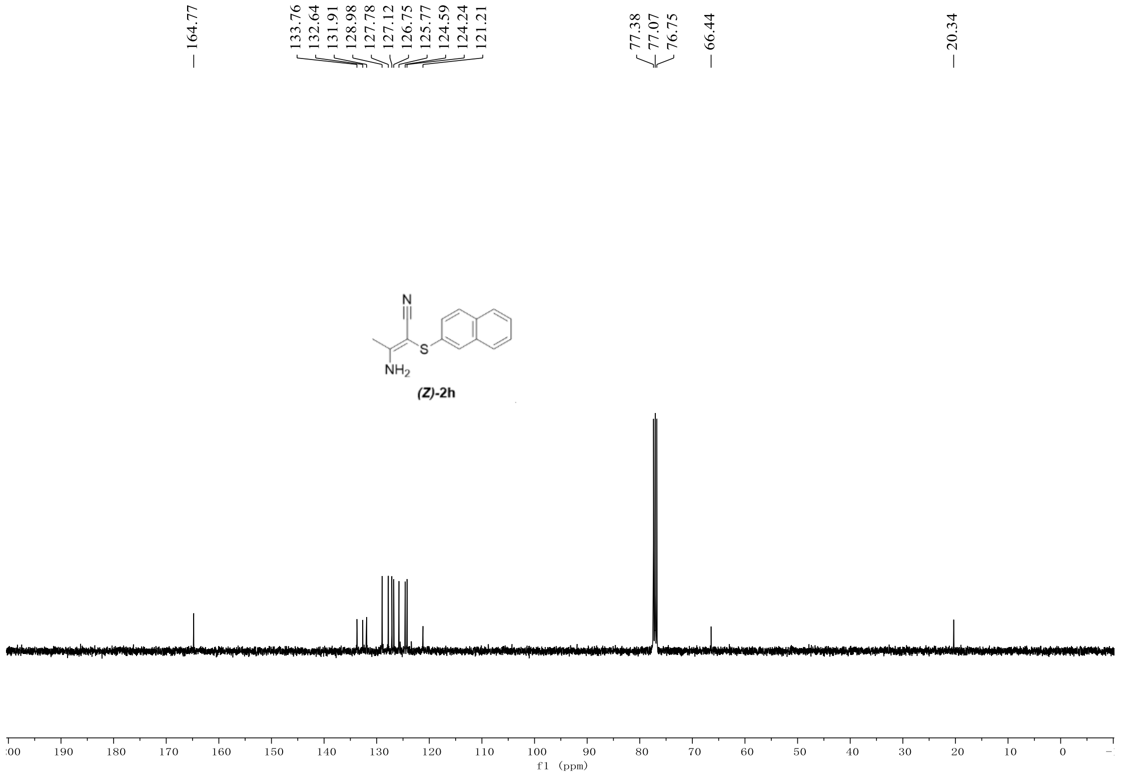

Supplementary Figure 43. ^{13}C NMR spectrum of *(Z)*-2h

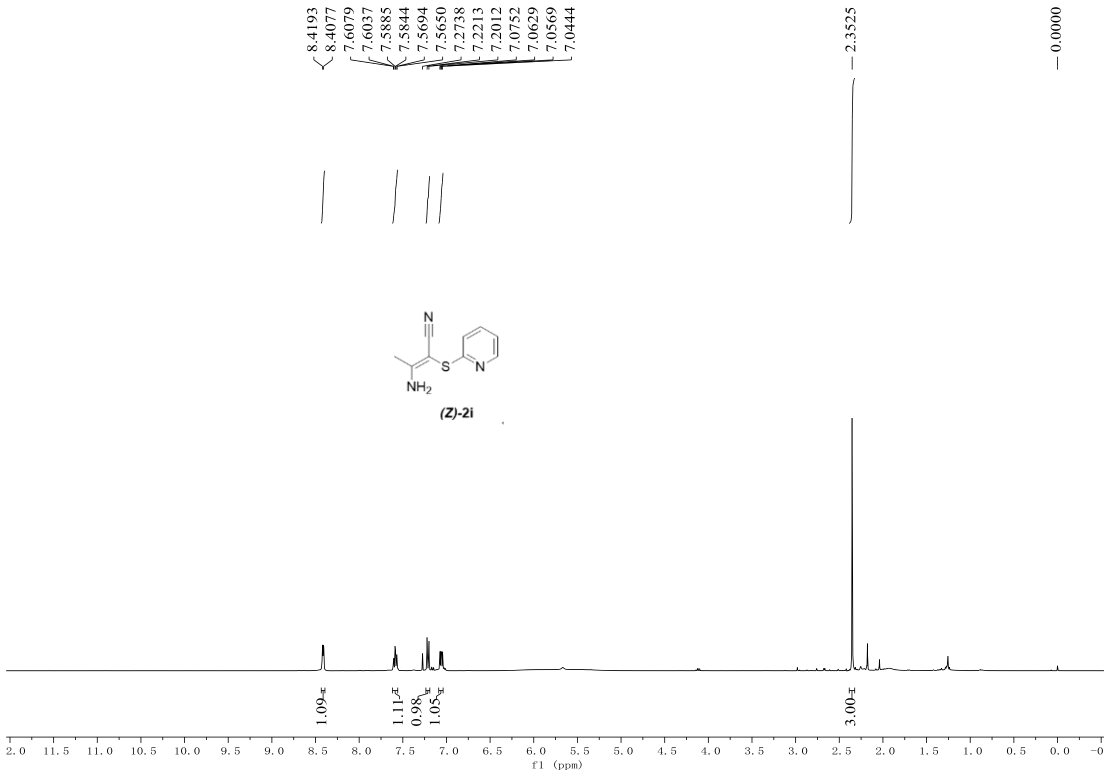

Supplementary Figure 44. ^{1}H NMR spectrum of *(Z)*-2i

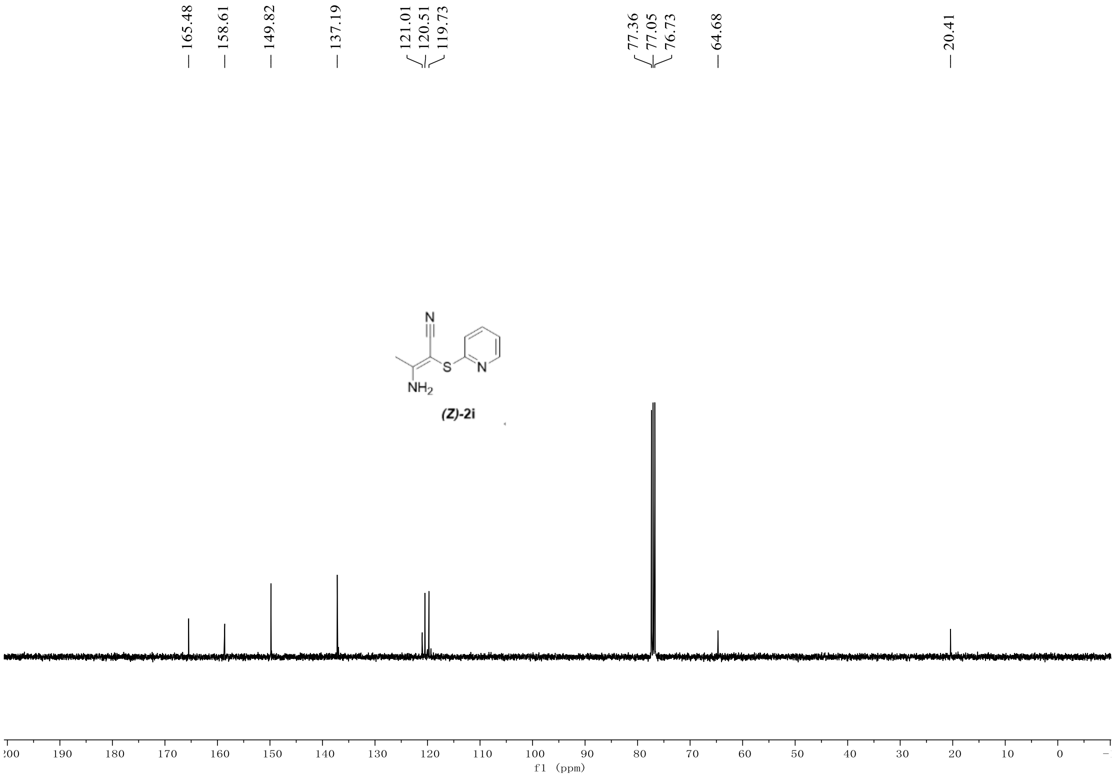

Supplementary Figure 45. ^{13}C NMR spectrum of *(Z)*-2i

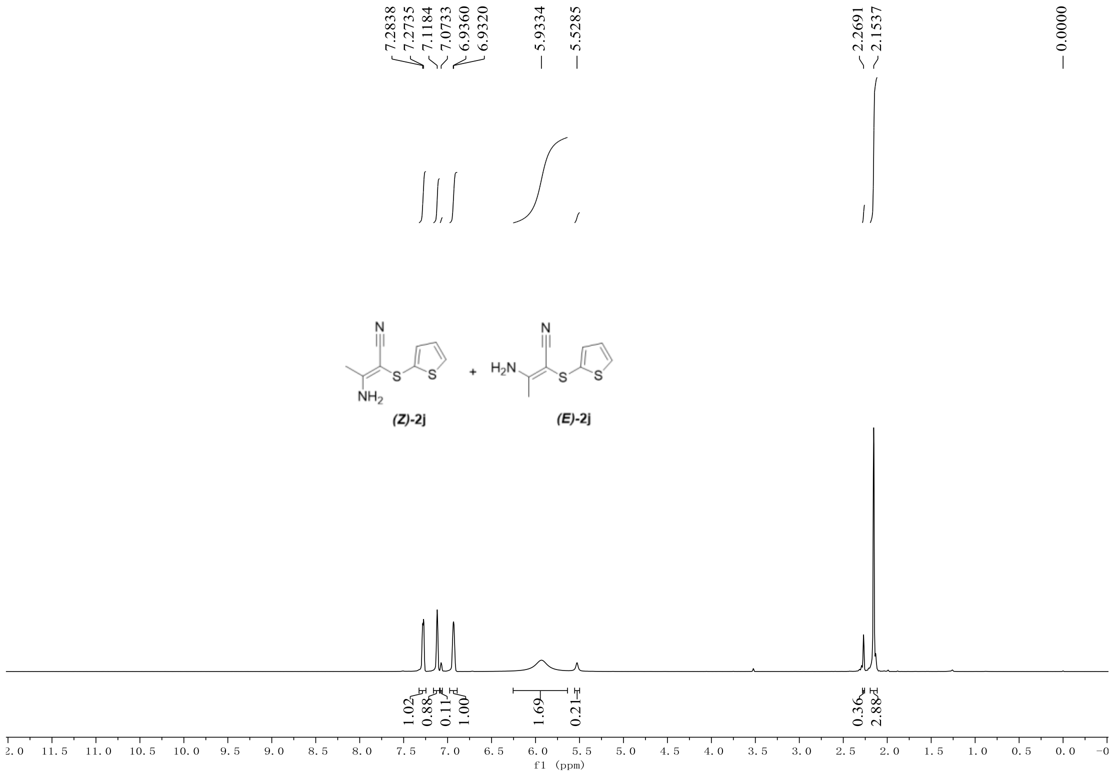

Supplementary Figure 46. ^{1}H NMR spectrum of 2j

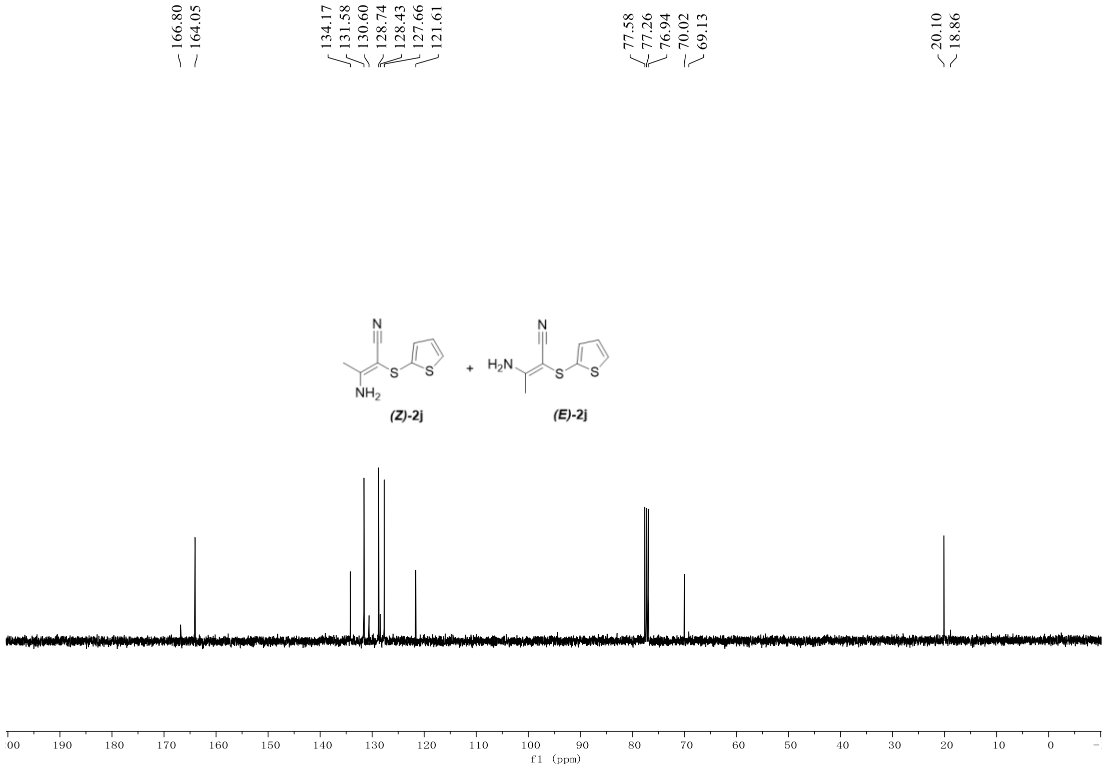

Supplementary Figure 47. ^{13}C NMR spectrum of 2j

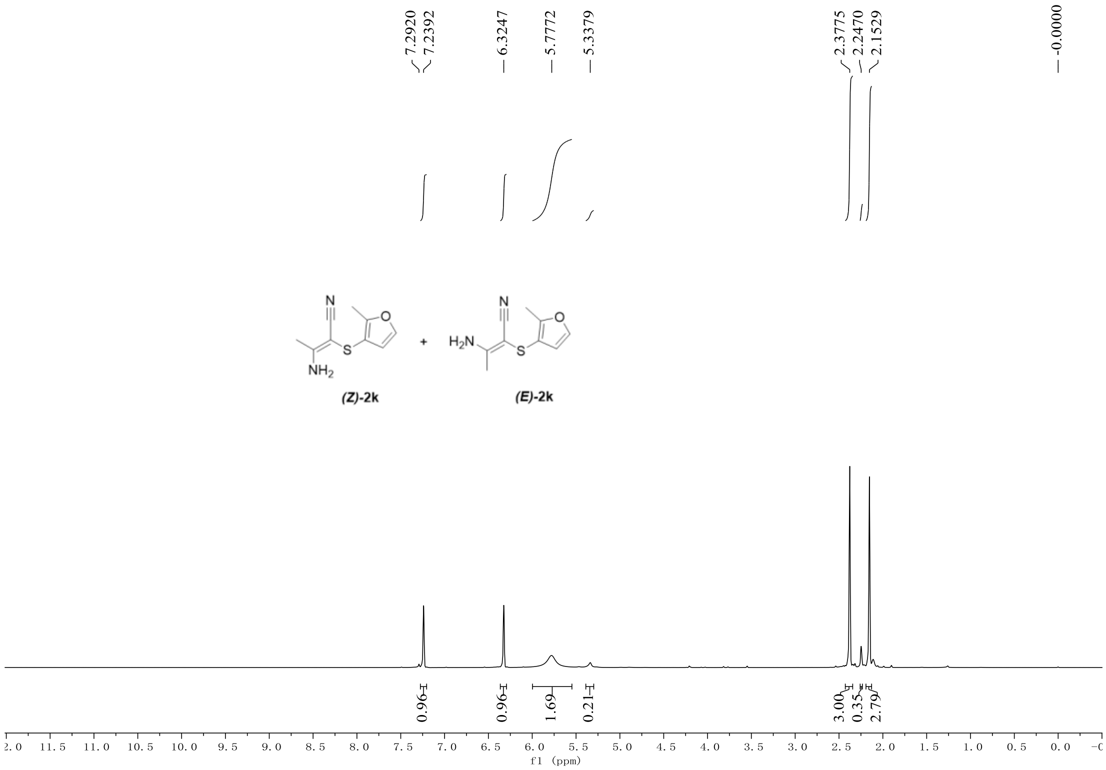

Supplementary Figure 48. ^{1}H NMR spectrum of 2k

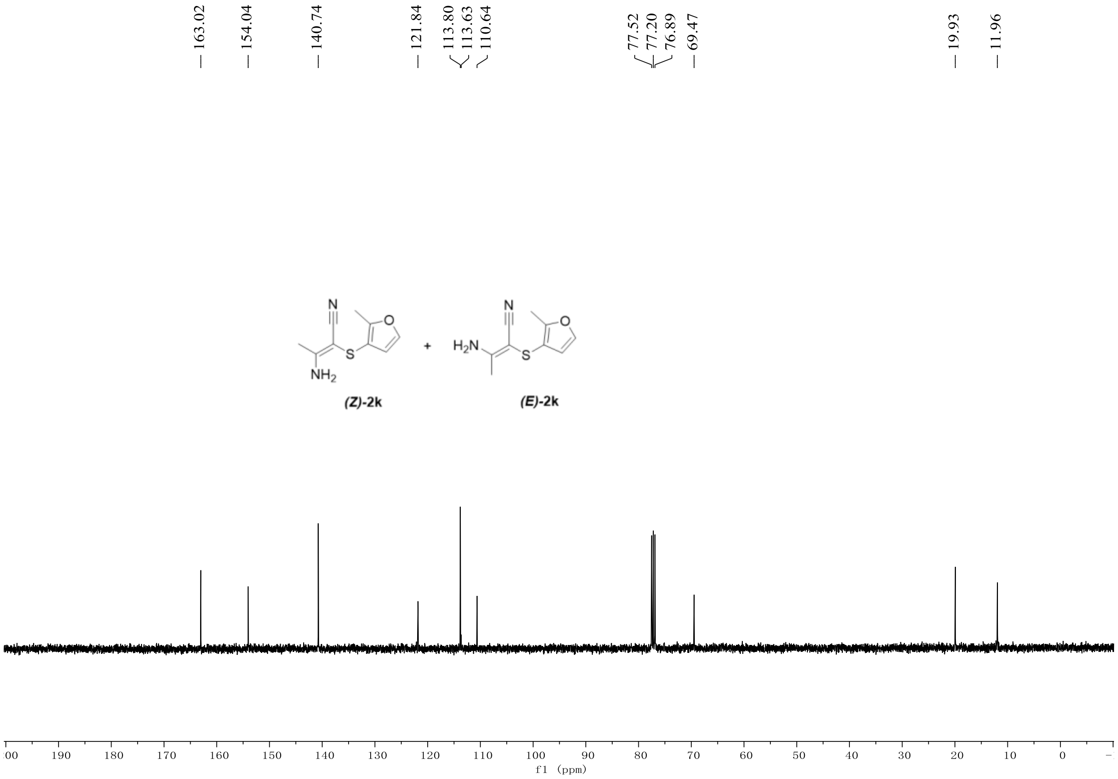

Supplementary Figure 49. ^{13}C NMR spectrum of 2k

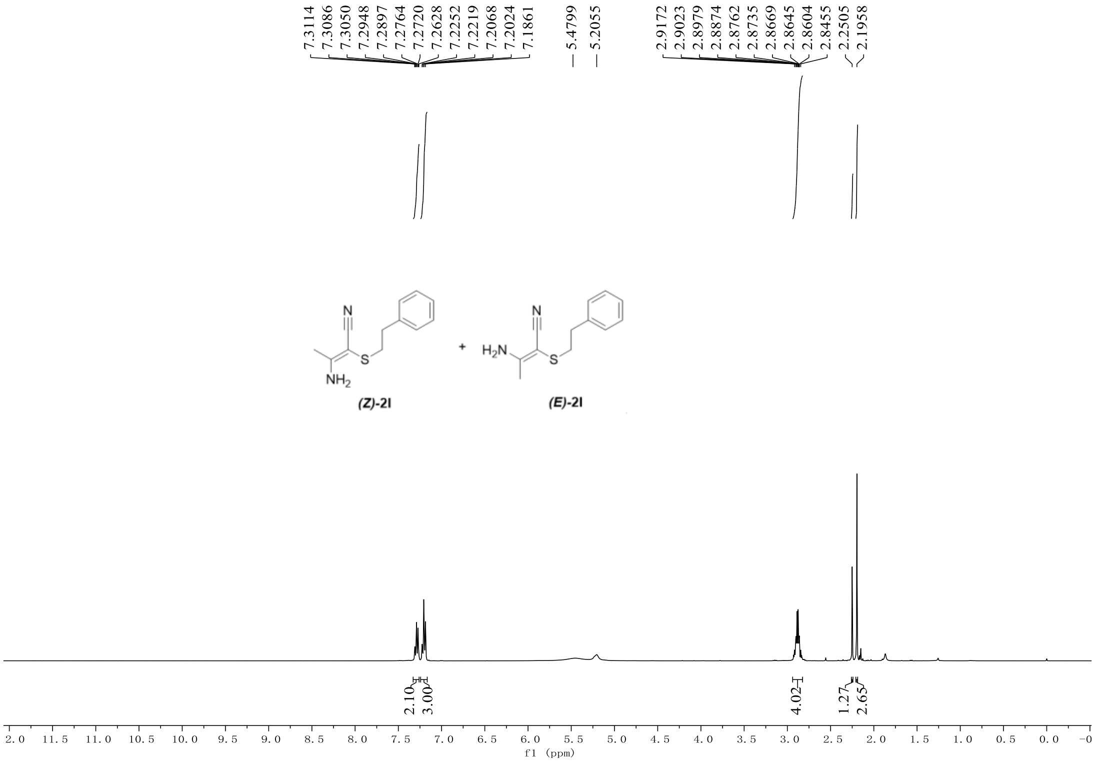

Supplementary Figure 50. ^{1}H NMR spectrum of 2l

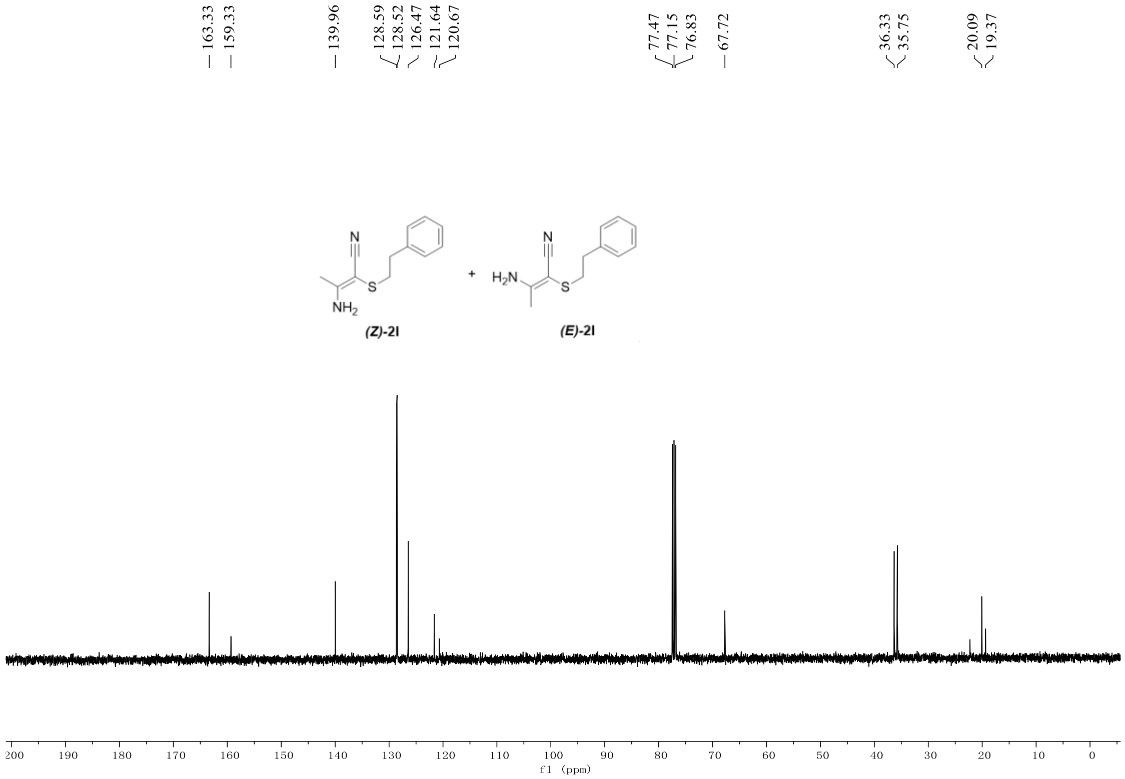

Supplementary Figure 51. ^{13}C NMR spectrum of 2l

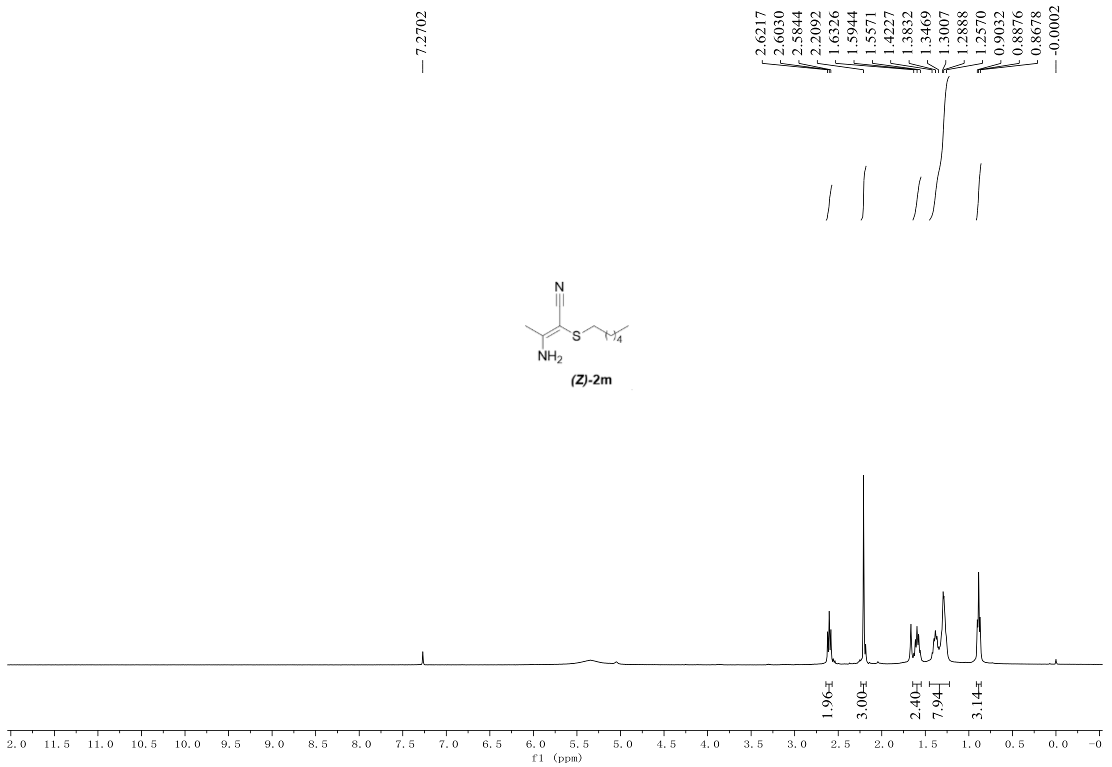

Supplementary Figure 52. ^{1}H NMR spectrum of *(Z)*-2m

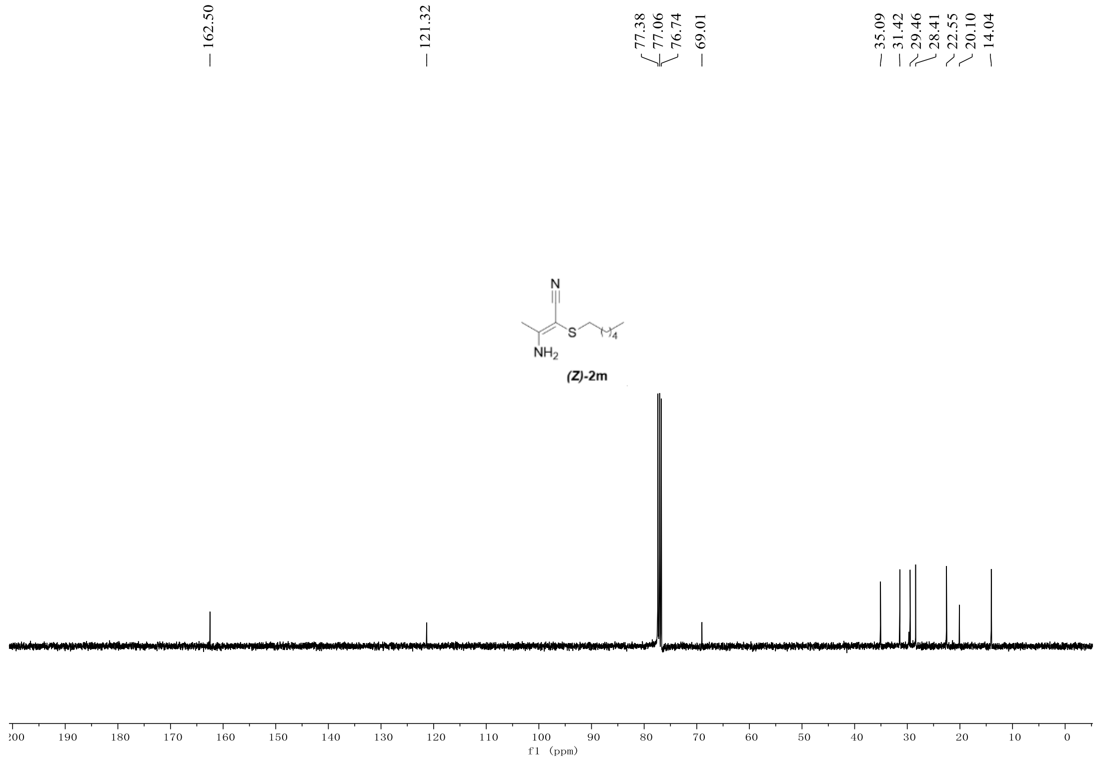

Supplementary Figure 53. ^{13}C NMR spectrum of *(Z)*-2m

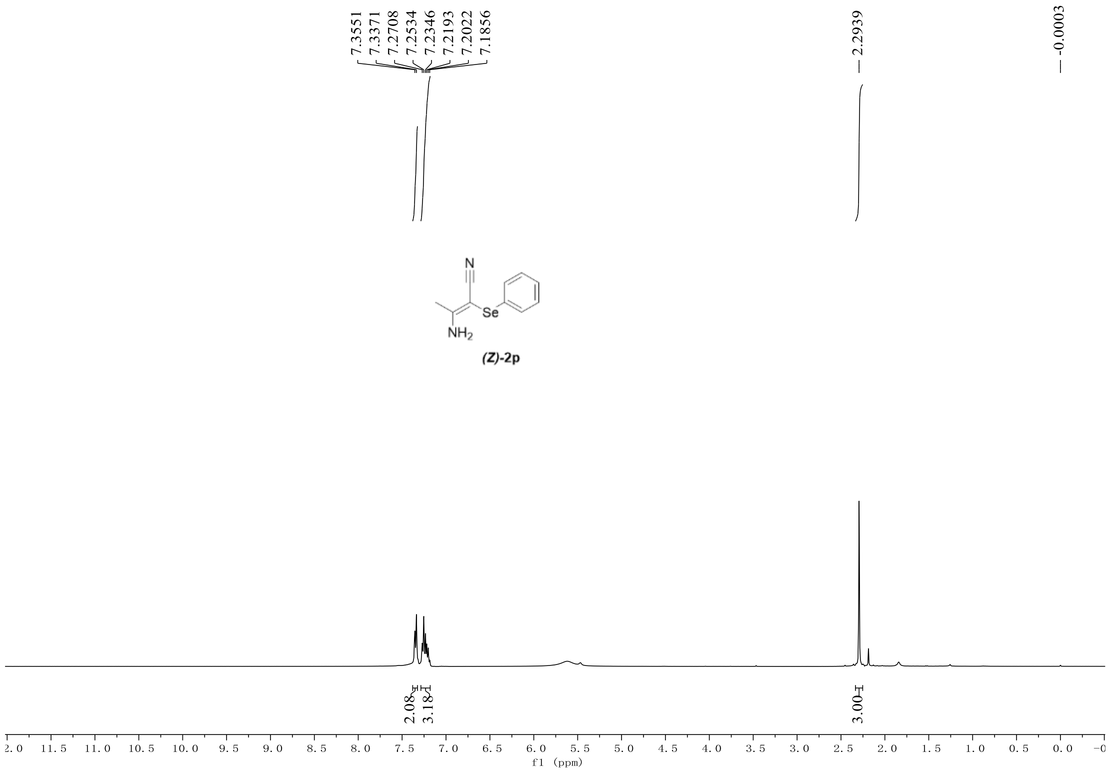

Supplementary Figure 54. ^{1}H NMR spectrum of *(Z)*-2p

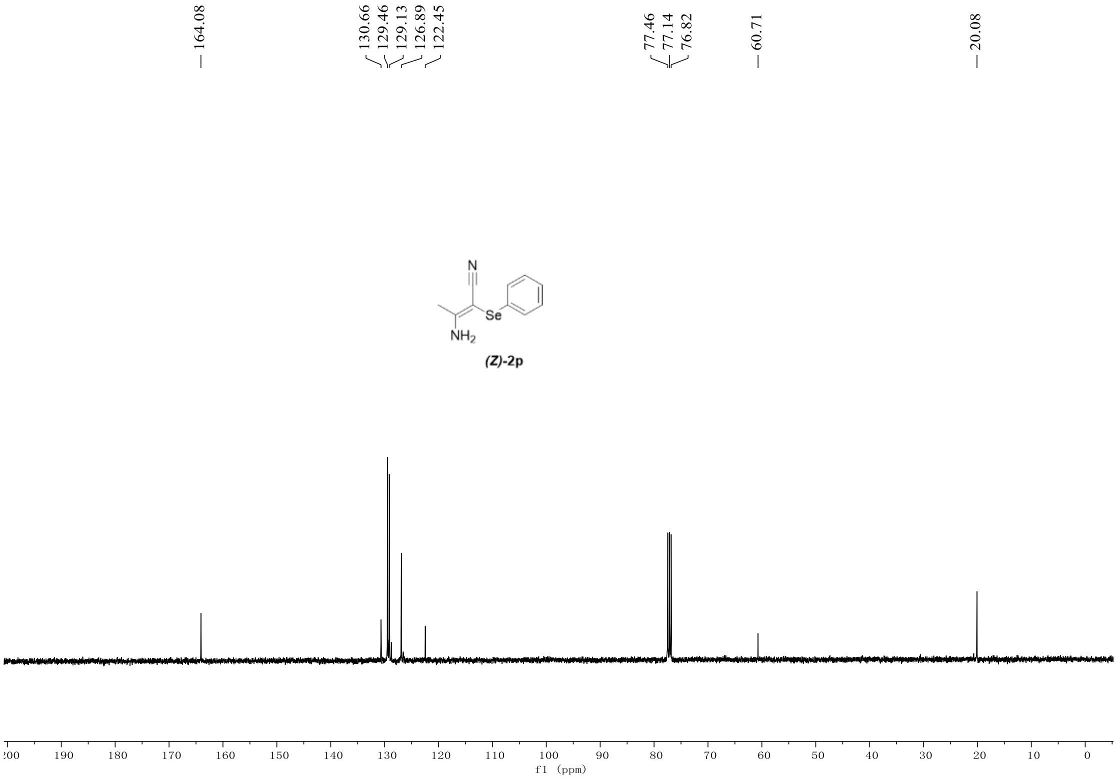

Supplementary Figure 55. ^{13}**C NMR spectrum of** ***(Z)*****-2p**

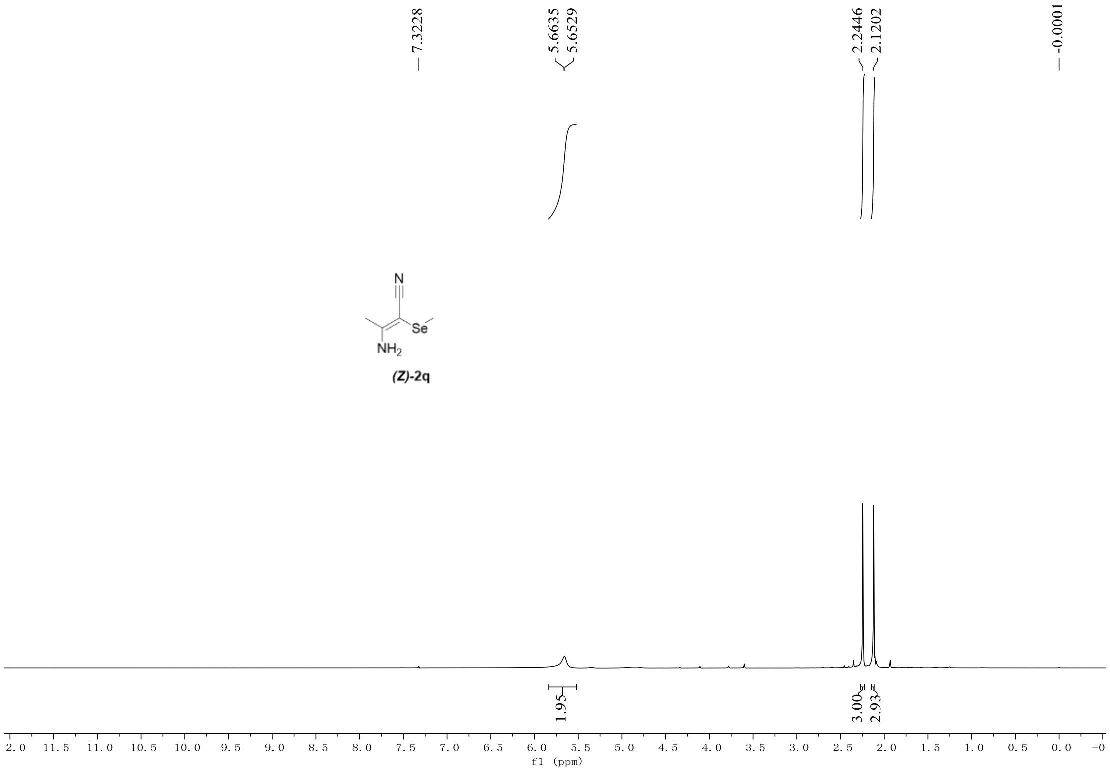

Supplementary Figure 56. ^{1}**H NMR spectrum of** ***(Z)*****-2q**

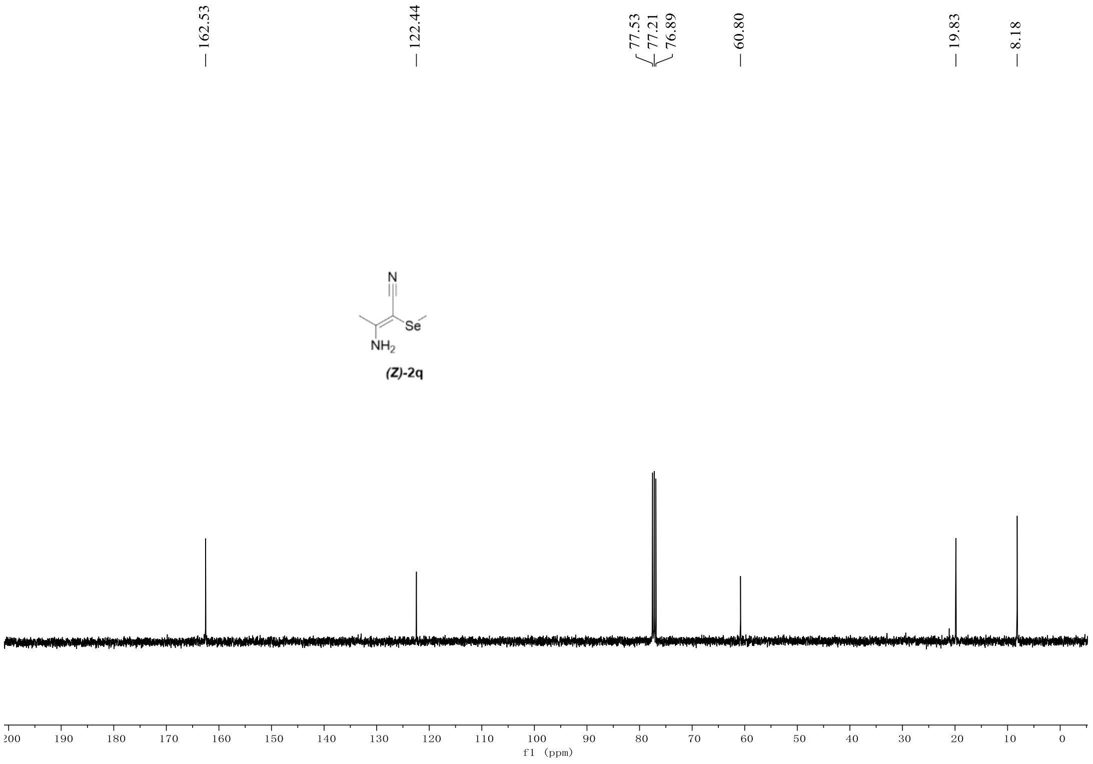

Supplementary Figure 57. ^{13}C NMR spectrum of *(Z)*-2q

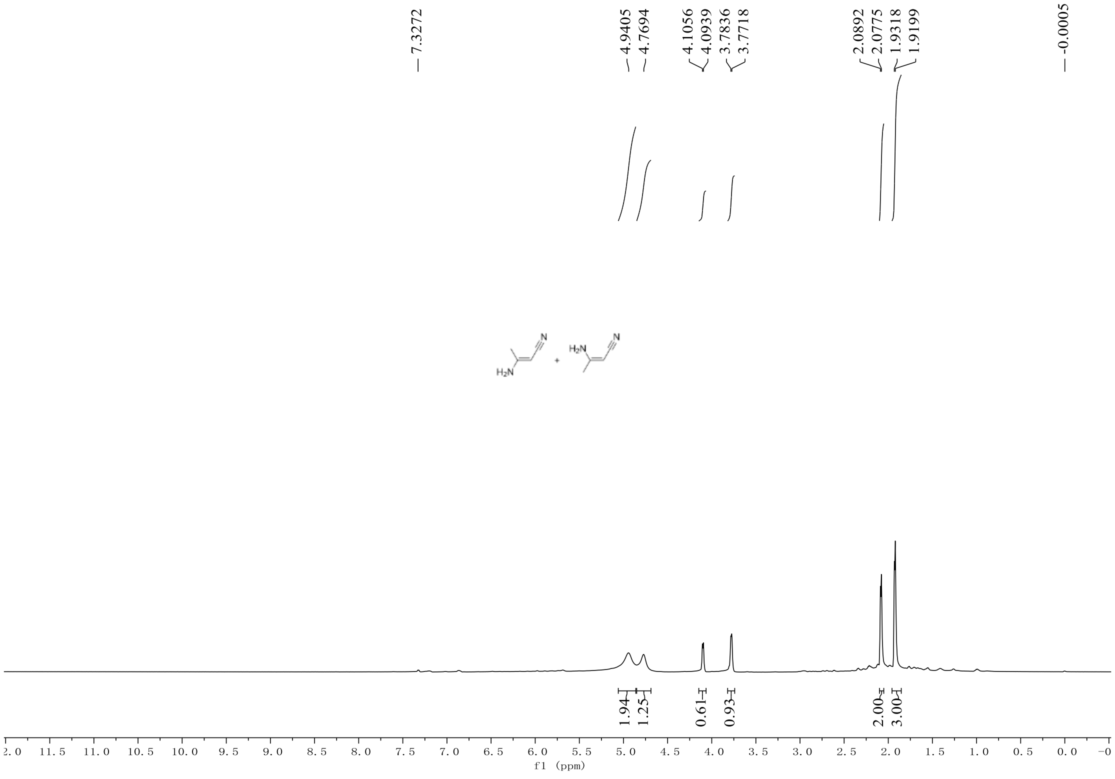

Supplementary Figure 58. ^{1}H NMR spectrum of 3-Aminocrotononitrile

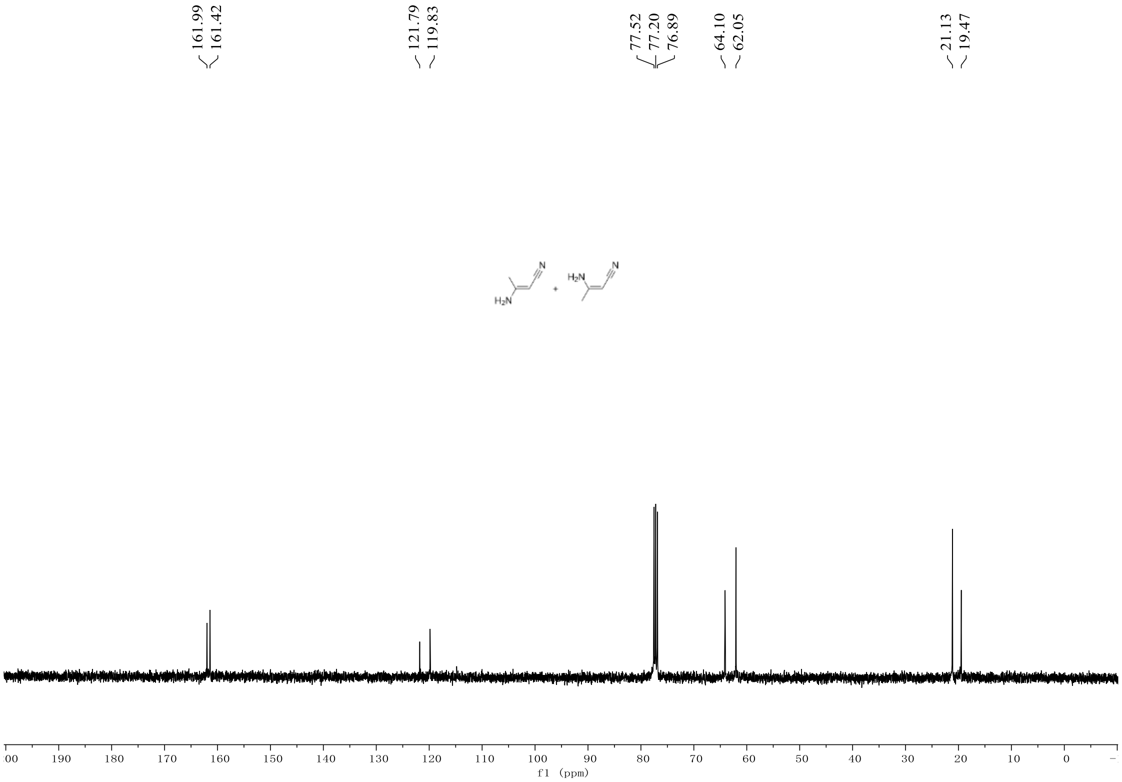

Supplementary Figure 59. ^{13}C NMR spectrum of 3-Aminocrotononitrile

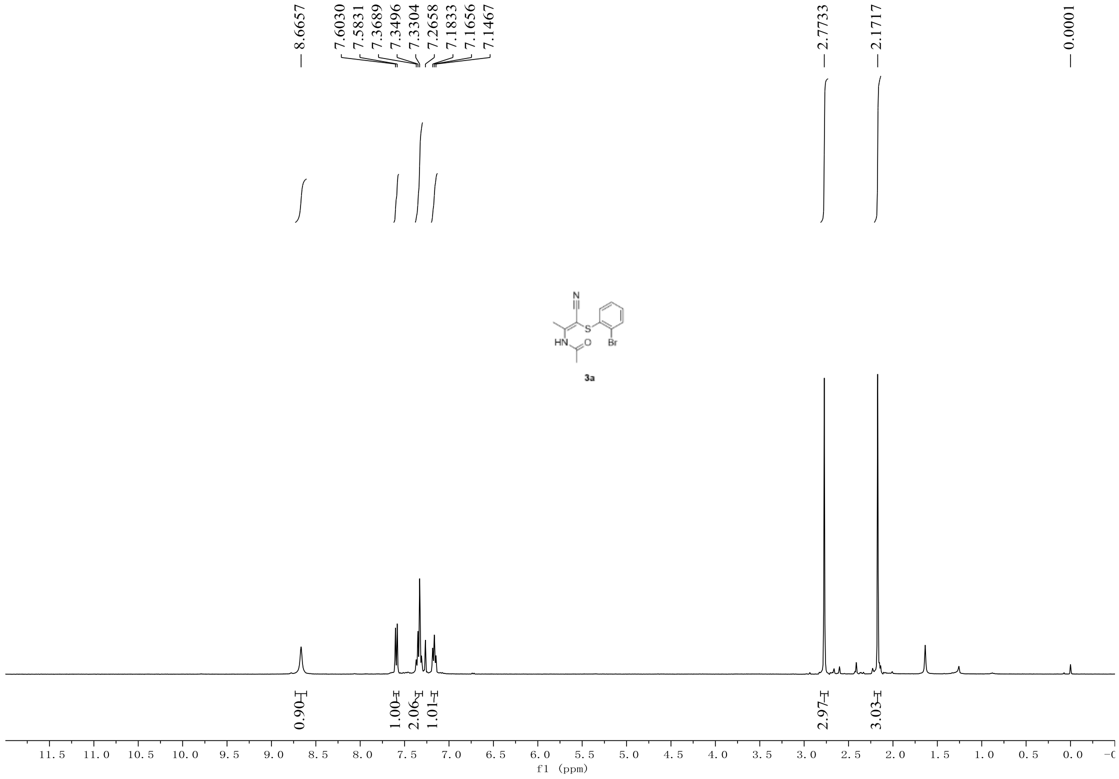

Supplementary Figure 60. ^{1}H NMR spectrum of 3a

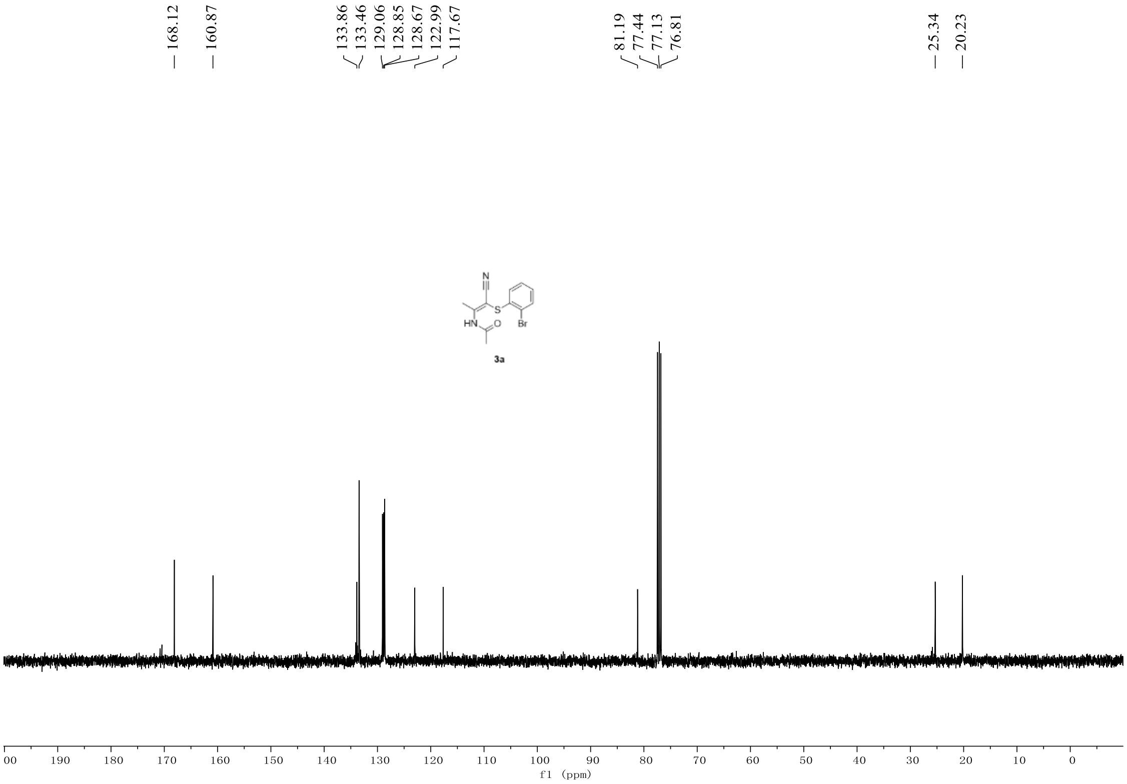

Supplementary Figure 61. ^{13}C NMR spectrum of 3a

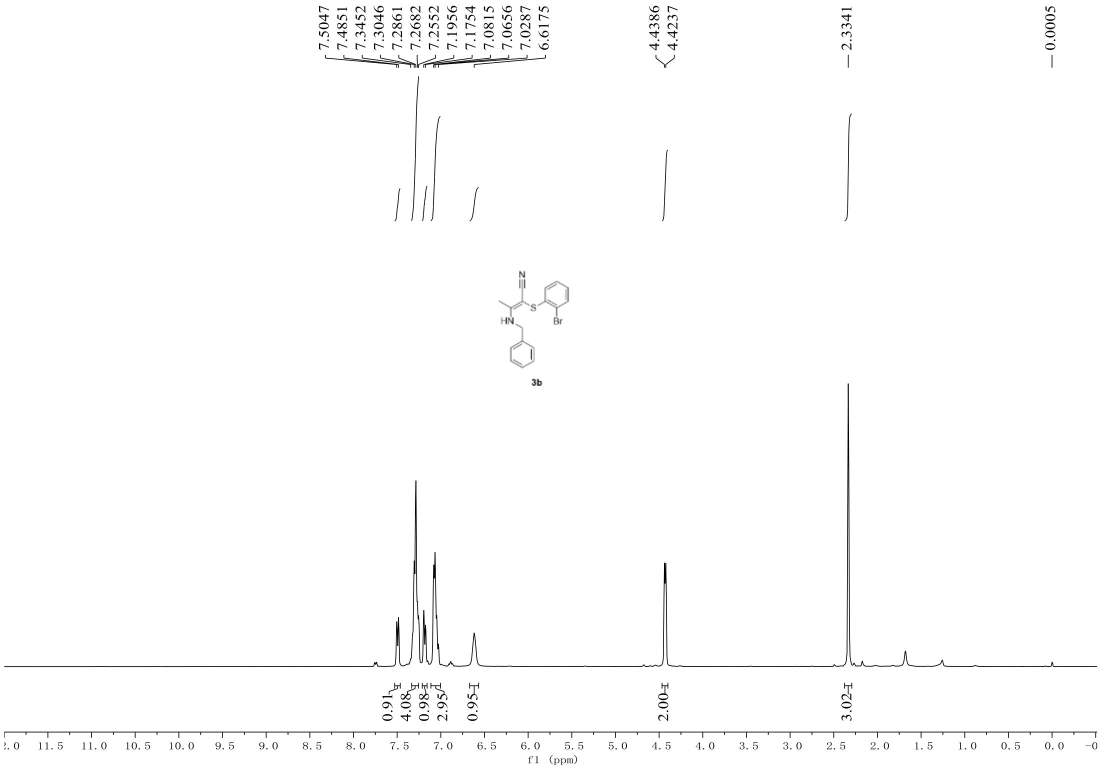

Supplementary Figure 62. ^{1}H NMR spectrum of 3b

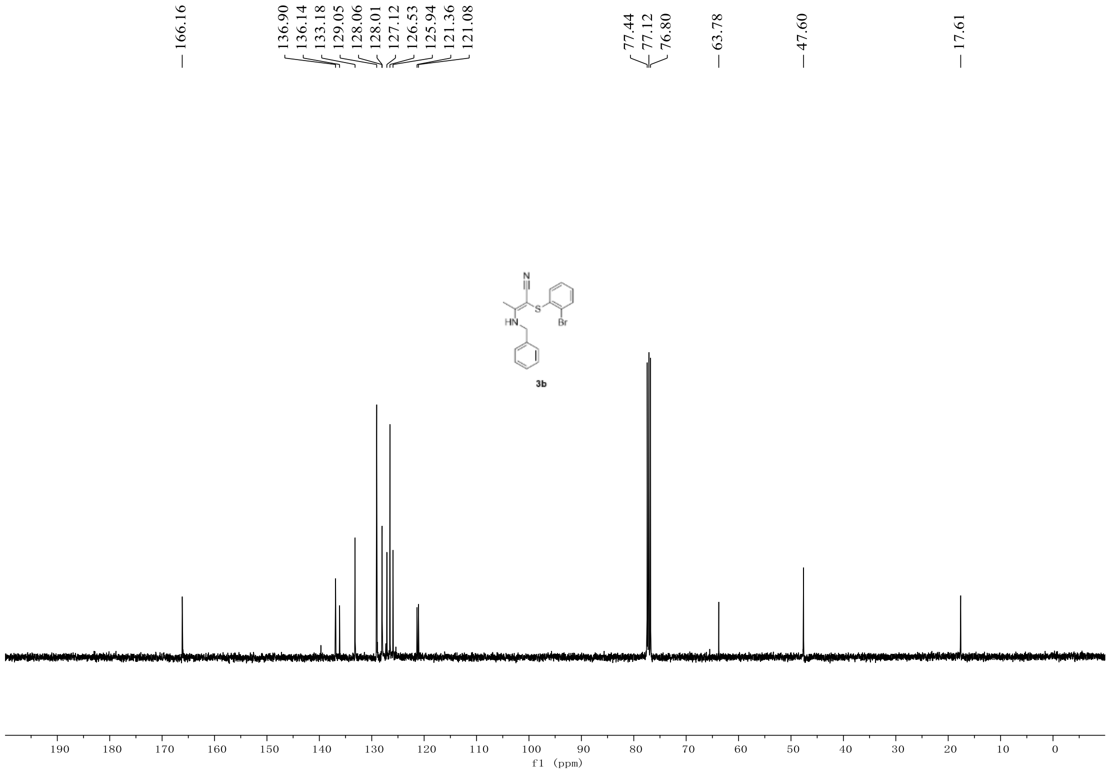

Supplementary Figure 63. ^{13}C NMR spectrum of 3b

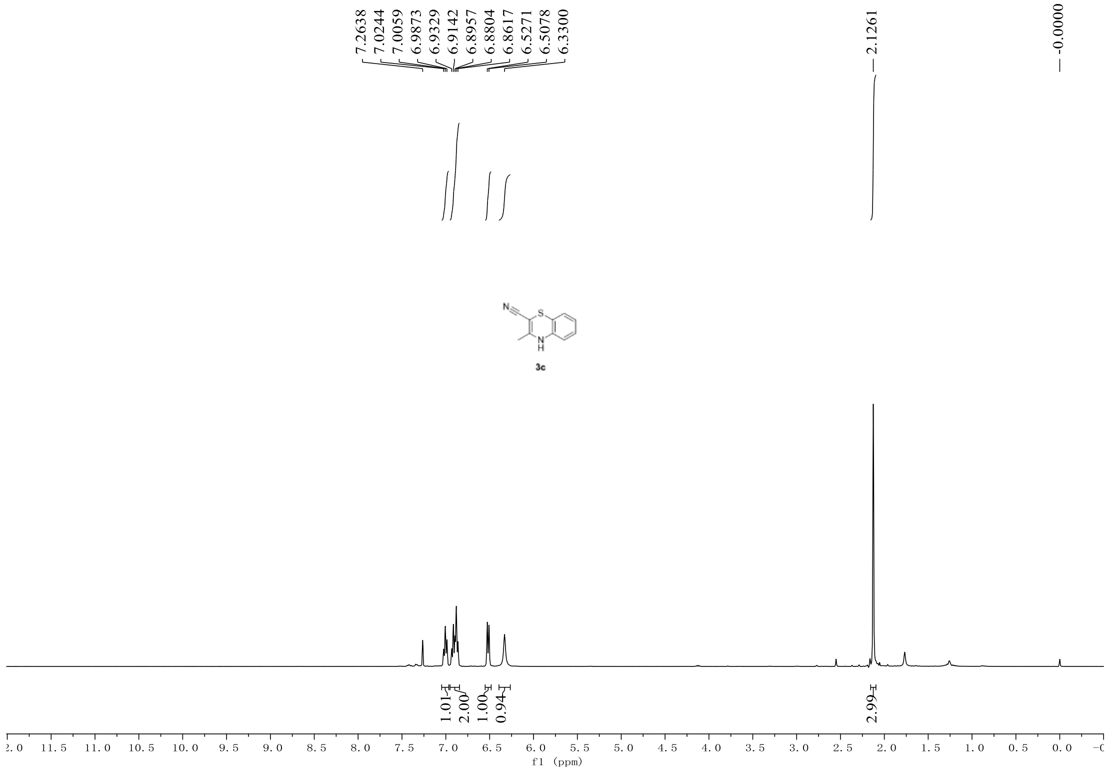

Supplementary Figure 64. ^{1}H NMR spectrum of 3c

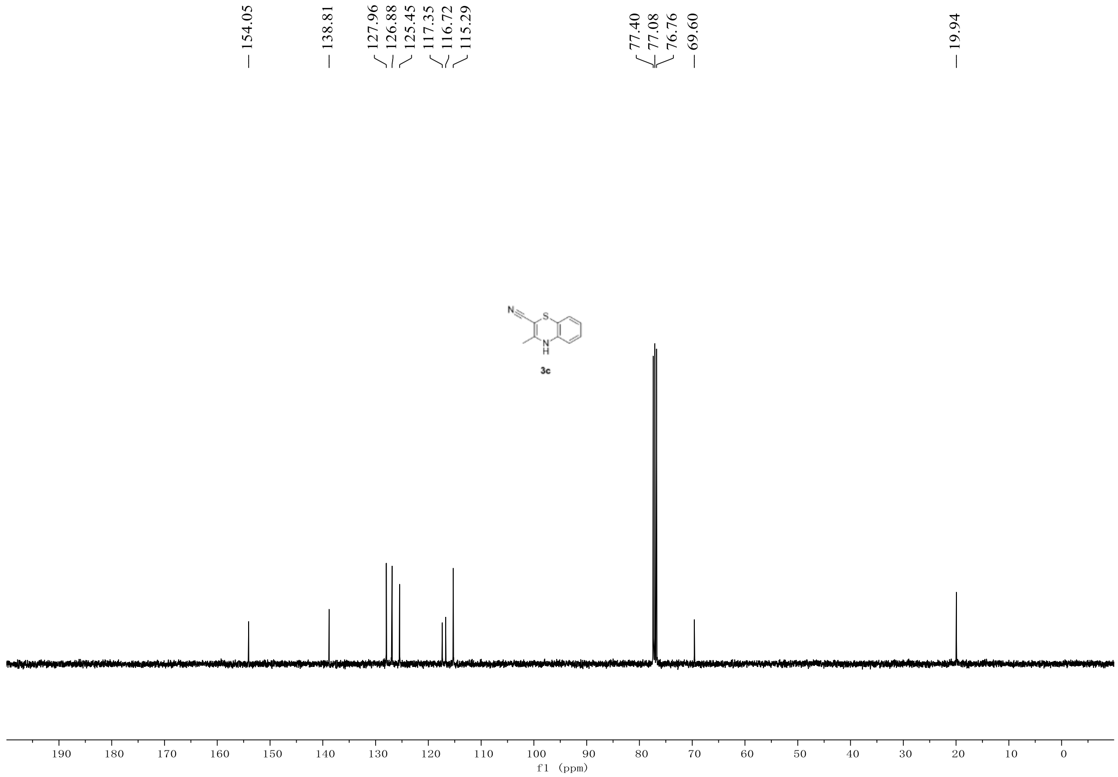

Supplementary Figure 65. ^{13}C NMR spectrum of 3c

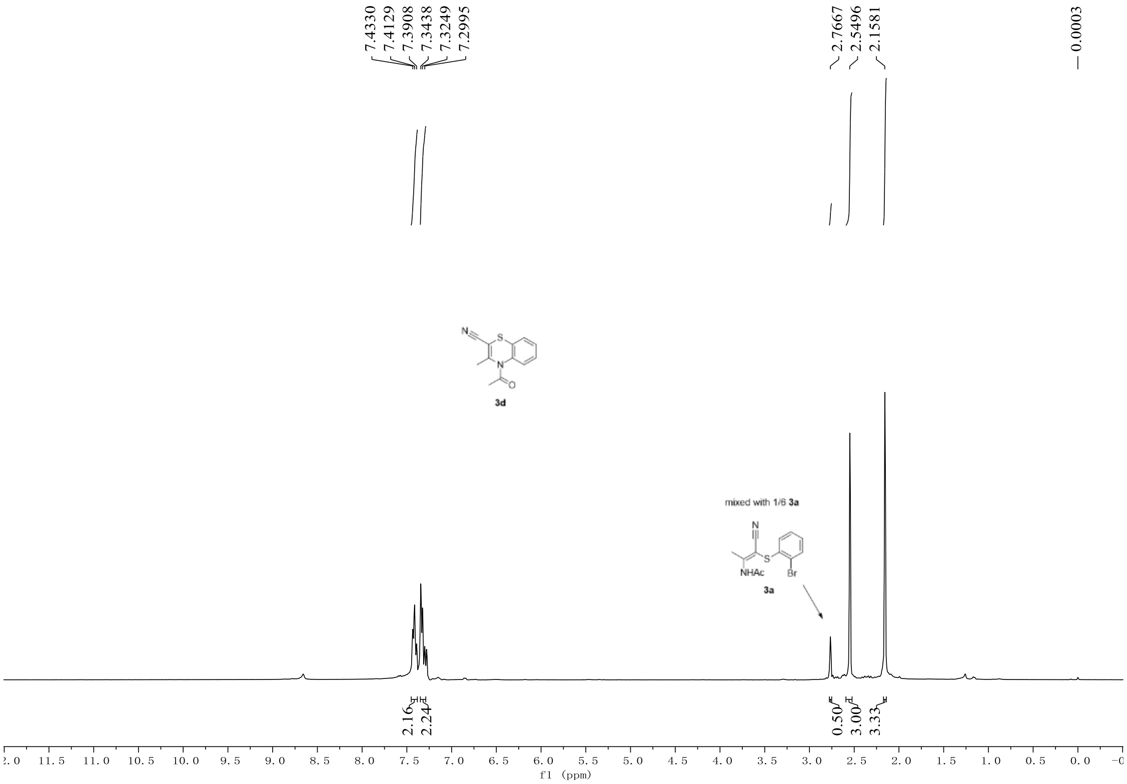

Supplementary Figure 66. ^{1}H NMR spectrum of 3d

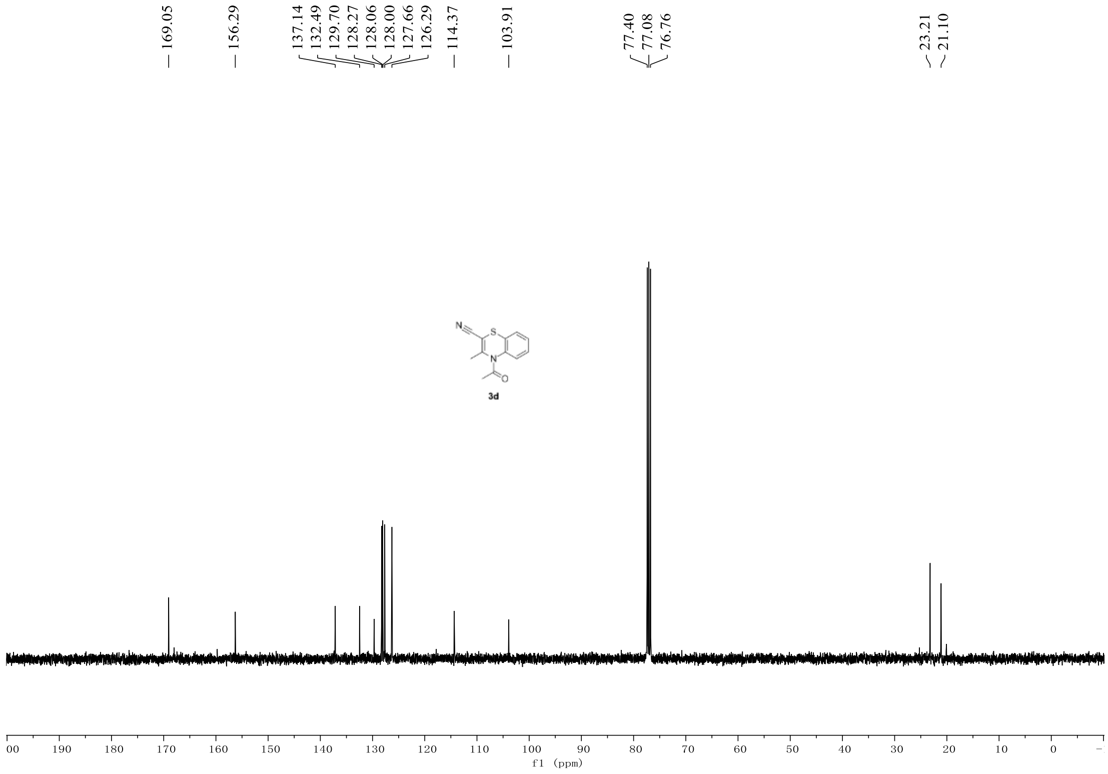

Supplementary Figure 67. ^{13}C NMR spectrum of 3d

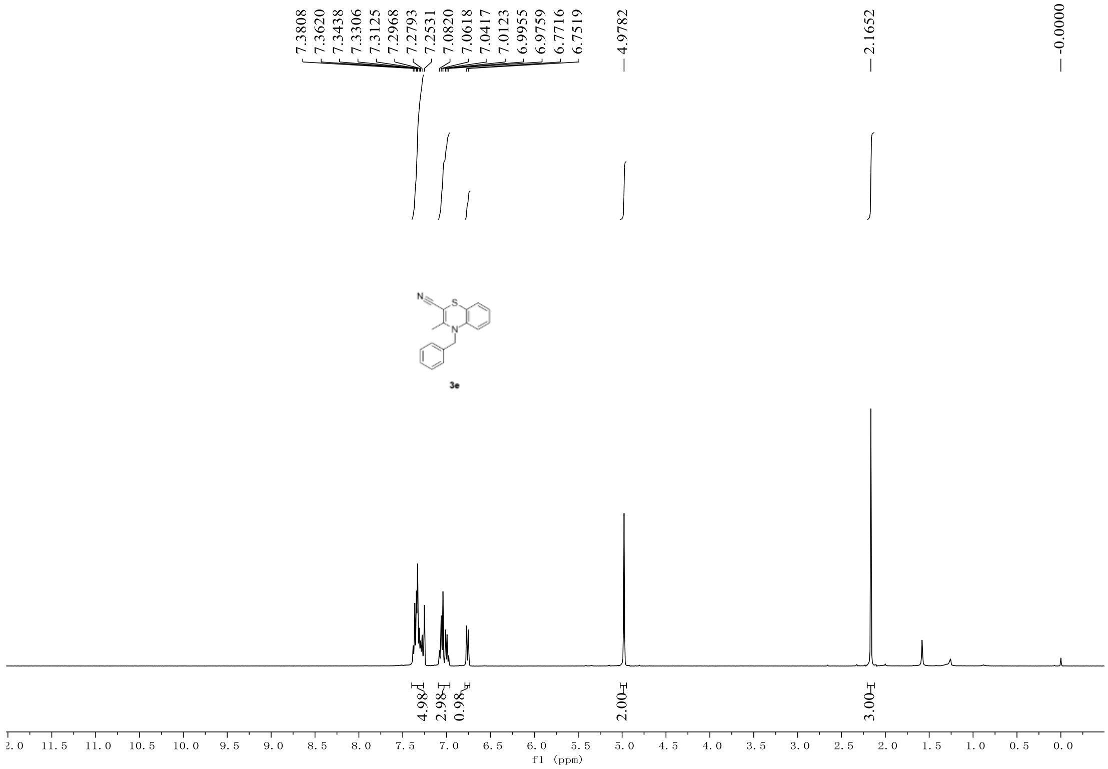

Supplementary Figure 68. ^{1}H NMR spectrum of 3e

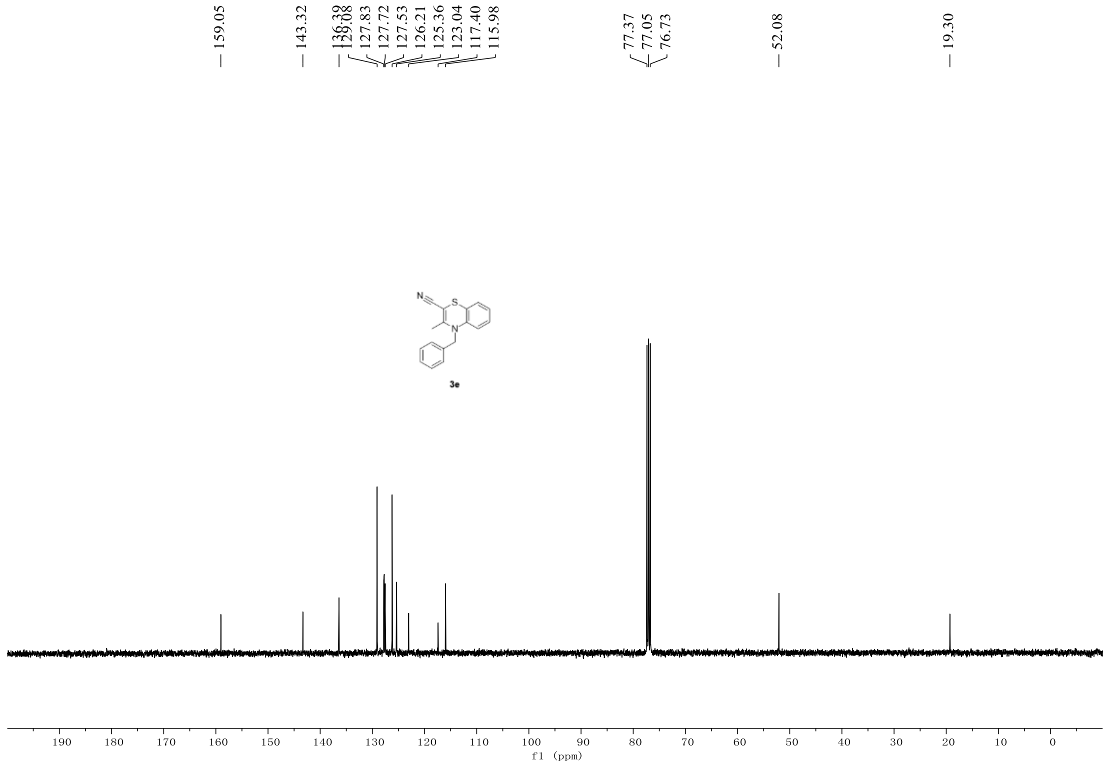

Supplementary Figure 69. ^{13}C NMR spectrum of 3e

Supplementary References

1. J. M. John, R. Loorthuraja, E. Antoniuk, S. H. Bergens. Catalytic hydrogenation of functionalized amides under basic and neutral conditions. *Catal. Sci. Technol.*, **5**, 1181–1186 (2015).
2. R. Masciadri, M. Kamer, N. Nock. Regioselective Friedel−Crafts Alkylation of Anilines and Amino-Substituted Heteroarenes with Hexafluoroacetone Sesquihydrate. *Eur. J. Org. Chem.*, **21**, 4286–4291 (2003).
3. R. A. Altman, A. M. Hyde, X. h. Huang, S. L. Buchwald. Orthogonal Pd- and Cu-Based Catalyst Systems for C- and N-Arylation of Oxindoles. *J. Am. Chem. Soc.*, **130**, 9613–9620 (2008).
4. L. A. Farmer, E. A. Haidasz, M. Griesser, D. A. Pratt. Phenoxazine: A Privileged Scaffold for Radical-Trapping Antioxidants. *J. Org. Chem.*, **82**, 10523−10536 (2017).
5. Frisch, M. J.; Schlegel, H. B.; Scuseria, G. E.; Robb, M. A.; Cheeseman, J. R.; Scalmani, G.; Barone, V.; Mennucci, B.; Petersson, G. A.; Nakatsuji, H.; Caricato, M.; Li, X.; Hratchian, H. P.; Izmaylov, A. F.; Bloino, J.; Zheng, G.; Sonnenberg, J. L.; Hada, M.; Ehara, M.; Toyota, K.; Fukuda, R.; Hasegawa, J.; Ishida, M.; Nakajima, T.; Honda, Y.; Kitao, O.; Nakai, H.; Vreven, T.; Montgomery, J. A., Jr.; Peralta, J. E.; Ogliaro, F.; Bearpark, M.; Heyd, J. J.; Brothers, E.; Kudin, K. N.; Staroverov, V. N.; Keith, T.; Kobayashi, R.; Normand, J.; Raghavachari, K.; Rendell, A.; Burant, J. C.; Iyengar, S. S.; Tomasi, J.; Cossi, M.; Rega, N.; Millam, J. M.; Klene, M.; Knox, J. E.; Cross, J. B.; Bakken, V.; Adamo, C.; Jaramillo, J.; Gomperts, R.; Stratmann, R. E.; Austin, A. J.; Cammi, R.; Pomelli, C.; Ochterski, J. W.; Martin, R. L.; Morokuma, K.; Zakrzewski, V. G.; Voth, G. A.; Salvador, P.; Dannenberg, J. J. D. S.; Daniels, A. D.; Farkas, O.; Foresman, J. B.; Ortiz, J. V.; Cioslowski, J.; Fox, D. J. *Gaussian 09, Revision D.01* (Gaussian, Inc., Wallingford CT, 2009).
6. Zhao, Y.; Truhlar, D. G. The M06 suite of density functionals for main group thermochemistry, thermochemical kinetics, noncovalent interactions, excited states, and transition elements: two new functionals and systematic testing of four M06-class functionals and 12 other functionals. *Theor. Chem. Acc.*, **120**, 215−241 (2008).
7. Marenich, A. V., Cramer, C. J.; Truhlar, D. G. Universal Solvation Model Based on Solute Electron Density and on a Continuum Model of the Solvent Defined by the Bulk Dielectric Constant and Atomic Surface Tensions. *The Journal of Physical Chemistry B.*, **113**, 6378−6396 (2009).
8. Winikoff, S. G.; Cramer, C. J. Mechanistic analysis of water oxidation catalyzed by mononuclear copper in aqueous bicarbonate solutions. *Catal. Sci. Technol.*, **4**, 2484−2489 (2014).
9. Liang, Y., Liu, S., Xia, Y., Li, Y.; Yu, Z. X. Mechanism, regioselectivity, and the kinetics of phosphine-catalyzed [3+2] cycloaddition reactions of allenoates and electron-deficient alkenes. *Chem. Eur. J.*, **14**, 4361−4373 (2008).
10. Li, H., Wen, M.; Wang, Z. X. Computational mechanistic study of the hydrogenation of carbonate to methanol catalyzed by the Ru(II)PNN complex. *Inorg. Chem.*, **51**, 5716−5727 (2012).
