# Supplementary material for: Stereoselective synthesis of sulfur-containing β-enaminonitrile derivatives through electrochemical Csp3–H bond oxidative functionalization of acetonitrile
Source: Nat Commun. 2019 Feb 19;10:833. doi: 10.1038/s41467-019-08762-5 (PMC6381189; doi:10.1038/s41467-019-08762-5)
Supplement: Supplementary file 3 — Description of Additional Supplementary Files [file 41467_2019_8762_MOESM3_ESM.pdf]

Description of Additional Supplementary Files

File Name: Supplementary Dataset 1

Description: Cartesian coordinates for all optimized structures
