# Supplementary material for: Stereoselective synthesis of sulfur-containing β-enaminonitrile derivatives through electrochemical Csp3–H bond oxidative functionalization of acetonitrile
Source: Nat Commun. 2019 Feb 19;10:833. doi: 10.1038/s41467-019-08762-5 (PMC6381189; doi:10.1038/s41467-019-08762-5)
Supplement: Supplementary file 4 — Supplementary Data 1 [file 41467_2019_8762_MOESM4_ESM.doc]

**The Cartesian coordinates (xyz) for all optimized structures are presented**

DPPE-oxide


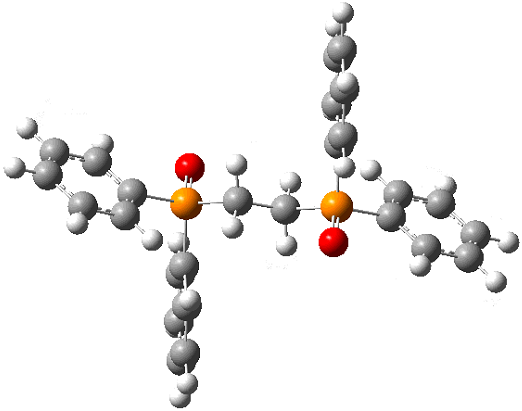


0 1

P 2.075192 0.086036 0.748937

C 3.438518 -0.973470 0.174548

C 4.284958 -1.513626 1.144376

C 5.348389 -2.328593 0.765524

C 5.565563 -2.608265 -0.580495

C 4.719765 -2.073926 -1.550608

C 3.656867 -1.258202 -1.177039

C 2.443284 1.785714 0.208915

C 2.657375 2.750542 1.193683

C 2.932922 4.066736 0.830484

C 2.991387 4.420122 -0.514061

C 2.772556 3.458866 -1.499932

C 2.497727 2.143737 -1.141603

C 0.607431 -0.419430 -0.199360

H 4.103522 -1.296152 2.191337

H 6.004407 -2.746206 1.520767

H 6.393163 -3.243713 -0.875139

H 4.886204 -2.294935 -2.598721

H 3.003420 -0.850365 -1.940909

H 2.602613 2.466716 2.239134

H 3.097831 4.814894 1.597578

H 3.205375 5.445051 -0.795984

H 2.813036 3.734583 -2.547595

H 2.323486 1.403244 -1.915728

H 0.451035 -1.481234 0.019066

H 0.820120 -0.322284 -1.267558

C -0.607431 0.419430 0.199360

P -2.075192 -0.086036 -0.748937

H -0.451035 1.481234 -0.019066

H -0.820120 0.322284 1.267558

O -1.893965 -0.006816 -2.240401

C -3.438518 0.973470 -0.174548

C -2.443284 -1.785714 -0.208915

C -4.284958 1.513626 -1.144376

C -3.656867 1.258202 1.177039

C -2.657375 -2.750542 -1.193683

C -2.497727 -2.143737 1.141603

C -5.348389 2.328593 -0.765524

H -4.103522 1.296152 -2.191337

C -4.719765 2.073926 1.550608

H -3.003420 0.850365 1.940909

C -2.932922 -4.066736 -0.830484

H -2.602613 -2.466716 -2.239134

C -2.772556 -3.458866 1.499932

H -2.323486 -1.403244 1.915728

C -5.565563 2.608265 0.580495

H -6.004407 2.746206 -1.520767

H -4.886204 2.294935 2.598721

C -2.991387 -4.420122 0.514061

H -3.097831 -4.814894 -1.597578

H -2.813035 -3.734583 2.547595

H -6.393163 3.243713 0.875139

H -3.205375 -5.445051 0.795984

O 1.893965 0.006816 2.240401

**9**


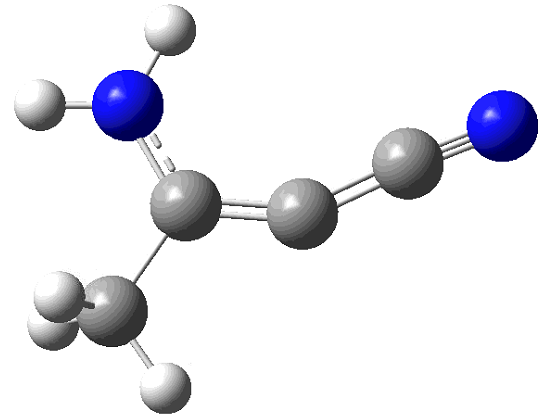


0 2

H 0.455961 2.051871 0.005634

H 2.127273 1.591924 0.011005

N 1.154279 1.323317 -0.004077

C 0.813169 0.019284 0.003068

C -0.455381 -0.420205 0.008265

C 1.953565 -0.960706 -0.002607

H 2.539940 -0.825975 -0.914948

H 2.602830 -0.767872 0.854767

H 1.588361 -1.984708 0.042656

C -1.779323 -0.165637 0.001301

N -2.940929 -0.023554 -0.004390

**9'**


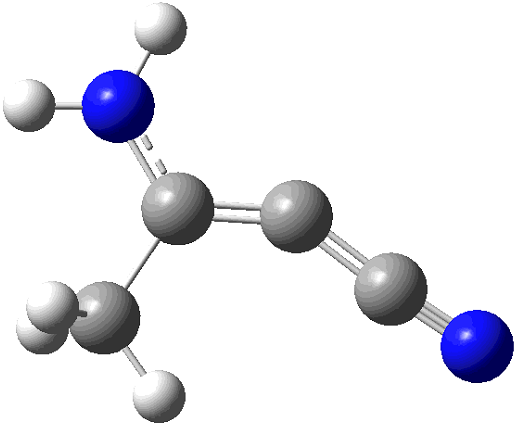


0 2

H 1.785171 -1.924541 0.000742

H 2.829607 -0.540140 0.000653

N 1.895595 -0.921284 0.000020

C 0.830533 -0.100034 -0.000095

C -0.440351 -0.534652 -0.000457

C 1.132743 1.375679 0.000012

H 1.721212 1.621920 0.887138

H 1.722782 1.621813 -0.886096

H 0.218445 1.965420 -0.000819

C -1.742335 -0.179054 -0.000030

N -2.889989 0.047555 0.000237

**10**


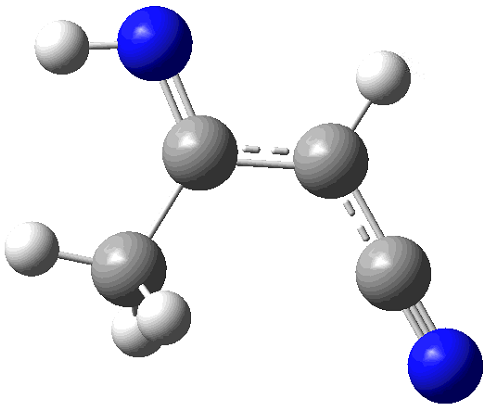


0 2

H -2.818921 -0.152458 0.000194

C -0.919402 -0.102885 -0.000113

N -2.014942 -0.784244 0.000082

C 0.297457 -0.878192 -0.000074

C 1.567326 -0.280455 0.000002

N 2.615736 0.209271 0.000063

H 0.245085 -1.960033 -0.000056

C -0.822631 1.397507 -0.000015

H -0.275437 1.739099 0.882752

H -0.275515 1.739237 -0.882771

H -1.817273 1.843117 0.000057

**(10)**


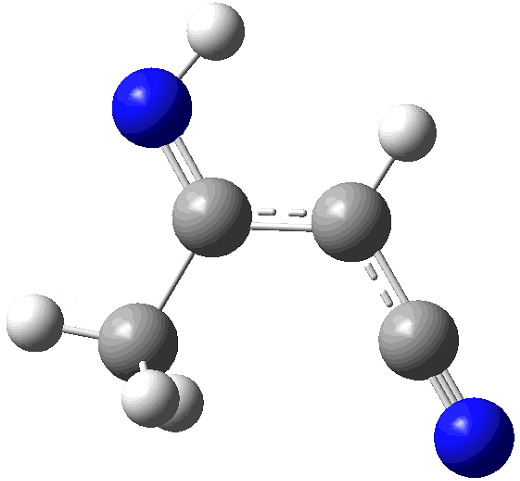


0 2

C 0.931667 -0.061013 -0.000002

N 2.102604 -0.602056 0.000008

C -0.265168 -0.870781 -0.000061

C -1.548136 -0.302061 -0.000094

N -2.605723 0.167290 0.000187

H -0.194476 -1.952861 -0.000014

C 0.789182 1.433462 -0.000041

H 0.233752 1.762305 -0.882459

H 0.233676 1.762341 0.882316

H 1.774066 1.897074 -0.000010

H 2.029548 -1.623131 -0.000011

**10'**


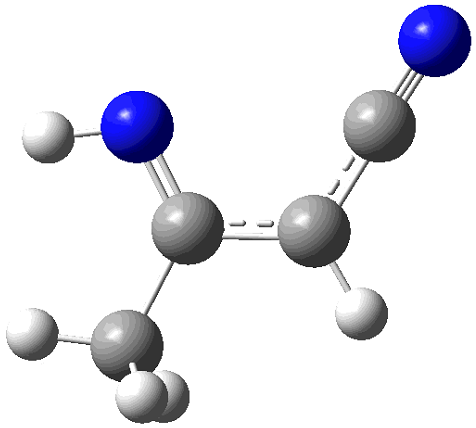


0 2

H -1.462898 1.910760 -0.000139

C -0.813841 0.126246 0.000008

N -0.581212 1.393272 -0.000113

C 0.319887 -0.769591 0.000071

C 1.634670 -0.271341 -0.000017

N 2.722480 0.119250 -0.000090

H 0.187389 -1.844276 0.000182

C -2.172276 -0.516924 0.000104

H -2.284328 -1.152703 -0.881548

H -2.284247 -1.152625 0.881824

H -2.955436 0.240849 0.000107

**(10')**


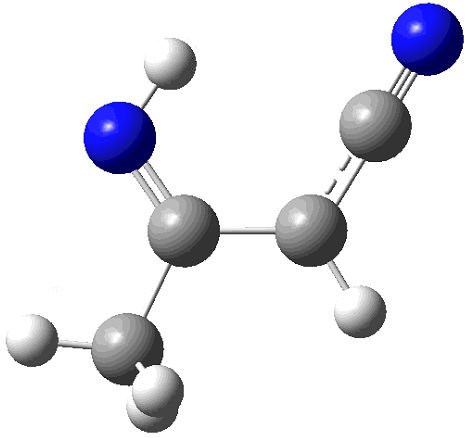


0 2

C -0.833658 0.151693 0.000009

N -0.714279 1.433847 -0.000079

C 0.286930 -0.767642 0.000054

C 1.616379 -0.313407 -0.000007

N 2.706221 0.072383 -0.000055

H 0.122977 -1.838473 0.000131

C -2.194145 -0.482931 0.000061

H -2.311761 -1.118367 -0.881340

H -2.311728 -1.118297 0.881516

H -2.965196 0.285682 0.000044

H 0.269071 1.719564 -0.000112

**1aa**


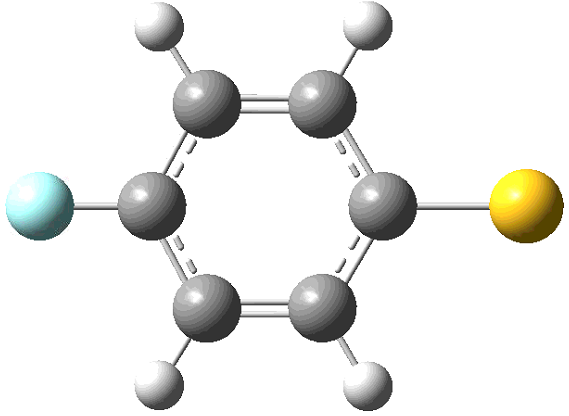


0 2

C -1.093698 1.222852 0.000125

C 0.287665 1.218364 0.000071

C 1.009259 0.000000 0.000384

C 0.287665 -1.218364 0.000071

C -1.093698 -1.222852 0.000125

C -1.751610 0.000000 0.000390

H -1.665144 2.142934 0.000018

H 0.831633 2.155216 -0.000083

H 0.831632 -2.155216 -0.000084

H -1.665144 -2.142934 0.000018

S 2.726829 0.000000 -0.000192

F -3.092859 0.000000 -0.000421

**11**


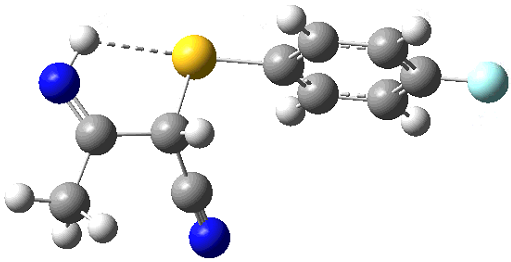


0 1

H 1.325485 -0.213126 1.276646

C 3.229665 -0.304421 0.317506

N 3.712135 -1.392213 -0.111070

C 1.729517 0.039282 0.289516

C 1.487957 1.463654 0.072261

N 1.258676 2.573566 -0.118661

S 0.805075 -0.927900 -0.973208

C -0.858494 -0.532264 -0.457073

C -1.550235 0.491101 -1.105949

C -1.464097 -1.253105 0.573437

C -2.850821 0.802804 -0.724642

H -1.075360 1.045198 -1.907227

C -2.760717 -0.945976 0.965505

H -0.926177 -2.052998 1.069231

C -3.420666 0.075439 0.303578

H -3.411875 1.591752 -1.210617

H -3.257521 -1.486508 1.761960

F -4.681228 0.371295 0.680287

C 4.100649 0.727270 0.967128

H 3.677394 1.031869 1.928590

H 5.100725 0.322898 1.113814

H 4.159802 1.619732 0.337465

H 3.005150 -2.006236 -0.520594

**11'**


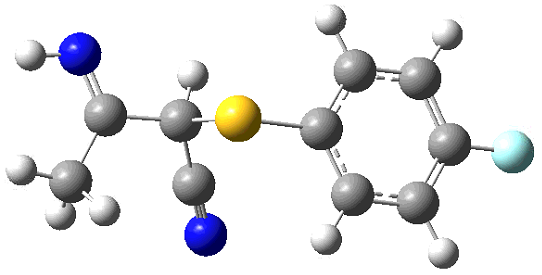


0 1

H 4.771772 -1.358293 -0.497016

H 1.454423 -0.737763 -1.384801

C 3.249622 -0.269778 -0.293029

N 3.778888 -1.329094 -0.741453

C 1.747027 -0.125076 -0.531522

S 0.884557 -0.765136 0.980361

C -0.810441 -0.447992 0.520058

C -1.490890 -1.354335 -0.295150

C -1.454505 0.695254 0.995273

C -2.814653 -1.118434 -0.643666

H -0.988946 -2.244343 -0.656704

C -2.781318 0.938134 0.657633

H -0.921711 1.395855 1.627522

C -3.425504 0.023501 -0.155087

H -3.368995 -1.803276 -1.273701

H -3.307810 1.815669 1.012927

F -4.712286 0.252485 -0.487441

C 1.340400 1.255133 -0.774446

N 0.992339 2.336304 -0.950105

C 3.929652 0.824210 0.474483

H 4.945222 0.530109 0.737458

H 3.967274 1.732443 -0.134254

H 3.371504 1.065233 1.383386

***(E)*-2aa**


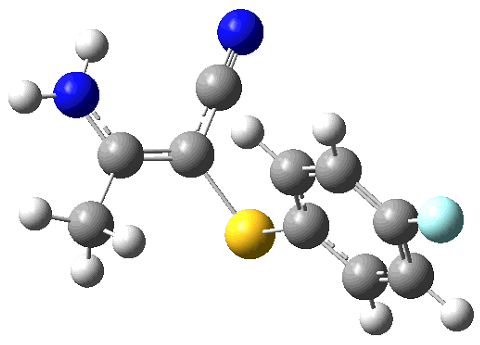


0 1

H -3.804515 -0.791647 1.412604

H -3.944335 -2.190334 0.406513

N -3.501722 -1.302405 0.593090

C -2.574758 -0.823968 -0.238533

C -1.951149 0.393855 -0.022903

C -2.224838 -1.680044 -1.417241

H -2.801728 -2.604102 -1.405888

H -2.427762 -1.143497 -2.345271

H -1.159795 -1.921719 -1.398718

C -2.275251 1.153986 1.123396

N -2.546637 1.765200 2.068019

S -0.752216 1.057816 -1.123936

C 0.806864 0.461580 -0.470731

C 0.900667 -0.477450 0.553699

C 1.967352 0.974665 -1.055790

C 2.150289 -0.906289 0.994616

H 0.006757 -0.879345 1.016248

C 3.217661 0.548715 -0.625247

H 1.900464 1.710955 -1.850042

C 3.279095 -0.384585 0.394086

H 2.247001 -1.635419 1.790292

H 4.129367 0.935572 -1.064547

F 4.494770 -0.800875 0.820212

***(Z)*-2aa**


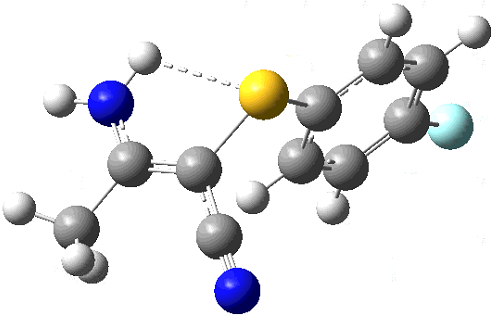


0 1

H 1.372598 0.744440 -2.266689

H 2.522456 2.035092 -2.128784

N 2.084744 1.218870 -1.726814

C 2.493788 0.746273 -0.551410

C 1.951691 -0.394991 0.011954

C 3.533982 1.550246 0.168721

H 4.124952 2.131563 -0.539449

H 3.035529 2.241562 0.854200

H 4.194331 0.908897 0.751101

C 2.360673 -0.816258 1.296423

N 2.674224 -1.181651 2.348823

S 0.729389 -1.365459 -0.806191

C -0.819568 -0.595324 -0.324381

C -1.990991 -1.253181 -0.705642

C -0.890069 0.605439 0.376986

C -3.231677 -0.712732 -0.390994

H -1.941925 -2.190999 -1.248946

C -2.129280 1.151724 0.699457

H 0.013666 1.121271 0.679811

C -3.271041 0.481363 0.306342

H -4.151931 -1.208667 -0.675575

H -2.207881 2.084148 1.245584

F -4.476185 1.012301 0.617358

**C1**


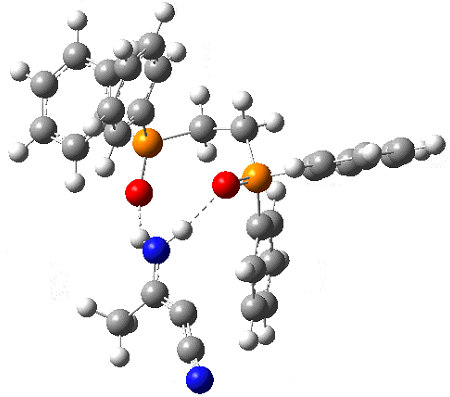


0 2

P 2.133210 0.002265 -0.219724

O 1.450758 1.332845 -0.409086

C 2.216165 -0.505886 1.520697

C 2.008119 0.467732 2.498456

C 2.083579 0.125769 3.845052

C 2.362049 -1.187293 4.216229

C 2.566088 -2.161660 3.241448

C 2.493827 -1.823645 1.893632

C 3.852982 0.089239 -0.815481

C 4.713774 -1.011187 -0.773895

C 6.020534 -0.887990 -1.231679

C 6.472834 0.332896 -1.730899

C 5.618388 1.430144 -1.770968

C 4.307968 1.310216 -1.313912

C 1.399443 -1.371097 -1.169157

C 0.147953 -2.073661 -0.633241

P -1.318217 -1.096778 -0.173399

O -1.176258 -0.294820 1.097332

C -1.784565 -0.066320 -1.594144

C -2.524711 1.089439 -1.333008

C -2.918198 1.916755 -2.379362

C -2.576840 1.589383 -3.689905

C -1.850200 0.431285 -3.954651

C -1.453252 -0.398946 -2.909800

C -2.625585 -2.351269 -0.000929

C -3.250169 -2.490997 1.238090

C -4.252994 -3.443861 1.406200

C -4.630128 -4.253651 0.339491

C -4.008353 -4.112776 -0.900772

C -3.008522 -3.162575 -1.073079

H 1.779283 1.486411 2.204080

H 1.921751 0.882905 4.603938

H 2.416859 -1.452942 5.266060

H 2.778408 -3.184919 3.529532

H 2.647092 -2.589631 1.139190

H 4.368510 -1.964168 -0.385656

H 6.685996 -1.743021 -1.200220

H 7.492360 0.426454 -2.087603

H 5.970038 2.379883 -2.157844

H 3.634026 2.159222 -1.341468

H 2.180603 -2.127188 -1.292759

H 1.225453 -0.941015 -2.160590

H -0.184404 -2.798120 -1.383731

H 0.376016 -2.647589 0.270603

H -2.794953 1.341527 -0.312677

H -3.489363 2.814539 -2.169781

H -2.880097 2.234923 -4.506515

H -1.589091 0.174239 -4.974913

H -0.889743 -1.300257 -3.128071

H -2.950433 -1.854802 2.063687

H -4.737560 -3.552425 2.369917

H -5.409906 -4.995322 0.471418

H -4.304164 -4.741366 -1.732846

H -2.531650 -3.051547 -2.042689

H 0.189360 2.463857 0.454206

H -1.016751 1.560992 1.236480

N -0.663311 2.491856 1.009191

C -1.471129 3.556748 0.970647

C -2.745218 3.546146 1.420622

C -0.906114 4.809806 0.349752

H 0.034344 5.064249 0.843901

H -0.698646 4.619005 -0.706683

H -1.598698 5.645088 0.434251

C -3.791974 4.301408 1.809140

N -4.742408 4.885230 2.167983

**C1'**


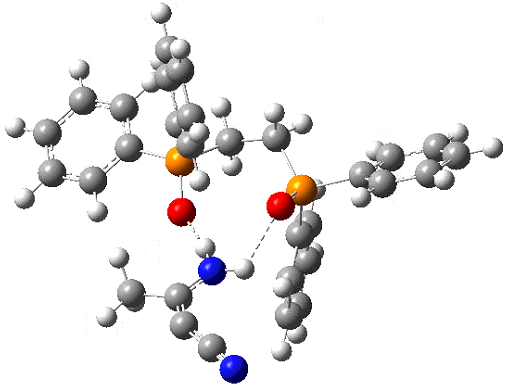


0 2

P 1.807221 -0.632203 -0.010528

O 1.359080 0.633369 -0.699082

C 1.947344 -0.416722 1.789713

C 1.350588 0.706672 2.363756

C 1.414838 0.897311 3.741988

C 2.065649 -0.034016 4.545471

C 2.653315 -1.161442 3.974222

C 2.595716 -1.354747 2.598274

C 3.439002 -1.142579 -0.634650

C 3.965888 -2.429499 -0.485213

C 5.228498 -2.725570 -0.989344

C 5.969154 -1.744134 -1.643522

C 5.446990 -0.462637 -1.795938

C 4.184033 -0.162378 -1.295661

C 0.703349 -2.052341 -0.271320

C -0.634262 -2.020124 0.474969

P -1.804899 -0.670603 0.100543

O -1.487982 0.661942 0.723501

C -1.937219 -0.577604 -1.710525

C -2.053974 0.685070 -2.292548

C -2.182656 0.804802 -3.673524

C -2.190683 -0.334735 -4.473422

C -2.067232 -1.596704 -3.895016

C -1.939386 -1.720571 -2.515548

C -3.409417 -1.297286 0.702703

C -4.151851 -0.468803 1.545145

C -5.393776 -0.887403 2.018297

C -5.894926 -2.132350 1.651933

C -5.155550 -2.962410 0.810916

C -3.915841 -2.547975 0.337190

H 0.822834 1.414257 1.734525

H 0.954069 1.771918 4.186996

H 2.115142 0.116729 5.618057

H 3.159293 -1.887855 4.599719

H 3.058863 -2.233654 2.162192

H 3.402934 -3.205816 0.021394

H 5.632043 -3.724969 -0.873974

H 6.951858 -1.980056 -2.036133

H 6.018923 0.301984 -2.309075

H 3.764958 0.829959 -1.423138

H 1.244003 -2.948262 0.046082

H 0.563055 -2.126387 -1.354212

H -1.159901 -2.957318 0.271131

H -0.483294 -1.975299 1.559183

H -2.035374 1.568119 -1.662906

H -2.274104 1.786642 -4.124112

H -2.288922 -0.240574 -5.549207

H -2.066364 -2.483664 -4.518258

H -1.834276 -2.706019 -2.072249

H -3.752919 0.499896 1.825766

H -5.967914 -0.240841 2.672294

H -6.861467 -2.458402 2.019472

H -5.544017 -3.932857 0.523875

H -3.351674 -3.203513 -0.318122

H 0.295279 2.033368 -0.339888

H -1.071779 2.919127 0.245997

N -0.157910 2.939165 -0.183341

C 0.517693 4.080969 -0.359200

C 0.034616 5.307186 -0.065480

C 1.916670 3.943082 -0.899074

H 2.521521 3.370394 -0.191454

H 1.891041 3.393217 -1.842347

H 2.372695 4.918908 -1.056690

C -1.002826 6.112873 0.221785

N -1.856745 6.878687 0.466375

**C2**


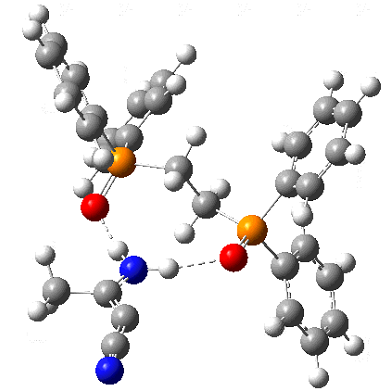


0 2

P 2.111945 -0.256662 -0.604077

P -2.145651 0.016008 0.313984

O 2.020033 0.801661 -1.681723

O -2.138780 1.518807 0.474619

C 0.601813 -0.301567 0.409895

H 0.719320 -1.046256 1.201105

H 0.530009 0.680529 0.891265

C -0.621836 -0.595908 -0.465031

H -0.713570 -1.663618 -0.681768

H -0.571292 -0.070660 -1.426648

C 3.475046 0.029122 0.559018

C 3.428902 1.189634 1.340389

H 2.586276 1.870626 1.258165

C 4.466378 1.476849 2.219563

H 4.428781 2.375486 2.824456

C 5.553421 0.610036 2.320688

H 6.361923 0.834768 3.007112

C 5.605380 -0.539949 1.539453

H 6.453360 -1.210948 1.612942

C 4.568488 -0.832403 0.657022

H 4.618250 -1.728226 0.048038

C 2.305456 -1.920982 -1.290922

C 2.536147 -2.053611 -2.659463

H 2.602941 -1.166275 -3.279626

C 2.672061 -3.322335 -3.218670

H 2.853150 -3.426903 -4.282237

C 2.573915 -4.451686 -2.412158

H 2.680097 -5.438620 -2.848502

C 2.337686 -4.320049 -1.044046

H 2.261559 -5.201480 -0.417854

C 2.202485 -3.056518 -0.481041

H 2.023452 -2.955821 0.585551

C -3.515651 -0.543846 -0.736016

C -3.707038 -1.897765 -1.026356

H -3.034776 -2.646529 -0.619089

C -4.764169 -2.287196 -1.840411

H -4.913474 -3.336792 -2.066225

C -5.630511 -1.328316 -2.364216

H -6.455435 -1.634539 -2.997667

C -5.440734 0.019477 -2.074887

H -6.115769 0.763522 -2.482095

C -4.382289 0.415115 -1.260439

H -4.224903 1.462812 -1.028670

C -2.294559 -0.839492 1.907685

C -2.844659 -0.135091 2.979930

H -3.148382 0.897537 2.845028

C -2.994188 -0.758186 4.215713

H -3.423370 -0.211307 5.047446

C -2.589528 -2.079640 4.382496

H -2.704738 -2.562909 5.346269

C -2.032597 -2.781762 3.315649

H -1.711037 -3.808353 3.448074

C -1.883736 -2.164849 2.077779

H -1.445531 -2.717261 1.252550

H 0.764660 2.157093 -1.274461

C 0.325939 4.132809 -0.985194

N 0.076497 2.819148 -0.911939

C -0.489616 5.083102 -0.485490

C -0.702775 6.404218 -0.326767

N -0.959162 7.533173 -0.147488

H -0.750269 2.465292 -0.427536

C 1.605659 4.528563 -1.679499

H 1.592483 4.142418 -2.701606

H 1.728509 5.609512 -1.703822

H 2.452661 4.080072 -1.154030

**C2'**


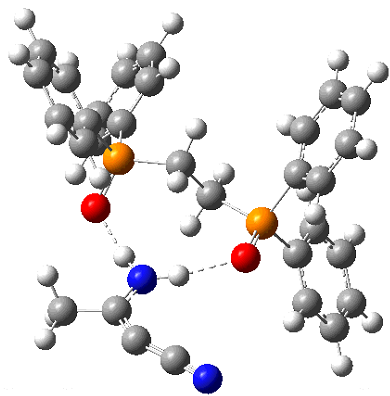


0 2

P 2.185450 -0.067933 -0.595456

P -2.086367 -0.111516 0.277622

O 1.988345 0.901722 -1.740519

O -2.185858 1.391843 0.394549

C 0.673057 -0.212184 0.406733

H 0.842221 -0.911891 1.229067

H 0.515786 0.779221 0.847920

C -0.513350 -0.639000 -0.464645

H -0.519556 -1.718559 -0.636087

H -0.490991 -0.150874 -1.446466

C 3.477022 0.469329 0.559959

C 3.295178 1.713159 1.176458

H 2.403926 2.299789 0.970259

C 4.257180 2.203822 2.050548

H 4.112921 3.166616 2.527295

C 5.404565 1.456897 2.312044

H 6.154232 1.840022 2.995241

C 5.590738 0.222925 1.696558

H 6.485688 -0.354808 1.897215

C 4.629147 -0.273679 0.818957

H 4.784070 -1.234004 0.339450

C 2.587909 -1.734736 -1.176477

C 2.892902 -1.903493 -2.527085

H 2.893533 -1.042596 -3.186639

C 3.185371 -3.173358 -3.018139

H 3.422656 -3.304974 -4.067534

C 3.168621 -4.270203 -2.162115

H 3.394951 -5.258960 -2.545148

C 2.858486 -4.103610 -0.812868

H 2.843981 -4.959909 -0.148597

C 2.566376 -2.838126 -0.317501

H 2.326962 -2.711819 0.734126

C -3.398723 -0.794722 -0.771981

C -3.463106 -2.159937 -1.064738

H -2.724402 -2.845164 -0.660574

C -4.481030 -2.644482 -1.877781

H -4.530906 -3.702745 -2.106845

C -5.434619 -1.770107 -2.397820

H -6.227110 -2.150819 -3.032190

C -5.371033 -0.411039 -2.105871

H -6.112172 0.268810 -2.510413

C -4.352339 0.080019 -1.293042

H -4.292784 1.137712 -1.060412

C -2.195348 -0.927418 1.895635

C -2.775090 -0.219946 2.950056

H -3.129060 0.792522 2.787803

C -2.888913 -0.813516 4.204238

H -3.341002 -0.264189 5.022113

C -2.420465 -2.108471 4.407020

H -2.508667 -2.568793 5.384767

C -1.834174 -2.813320 3.357695

H -1.462737 -3.819074 3.517186

C -1.720183 -2.225823 2.102087

H -1.256983 -2.779493 1.291427

H 0.633435 2.169780 -1.392003

C 0.055780 4.125948 -1.249016

N -0.100353 2.804987 -1.070850

C -0.810906 5.057276 -0.803542

C -1.997667 5.387265 -0.261972

N -3.011419 5.752115 0.199649

H -0.895100 2.425803 -0.553186

C 1.300029 4.553967 -1.980423

H 1.356095 4.032642 -2.938868

H 1.305291 5.628808 -2.151442

H 2.179493 4.279836 -1.391629

**C3**


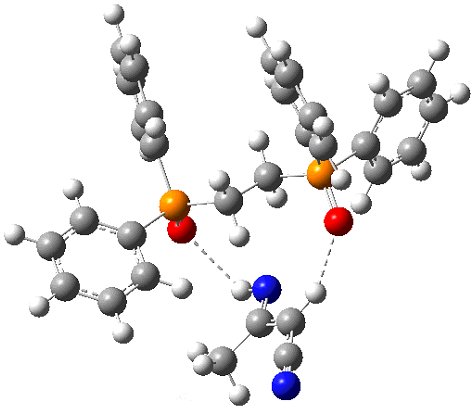


0 2

P 1.857222 -0.602016 -0.954145

P -2.142856 0.737198 0.352944

O 1.967983 0.011213 -2.326006

O -1.853630 2.151518 0.791597

C 0.518998 0.078390 0.077048

H 0.498045 -0.494239 1.010215

H 0.761876 1.113664 0.324750

C -0.817218 0.012511 -0.658955

H -1.097226 -1.014921 -0.912417

H -0.771557 0.593538 -1.583967

C 3.372623 -0.420905 0.036978

C 3.629496 0.795992 0.677146

H 2.902861 1.601900 0.643456

C 4.825347 0.987184 1.361468

H 5.017204 1.932426 1.856744

C 5.770450 -0.034814 1.410994

H 6.701984 0.114585 1.945238

C 5.520713 -1.247025 0.773534

H 6.255716 -2.043017 0.811038

C 4.325665 -1.441342 0.086760

H 4.139409 -2.389169 -0.407811

C 1.504551 -2.384035 -1.040303

C 1.440516 -2.984940 -2.296470

H 1.625010 -2.387652 -3.183023

C 1.140154 -4.341435 -2.404504

H 1.091088 -4.807917 -3.381827

C 0.899294 -5.093158 -1.258983

H 0.661926 -6.147821 -1.342929

C 0.961156 -4.493826 -0.001267

H 0.772270 -5.079621 0.891224

C 1.264898 -3.141846 0.110313

H 1.320259 -2.681628 1.092426

C -3.636653 0.609656 -0.675887

C -4.765128 -0.119758 -0.298831

H -4.778085 -0.667345 0.637134

C -5.885322 -0.143973 -1.127305

H -6.760270 -0.711845 -0.831985

C -5.881967 0.558893 -2.327873

H -6.755633 0.538798 -2.969605

C -4.758337 1.292595 -2.705438

H -4.755548 1.843769 -3.638691

C -3.638504 1.318321 -1.882819

H -2.766043 1.894696 -2.177146

C -2.344991 -0.379480 1.771366

C -2.440692 0.189932 3.041257

H -2.412364 1.269166 3.145575

C -2.562386 -0.626528 4.163032

H -2.638433 -0.181953 5.148801

C -2.580408 -2.010006 4.016740

H -2.669210 -2.645884 4.890459

C -2.480142 -2.582130 2.748999

H -2.491845 -3.660139 2.635736

C -2.362867 -1.770439 1.626201

H -2.288278 -2.222051 0.641602

H 1.343960 2.331942 -2.645646

H -0.319251 3.565559 0.233151

C 1.414738 3.454276 -1.103017

N 0.745295 2.690883 -1.898657

C 0.693397 3.923107 0.055215

C 1.282191 4.779319 0.999225

N 1.763883 5.487549 1.778180

C 2.853335 3.857289 -1.288993

H 2.906668 4.914788 -1.564200

H 3.421510 3.726933 -0.365117

H 3.311949 3.263874 -2.080360

**C4**


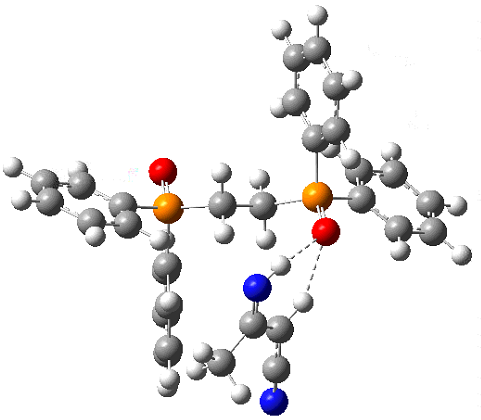


0 2

P 2.224826 0.117417 0.430847

C 3.530595 -0.815171 -0.417368

C 4.158715 -1.857219 0.265148

C 5.162638 -2.589818 -0.364038

C 5.534696 -2.283644 -1.669560

C 4.904556 -1.244309 -2.352924

C 3.903637 -0.509136 -1.729235

C 2.760049 1.851782 0.507900

C 3.232745 2.330567 1.731086

C 3.676275 3.645562 1.837665

C 3.643733 4.483119 0.726262

C 3.163667 4.009660 -0.493422

C 2.720630 2.696105 -0.605850

C 0.786183 0.067877 -0.677306

H 3.861308 -2.088534 1.282329

H 5.653192 -3.398627 0.165522

H 6.316978 -2.853952 -2.157789

H 5.193386 -1.007096 -3.370415

H 3.416931 0.301191 -2.262774

H 3.246597 1.674207 2.594571

H 4.043514 4.015735 2.788080

H 3.988090 5.507720 0.810022

H 3.131593 4.664357 -1.356661

H 2.343046 2.337429 -1.557847

H 0.552794 -0.992148 -0.828377

H 1.077199 0.483126 -1.647153

C -0.400832 0.821810 -0.077604

P -1.768780 0.898037 -1.273499

H -0.141877 1.856110 0.172968

H -0.758809 0.328164 0.830579

O -1.383005 1.481079 -2.605383

C -3.102399 1.830359 -0.462656

C -2.346738 -0.819661 -1.431464

C -3.934498 2.597182 -1.281842

C -3.323769 1.795536 0.917463

C -2.270418 -1.431999 -2.681821

C -2.835128 -1.530667 -0.331387

C -4.988321 3.316268 -0.726588

H -3.749904 2.631708 -2.350326

C -4.378204 2.517719 1.467972

H -2.681935 1.209717 1.568103

C -2.682275 -2.754401 -2.833390

H -1.890765 -0.870645 -3.528780

C -3.251667 -2.847797 -0.488921

H -2.898052 -1.056665 0.644758

C -5.210652 3.275271 0.647324

H -5.631419 3.911473 -1.364908

H -4.547295 2.491626 2.538303

C -3.173078 -3.459916 -1.739230

H -2.620741 -3.230764 -3.805335

H -3.634704 -3.400376 0.361591

H -6.030259 3.837100 1.081165

H -3.496270 -4.488137 -1.857581

O 1.942088 -0.440287 1.806939

H 0.230172 -0.474255 2.810499

H 0.639962 -2.280968 1.612496

C -1.115953 -1.823780 2.851582

N -0.689885 -0.665664 3.228982

C -0.326702 -2.639760 1.952986

C -0.750064 -3.913186 1.539590

N -1.100359 -4.963029 1.200992

C -2.436731 -2.350759 3.333467

H -3.082663 -2.606753 2.489427

H -2.287457 -3.261954 3.919699

H -2.928371 -1.601655 3.952321

**C5**


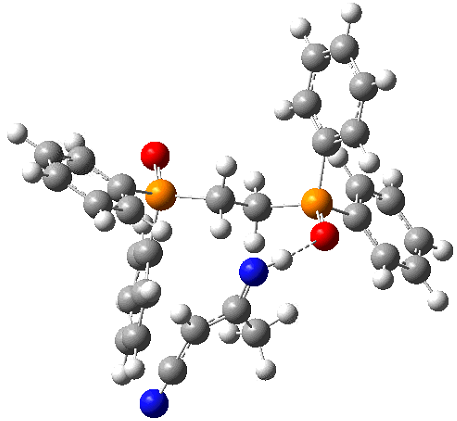


0 2

P 2.335462 -0.075445 0.504387

C 3.615099 -1.170677 -0.174286

C 3.948127 -2.315469 0.549910

C 4.911476 -3.191927 0.056437

C 5.539297 -2.924233 -1.156128

C 5.206568 -1.780270 -1.880764

C 4.245121 -0.902382 -1.392581

C 3.000948 1.616448 0.497527

C 3.501148 2.110048 1.704816

C 4.036980 3.392707 1.763891

C 4.068523 4.187392 0.620910

C 3.562659 3.701318 -0.582625

C 3.027530 2.418597 -0.647513

C 0.969435 -0.107856 -0.695669

H 3.455154 -2.512178 1.495735

H 5.171182 -4.080837 0.620042

H 6.290008 -3.606360 -1.539140

H 5.698573 -1.572551 -2.824044

H 3.991454 -0.009933 -1.956659

H 3.463963 1.490582 2.594550

H 4.424202 3.772833 2.702420

H 4.482250 5.188381 0.668858

H 3.579080 4.322300 -1.470923

H 2.631942 2.052537 -1.589209

H 0.694493 -1.160880 -0.818822

H 1.340808 0.252448 -1.659804

C -0.210978 0.717803 -0.185480

P -1.505716 0.898031 -1.449796

H 0.089796 1.734634 0.091204

H -0.649780 0.244934 0.696509

O -1.029285 1.529778 -2.729021

C -2.843273 1.837723 -0.651607

C -2.154454 -0.779137 -1.730861

C -3.591233 2.700944 -1.455345

C -3.151403 1.710503 0.706740

C -1.985616 -1.351746 -2.991897

C -2.795886 -1.496885 -0.718078

C -4.647538 3.423187 -0.908191

H -3.340858 2.807110 -2.505594

C -4.207827 2.435267 1.249201

H -2.573813 1.053678 1.349544

C -2.457112 -2.638912 -3.238807

H -1.489590 -0.785199 -3.772652

C -3.264901 -2.781901 -0.967296

H -2.931813 -1.054815 0.265542

C -4.956529 3.289408 0.442770

H -5.226587 4.091438 -1.535334

H -4.442294 2.336001 2.303055

C -3.096048 -3.352080 -2.228621

H -2.326927 -3.082824 -4.219352

H -3.762629 -3.336430 -0.179433

H -5.778163 3.853454 0.869840

H -3.464781 -4.353187 -2.422038

O 1.923692 -0.501154 1.893239

H 0.235984 -0.313229 2.898238

H -3.255863 -0.539837 3.650311

C -1.370843 -1.327600 2.845662

N -0.748063 -0.251585 3.191932

C -2.782776 -1.371795 3.142675

C -3.581880 -2.470687 2.787775

N -4.237976 -3.375560 2.487207

C -0.740177 -2.504594 2.149854

H -0.882377 -3.412710 2.742333

H -1.214133 -2.671379 1.178168

H 0.324912 -2.324907 2.005541
